# A Circular RNA Generated from Nebulin (*NEB*) Gene Splicing Promotes Skeletal Muscle Myogenesis in Cattle as Detected by a Multi‐Omics Approach

**DOI:** 10.1002/advs.202300702

**Published:** 2023-11-30

**Authors:** Kongwei Huang, Zhipeng Li, Dandan Zhong, Yufeng Yang, Xiuying Yan, Tong Feng, Xiaobo Wang, Liyin Zhang, Xinyue Shen, Mengjie Chen, Xier Luo, Kuiqing Cui, Jieping Huang, Saif Ur Rehman, Yu Jiang, Deshun Shi, Alfredo Pauciullo, Xiangfang Tang, Qingyou Liu, Hui Li

**Affiliations:** ^1^ State Key Laboratory for Conservation and Utilization of Subtropical Agro‐Bioresources, Guangxi Key Laboratory of Animal Breeding, Disease Control and Prevention, College of Animal Science and Technology Guangxi University Nanning 530005 China; ^2^ Guangdong Provincial Key Laboratory of Animal Molecular Design and Precise Breeding, School of Life Science and Engineering Foshan University Foshan 528225 China; ^3^ School of Biology and Biological Engineering South China University of Technology Guangzhou 510641 China; ^4^ Key Laboratory of Animal Genetics, Breeding and Reproduction of Shaanxi Province, College of Animal Science and Technology Northwest A&F University Yangling 712100 China; ^5^ Department of Agricultural, Forest and Food Sciences University of Torino Grugliasco (TO) 10095 Italy; ^6^ State Key Laboratory of Animal Nutrition and Feeding，Institute of Animal Sciences Chinese Academy of Agricultural Sciences Beijing 100193 China

**Keywords:** Cattle, circNEB, myogenesis, Ribo‐seq, SCF complex, skeletal muscle

## Abstract

Cattle and the draught force provided by its skeletal muscle have been integral to agro‐ecosystems of agricultural civilization for millennia. However, relatively little is known about the cattle muscle functional genomics (including protein coding genes, non‐coding RNA, etc.). Circular RNAs (circRNAs), as a new class of non‐coding RNAs, can be effectively translated into detectable peptides, which enlightened us on the importance of circRNAs in cattle muscle physiology function. Here, RNA‐seq, Ribosome profiling (Ribo‐seq), and peptidome data are integrated from cattle skeletal muscle, and detected five encoded peptides from circRNAs. It is further identified and functionally characterize a 907‐amino acids muscle‐specific peptide that is named circNEB‐peptide because derived by the splicing of Nebulin (*NEB*) gene. This peptide localizes to the nucleus and cytoplasm and directly interacts with SKP1 and TPM1, key factors regulating physiological activities of myoblasts, via ubiquitination and myoblast fusion, respectively. The circNEB‐peptide is found to promote myoblasts proliferation and differentiation in vitro, and induce muscle regeneration in vivo. These findings suggest circNEB‐peptide is an important regulator of skeletal muscle regeneration and underscore the possibility that more encoding polypeptides derived by RNAs currently annotated as non‐coding exist.

## Graphical abstract

CircNEB is a circular RNA that encode a protein in bovine skeletal muscle filtered through RNA‐seq, Ribo‐seq, and LC‐MS‐MS. The circNEB‐protein promote myoblast proliferation through the ubiquitination and activates PI3K‐Akt pathway to regulates cell differentiation. The expression of circNEB in vivo have shown that it promotes muscle repair in mice and tree shrews, as well as stimulates the regeneration of muscle in aging rabbits.

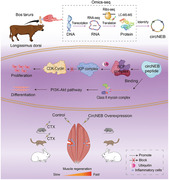

## 1 Introduction

Cattle plays a key role in global food systems as, often, it is the main source of animal protein (milk and meat). It contributes to crop productivity through the provision of draught power and manure, and often it represents the only livelihood and nutrition of poor households in low‐ and middle‐income countries. Beef is of excellent quality and rich in protein, making it a staple of the human diet.^[^ ^1^ ^]^ In addition to its protein content, beef also contains vitamins and minerals that are essential for human health.^[^ ^2^ ^]^

Muscle mass makes up 40% of a mammal's body weight.^[^ ^3^ ^]^ The skeletal muscle distributed in the limbs of animals consists mainly of a large number of muscle fibers with the ability to contract.^[^ ^4^ ^]^ The structural proteins of myofibers include nebulin, myosin, troponin, actin, etc., which are the basic components of both thick and thin myofilaments. The development and growth of skeletal muscle the primary target of agricultural meat production is a complex process, and the regulatory mechanisms underlying differences in meat quality are still poorly understood.^[^ ^5^ ^]^ This process relies on the co‐regulation of coding genes and non‐coding RNAs.^[^ ^6^ ^]^ Abnormalities in these regulatory networks can lead to a variety of human muscle diseases, such as Duchenne muscular dystrophy disease, Rippling muscle disease, hypertrophic cardiomyopathy, and so on.^[^ ^7^ ^]^

Among of the most important non‐coding RNAs, circRNAs play an increasingly important role in muscle development and in the treatment of muscular diseases.^[^ ^8^ ^]^ CircRNAs can act directly to influence the proliferation and differentiation of skeletal muscle satellite cells, such as circSVIL, or they can act as sponges for miRNAs to regulate muscle development, such as circMYBPC1.^[^ ^9^ ^]^ Most circRNAs are exon‐derived and present in the cytoplasm, suggesting that circRNA has the potential to be translated into protein. It has been shown that m6A methylation modifications in circRNAs, as well as internal ribosome entry site (IRES) structures, can facilitate translation of circRNAs, strongly supporting the ability of circRNAs to encode peptides.^[^ ^8^, ^10^ ^]^ The codability of circular circRNAs in skeletal muscle has also been demonstrated. For example, circ‐ZNF609 encoding peptide is significantly downregulated in Duchenne muscular dystrophy patients and circFAM188B promotes the proliferation and differentiation of chicken skeletal muscle satellite cells by encoding a protein.^[^ ^7d^ ^]^

Goal of this study was to explore the role of coding capacity played by circRNAs in cattle skeletal muscle development. To this end, we integrated RNA‐seq, Ribo‐seq and peptidome analyses to reveal the translation of circRNAs associated with myogenesis. We identified 5 circRNAs with potential coding capacity. We discovered a previously unrecognized ORF encoding a conserved 907‐amino acids (99 kDa) polypeptide, which we named circNEB‐peptide because it is derived from the NEB gene (encoding a nebulin protein). The bovine *NEB* gene is located on chromosome 2 and contains 173 exons that encode huge muscle structural proteins, known as the ruler of the fine myofilaments of muscle fibers. Through our studies we characterized that circNEB‐peptide localizes to the nucleus and cytoplasm, where it promotes myoblast proliferation and fusion associated with *SKP1* and *TPM1*, key factors regulating muscle cell cycle and differentiation, respectively. These findings demonstrate that the circNEB‐peptide may control the critical step in myofiber formation during muscle development. We further investigated the circNEB‐peptide in vivo to promote the recovery of damaged muscles caused by Cardiotoxin (*CTX*) both in mice and tree shrews; and we explored the contribution of circNEB‐peptide to muscle regeneration in four ageing rabbits.

## 2 Result

### 2.1 Screening and Coding Ability Verification of circRNAs with Coding Potential

#### 2.1.1 Multiomics Screening and Expression verification of circRNAs with Coding Potential

**Figure 1:**
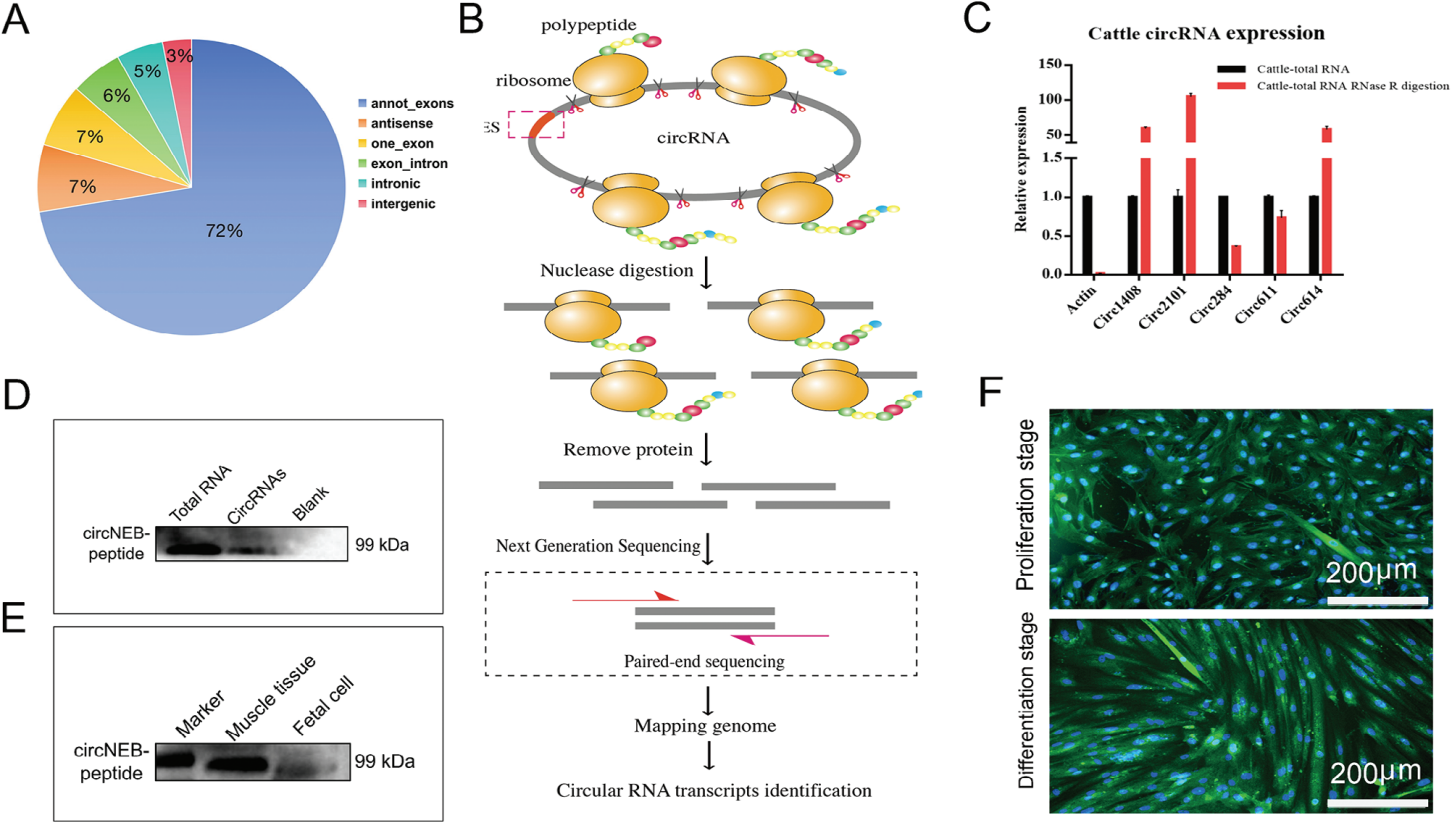
Transcriptome and translatome identification of translatable transcripts, qPCR validation, and bos‐circNEB encoded polypeptide detection. A) Annotation, classification, and distribution ratio of circRNA sequences. B) Library construction and sequencing workflow of Ribo‐seq. C) Total RNA after RNase R digestion: five candidate coding circRNAs in cattle muscle were analyzed by qPCR. As the linear control mRNA, the expression of actin decreased significantly after digestion (*n* = 3). D) Digestion of the linear transcript, circRNAs were translated in vitro and bos‐circNEB polypeptide was detected by western blotting. E) Both cattle muscle tissue and fetal cattle myoblast expressed bos‐circNEB peptide as detected by western blotting. The band size of the bos‐circNEB polypeptide was 99 kDa. F) Immunofluorescence were used to detect the expression of bos‐circNEB encoded peptides during the proliferation and 5 days after differentiation of myoblasts (*n* = 6). Data are represented as the mean ± SEM and analyzed by Student's *t*‐test. ^*^ *p* < 0.05, ^**^ *p* < 0.01, ^***^ *p* < 0.001 for groups connected by horizontal lines. *p*‐values < 0.05 were considered statistically significant.

In order to obtain a comprehensive picture of the circRNAs’ expression profiles in the bovine muscles, we collected and sequenced circRNAs from the *Longissimus dorsi* of adult cattle. We observed that 2150 circRNAs were mostly located in the exon regions (**Figure** **1**A), and predicted to contain 1869 ORFs. To assess the coding potential of circRNAs, we searched for IRES sequences of circRNAs by IRESfinder. Results showed that 1373 circRNAs expressed in cattle contained IRES sequences, which accounted for 63.9% of the identified circRNAs (Table S1, Supporting Information). Those circRNAs with IRES sequences served as candidate circRNAs for subsequent analysis because more likely they could encode for polypeptides.

Ribo‐seq is a robust technique for studying translation transcripts in recent years.^[^ ^11^ ^]^ We performed Ribo‐seq to identify the ribosome bounds of circRNAs. A schematic representation of the Ribo‐seq workflow was shown in Figure 1B. By sequencing of ribosome bound fragments, circRNA‐ORFs transcripts were identified. The ribosome binding transcripts were aligned with the predicted circRNA‐ORF, thus the Ribo‐seq data were obtained by a conventional bioinformatics analysis, as shown in Figure S1 (Supporting Information).

In order to understand the proteins and peptides encoded by cattle skeletal muscle, we analyzed the cattle muscle protein by label‐free mass spectrometry. Aligning the circRNA‐peptides databases, we observed 92 circRNAs derived small peptides, and the mass spectrometry mapping results were listed in Table S2 (Supporting Information).

Based on the analysis of transcriptome, translatome and protein mass spectrometry, as well as the analysis of whether circRNAs host genes are associated with myocyte proliferation, differentiation and muscle fiber formation muscle growth, we candidated five circRNAs with coding potential: circ611, circ614, circ2101, circ1480, and circ284. Among these candidate circRNAs, the circ1480 source gene is ARHGAP10, which encodes a protein with GTPase activator activity that is involved in the negative regulation of cytoskeletal organization and apoptotic processes. Circ2101 source gene is RBBP7, which is a ubiquitously expressed nuclear protein and it plays a role in the regulation of cell proliferation and differentiation. As for the circ284 that was the focus of this study, its source gene NEB encodes nebulin protein, which constitutes ≈ 4% of the total protein in muscle fibers. Mutations in the NEB gene lead to human congenital myopathies. Therefore, we speculate that the circNEB‐encoded protein may regulate muscle cells and consequently affect muscle development processes. This study involved multi‐omics screening of circRNA‐encoded proteins and analysis of the functional roles of their source genes, ultimately identifying candidate circRNAs in cattle skeletal muscle. Subsequently, the presence of these five circRNAs in cattle muscle was confirmed by qPCR and resistance to RNase R treatment (Figure 1C).

#### 2.1.2 Rolling‐Translated bos‐circNEB and Validation of its Encoded Polypeptide

Analyzing the function of the source gene of circRNAs, we found that the *NEB* gene produced a circRNA that may encode a polypeptide. It is known that *NEB* gene encodes a giant protein component of the cytoskeletal matrix that coexists with the thick and thin filaments within the sarcomeres of skeletal muscle. Nebulin is made of 6669 amino acids, is conserved in different species (Figure S2, Supporting Information), and it plays a significant role for the implementation of muscle physiological performance.^[^ ^12^ ^]^ Bos‐circNEB (circ284) consists of 4 exons (from the exon 67 to 70) of the *NEB* gene (Figure S3A, Supporting Information). Notably, the rolling‐translated circNEB polypeptide sequence is highly homologous to the NEB protein partial sequence (Figure S3B, Supporting Information), and its secondary structure was predicted (Figure S3C, Supporting Information). In order to verify the authenticity of circNEB encoded polypeptide, we prepared circNEB peptides antibody. Subsequently, the protein solution of in vitro translated circRNA was detected with circNEB peptides antibody by western blotting. We observed that the size of circNEB encoded polypeptide was 99 kDa (Figure 1D). Furthermore, we also detected the same size of circNEB coding polypeptide in cattle dorsal muscle and fetal cattle myoblasts (Figure 1E). Immunofluorescence analysis revealed that circNEB polypeptide was present in myoblasts, with expression in both the proliferation and differentiation phases. Fluorescence signals were detected both in the cytoplasm and nucleus (Figure 1F). These findings suggest that the polypeptide encoded by circNEB widely existed in the proliferation and differentiation phases of myoblast, and its function of regulating muscle development is worthy of further exploration.

### 2.2 Effects of circNEB on the Proliferation and Differentiation of Cattle Myoblasts and C2C12 Cells

#### 2.2.1 Bos‐circNEB Promotes the Proliferation of Cattle Myoblasts

**Figure 2:**
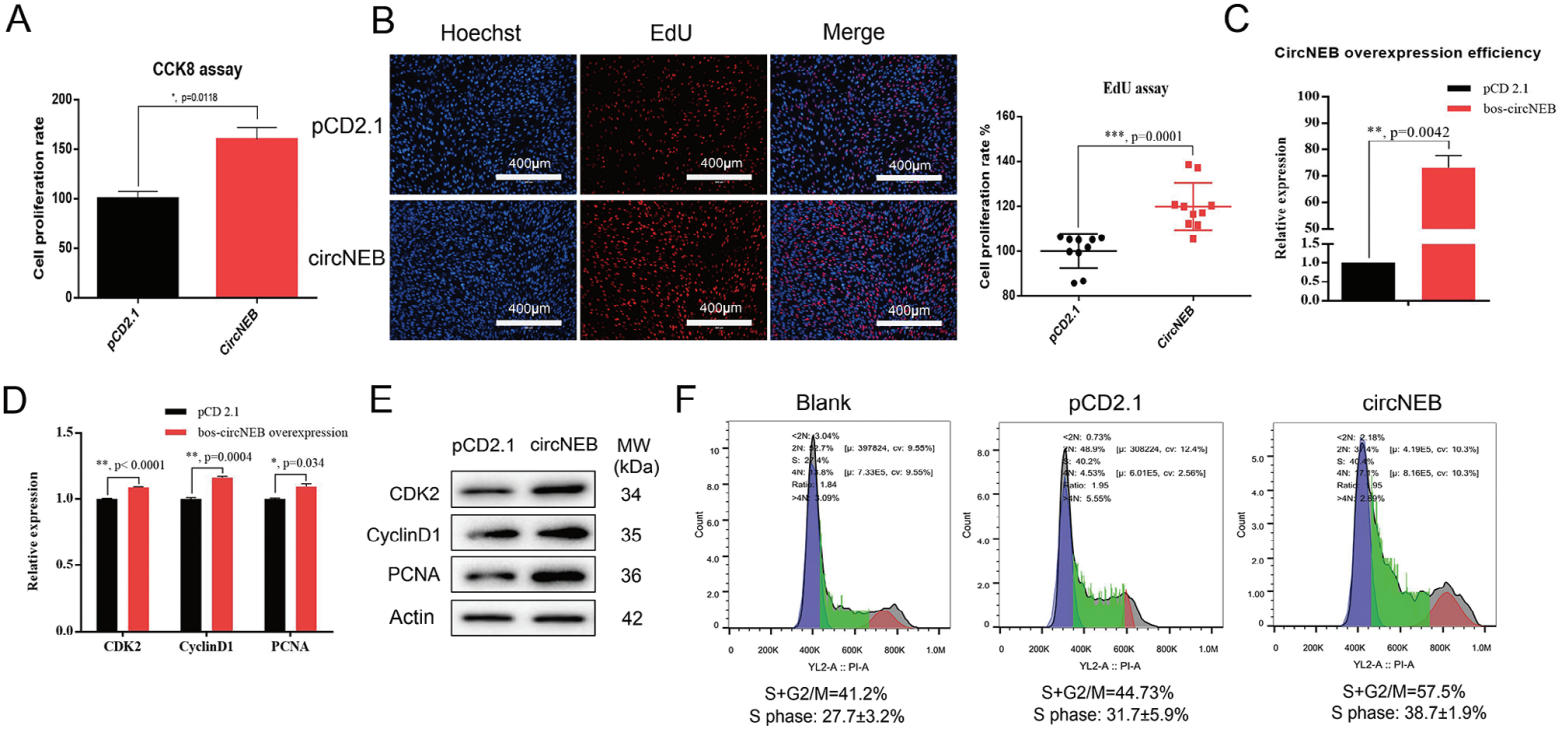
CircNEB promotes the proliferation of cattle fetal myoblasts. A) The proliferation of myoblasts after over‐expression of bos‐circNEB was measured by CCK8 assay. The cell proliferation rate of circNEB group was significantly higher than that of pCD2.1 control group (*n* = 6). B) EdU experiments detected the efficiency of myoblast proliferation. Hoechst‐stained nuclei are blue, EdU incorporated in replicated nuclei are red, and merged shows the proportion of replicating nuclei. Statistics of EdU data at 10× magnification, the percentage of red fluorescent nuclei within fields were counted. Each dot represents a different field of view (*n* = 10). C) Detection of bos‐circNEB over‐expression in cattle myoblasts by qPCR (*n* = 3). D) Gene expression of cattle myoblast in proliferation stage overexpressing bos‐circNEB detected by qPCR (*n* = 3). *CDK2*, *CyclinD1* and *PCNA* transcripts were significantly increased. E) Expressions of CDK2, CyclinD1 and PCNA proteins in cattle myoblasts overexpressing bos‐circNEB were detected by Western blotting. F) Flow cytometry analysis of the effect of bos‐circNEB on the cell cycle of cattle myoblasts (*n* = 3). The ratio of S and G2/M phase were higher in the circNEB group than control group, and the proportion of cells in the proliferative phase increased. Data are represented as the mean ± SEM and analyzed by Student's *t*‐test. ^*^, ^**^, ^***^ represent *p* < 0.05, < 0.01, < 0.001, respectively. *p*‐values < 0.05 were considered statistically significant.

To explore whether bos‐circNEB affects myoblast cell proliferation, we examined the rate of myocyte proliferation after over‐expression of circNEB on embryonic cattle myoblasts (**Figure** **2**A). The results of Cell Counting Kit‐8 (CCK8) and 5‐Ethynyl‐2′‐deoxyuridine (EdU) experiments showed that circNEB significantly promoted myoblast proliferation (Figure 2B,C). Subsequently, we detected the expression of the following proliferation key genes *CDK2*, *PCNA* and *CyclinD1*, and their mRNA and protein were significantly up‐regulated (Figure 2D,E). By flow cytometry, we observed that the cell ratio of S phase and G2/M phase in circNEB group were significantly higher than that in control group (Figure 2F). In contrast, after reducing circNEB expression by knocking down the 5′ and 3′ flanking introns by Cas9 system, EdU assay showed that myoblast proliferation was significantly inhibited (Figure S4, Supporting Information). Those results revealed that circNEB promotes the proliferation of cattle myoblasts.

#### 2.2.2 Over‐Expression of bos‐circNEB Promotes Myotube Formation and Fusion of Cattle Myoblasts

**Figure 3:**
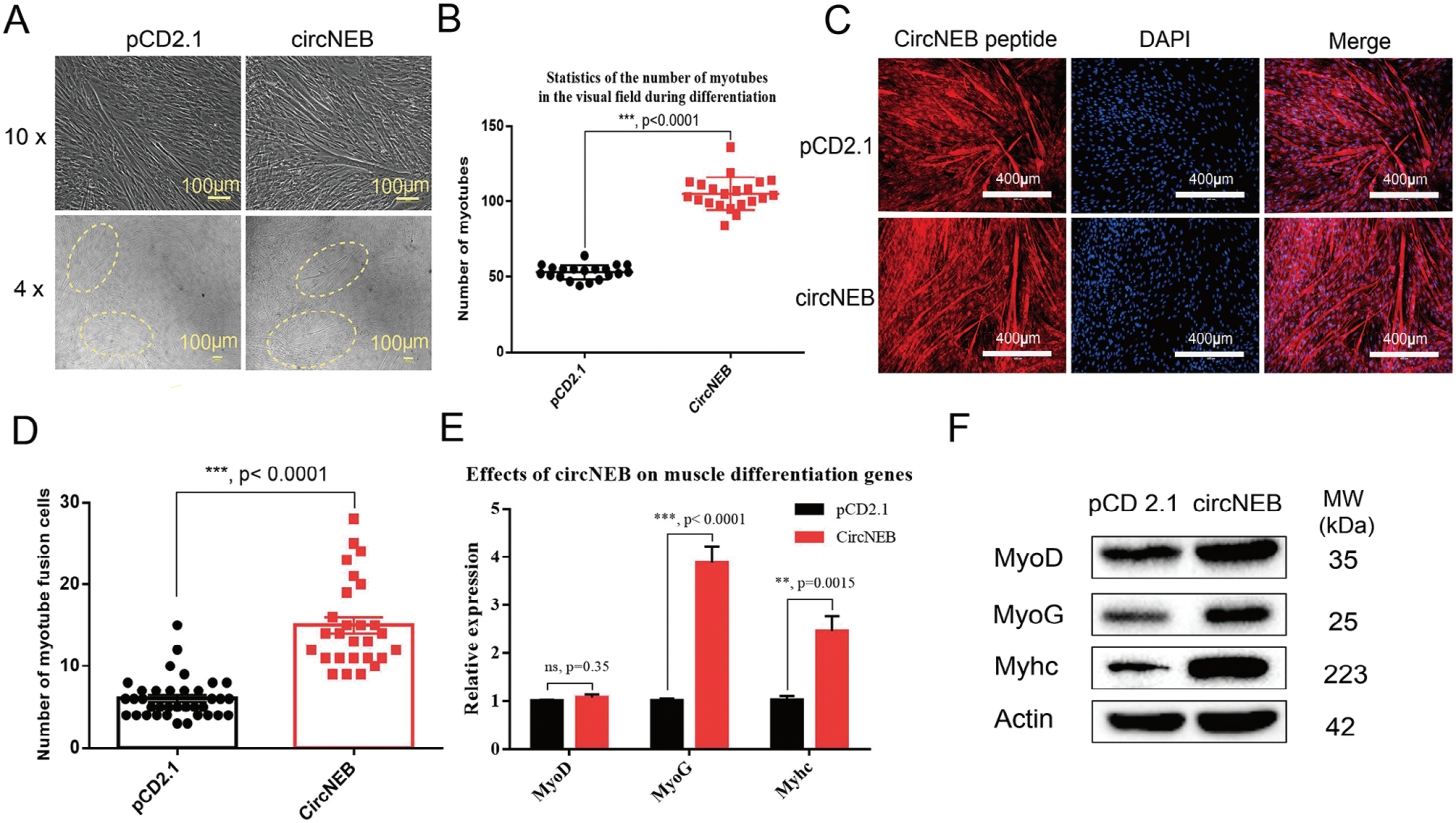
CircNEB promotes the differentiation of cattle fetal myoblasts and myotube formation. A) The bright field pictures of myoblasts induced to differentiate for 5 days were taken under 10× and 4× microscope magnification, respectively. Myotubes from the circNEB group differentiated better than those from the pCD2.1 control group. B) The number of myotubes formed in 4× microscope fields were counted separately, and each dot in the figure represents the statistical number of a visual field (*n* = 20). After statistics, the myotubes in circNEB group were significantly increased than that of control group. C) Immunofluorescence staining of bos‐circNEB encoded peptide and DAPI to visualize myotubes and nuclei, respectively. D) Statistical results of cell number of myotube fusion (pCD2.1, *n* = 36; circNEB, *n* = 27). The average number of myotube fusion cells in the circNEB group was significantly higher than that in the pCD2.1 control group. E) qPCR was used to detect the expression of muscle differentiation genes in differentiated myoblasts. The transcriptional expressions of MyoG and Myhc were significantly up‐regulated (*n* = 3). F) The expression of muscle differentiation protein was detected by western blotting. MyoD, MyoG, and Myhc proteins were significantly up‐regulated. Data are represented as the mean ± SEM and analyzed by Student's *t*‐test. ns, ^**^, ^***^ represent *p* > 0.05, < 0.01, < 0.001, respectively. *p*‐values < 0.05 were considered statistically significant.

In order to explore whether bos‐circNEB affects myoblasts to differentiate into myotubes, we induced myoblasts to differentiate after over‐expression of circNEB. When cultured in differentiation medium for the fifth day, the number of myotubes in circNEB group was significantly higher than that in control group (**Figure** **3**A,B). Then, in order to evaluate the degree of myotubes differentiation, we counted the average number of myotubes nuclei in circNEB group and pCD2.1 control group by immunofluorescence staining (Figure 3C,D). The number of myotube fusion cells in the circNEB group was significantly higher than that in the control group, suggesting that circNEB promotes myoblast fusion.

In addition, we examined the expression levels of genes critical for muscle cell differentiation. qPCR and western blotting results showed that circNEB promoted the expression of MyoD, MyoG and Myhc (Figure 3E,F). Upregulation of the expression of these myogenic differentiation related genes, may explain the phenomenon that circNEB promotes myoblast differentiation.

#### 2.2.3 Over‐Expression of mus‐circNEB Promotes C2C12 Cell Proliferation and Differentiation

**Figure 4:**
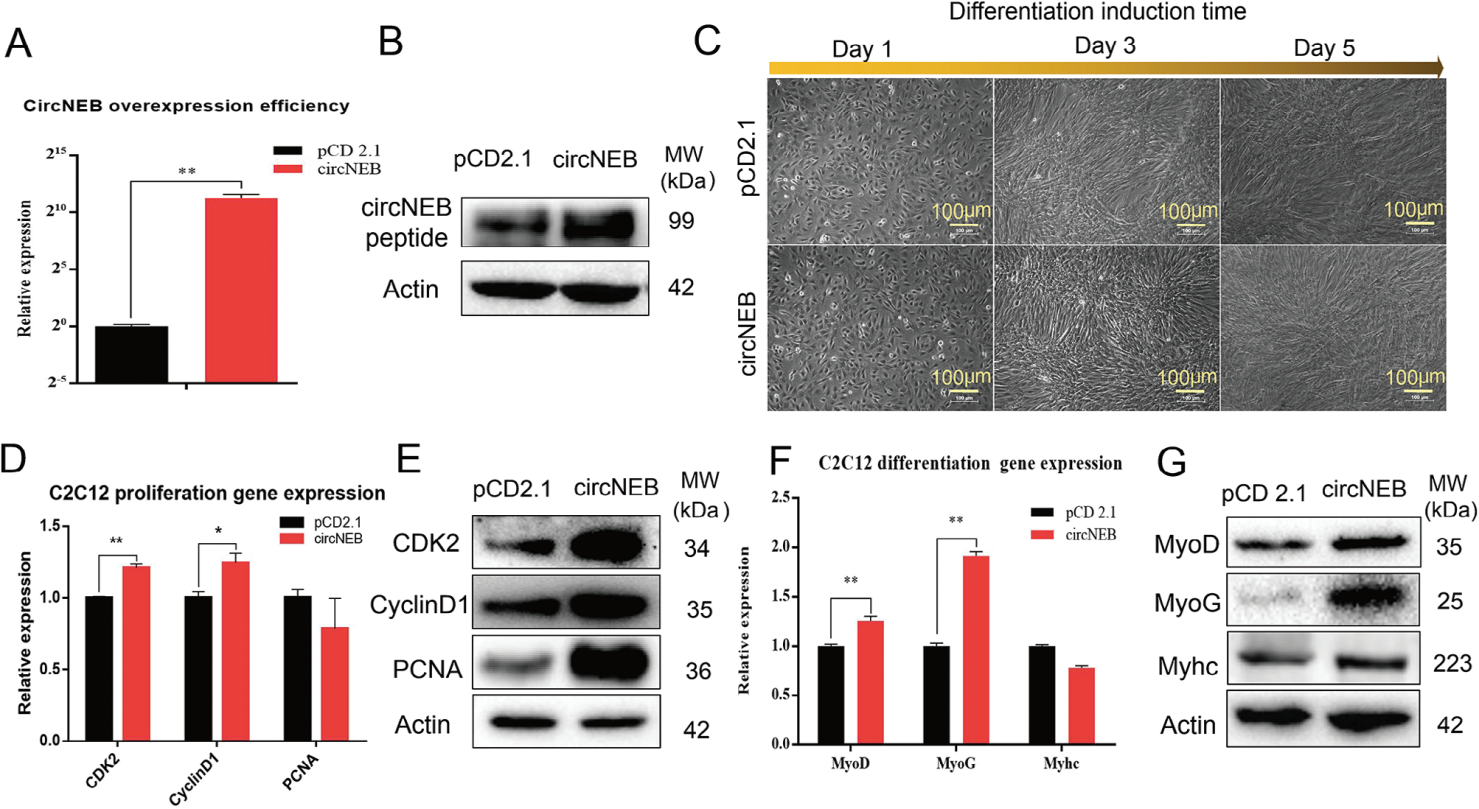
CircNEB promotes C2C12 cell proliferation and differentiation. A) Detection of overexpression efficiency of mus‐circNEB in C2C12 cells. CircNEB expression was significantly upregulated after transfection (*n* = 3). B) The expression of mus‐circNEB peptide was detected by western blotting. Overexpression of mus‐circNEB increased the expression of circNEB peptide. C) C2C12 cells were induced to differentiate for 1, 3, and 5 days of myotube differentiation after plasmid transfection (*n* = 3). D) The expression of proliferation‐related genes in C2C12 cells was detected by qPCR. Results showed that the expression of *CDK2* and *CyclinD1* were significantly up‐regulated (*n* = 3). E) The expression of cell proliferation‐related proteins were detected by western blotting, and the expressions of *CDK2*, *CyclinD1*, and *PCNA* were up‐regulated. F) The expression of C2C12 differentiation‐related genes were detected by qPCR assay (*n* = 3). G) Western blotting assay of differentiation related proteins in C2C12 cells. Data are represented as the mean ± SEM and analyzed by Student's *t*‐test. ^*^, ^**^ represent *p* < 0.05, < 0.01, respectively. *p*‐values < 0.05 were considered statistically significant.

By comparing the sequence of mouse *NEB* gene, we found that the mRNA sequences homology between mouse and cattle were 88%. Therefore, we tried to clone mouse mus‐circNEB, which is 86 bp different from bos‐circNEB (Figure S5, Supporting Information). Subsequently, we overexpressed mus‐circNEB in C2C12 and induced differentiation (**Figure** **4**A–C). With the overexpression of mus‐circNEB, its coding polypeptide was increased, indicating that mus‐circNEB can also encode peptide (Figure 4B). Similar to cattle myoblasts, we detected the expression of proliferation and differentiation related genes (Figure 4D–G). The results showed that mus‐circNEB also promoted the proliferation and differentiation of C2C12 myocytes.

#### 2.2.4 Transcriptome Analysis of Gene Expression Regulation of bos‐circNEB on Proliferation and Differentiation

To investigate the regulatory role of bos‐circNEB in myoblasts, we performed transcriptome analysis on myoblasts overexpressing bos‐circNEB during the proliferative and differentiation phases.

In the proliferative phase, we observed 351 significantly up‐regulated genes and 218 significantly down‐regulated genes (Figure S6A, Supporting Information). Gene Ontology (GO) analysis revealed that circNEB‐regulated genes were involved in various biological processes, including negative regulation of BMP signaling pathway, microspike assembly, and cellular component movement. These genes were distributed in the nucleus and cytoplasm. At the molecular level, the functions of these genes included calcium ion binding, titin Z domain binding, and protein binding (Figure S6B, Supporting Information). KEGG analysis demonstrated enrichment in cellular processes such as cell cycle, regulation of actin cytoskeleton, and focal adhesion. Significantly enriched signaling pathways included MAPK, PI3K‐Akt, and calcium signaling pathways. Additionally, ubiquitination and ribosomal processes were also enriched (Figure S6C, Supporting Information).

During myoblast differentiation, circNEB regulated 114 genes, with 50 genes being significantly up‐regulated and 64 genes being repressed (Figure S7A, Supporting Information). GO analysis revealed enrichment in biological processes such as RNA‐dependent DNA biosynthesis, cardiomyocyte communication, and spermine catabolic. At the cellular level, the differential genes were involved in protein complexes and myosin complexes formation. In terms of molecular functions, they were associated with cardiomyocyte communication, RNA‐directed DNA polymerase activity, and actin filament binding (Figure S7B, Supporting Information). KEGG analysis demonstrated enrichment in signaling pathways including AMPK, FoxO, and PI3K‐Akt during the differentiation of myoblast (Figure S7C, Supporting Information). To validate our findings, we performed qPCR assay on the transcriptional expression of PI3CA, a core gene of the PI3K‐Akt pathway, and its downstream Akt1 gene. The results showed significant up‐regulation consistent with the transcriptome analysis (Figure S7D, Supporting Information).

#### 2.2.5 Co‐Inmunoprecipitation (Co‐IP) Reveals the Interaction Proteins of bos‐circNEB

**Figure 5:**
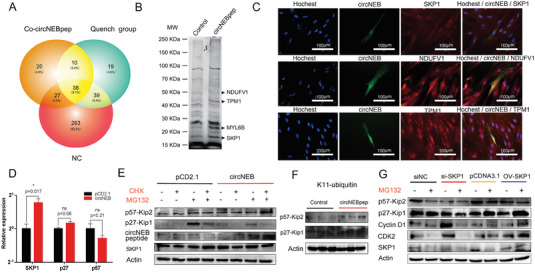
Co‐IP analysis of the circNEB peptide interacting proteins, cell fluorescence co‐localization and detection of myocyte protein ubiquitination. A) Mass spectrometry analysis of Co‐IP proteins, bos‐circNEB peptide specifically pulled down 20 proteins. The quenched group was the negative control and the NC group was the blank control. B) Co‐IP pulled down proteins were separated by SDS‐PAGE and revealed differential proteins at 19, 23, 43, and 51 kDa that were identified by mass spectrometry as SKP1, MYL6B, TPM1, and NDUFV1. C) In the figure, green is the fusion expression of EGFP‐circNEB peptide, red is the signal of Cy3 secondary antibody binding, and blue is the nucleus stained by hoechest. The co‐localization of SKP1, TPM1 and NDUFV1 proteins with EGFP‐circNEB peptide were detected. D) qPCR detected SKP1, p27, and p57 expression (*n* = 3). E) The protein expression of p27, p57, and SKP1 was assessed by Western blotting. Bos‐circNEB was transfected into mature myocytes and empty pCD2.1 vector was transfected into the control group. CHX and MG132 were added to the medium 24 h after transfection, with “‐” indicating no addition and “+” indicating the addition of additives. F) K11 ubiquitination modified western blotting detection. p27‐Kip1 (27 kDa) and p57‐Kip2 (57 kDa) bands were detected, respectively. G) The protein expression of p27, p57, cyclinD1, and CDK2 was assessed by Western blotting. SKP1 protein expression was manipulated in mature myocytes using siRNA and overexpression techniques, with si‐NC and pCDNA3.1 serving as control groups. MG132 was added to the medium 24 h after transfection, with “‐” indicating no addition and “+” indicating the addition of additives. Data are represented as the mean ± SEM and analyzed by Student's *t*‐test. ns, ^*^ represent *p* > 0.05, < 0.05, respectively. *p*‐values < 0.05 were considered statistically significant.

Although the transcriptome was enriched for many regulated genes, the target proteins directly bound by the circNEB peptide were not clear. In order to explore the circNEB peptide interactional protein, we pulled down the circNEB peptide and its binding protein by Co‐IP and then analyzed it by protein mass spectrometry. Further, we screened four proteins that bind and were regulated by circNEB peptides (**Figure** **5**A,B). Among them, SKP1 and NDUFV1 proteins are related to muscle cell proliferation, while TPM1 and MYL6B proteins are associated with myofiber maturation. In the proliferative phase of myoblasts, the circNEB peptide fusion protein with EGFP showed the same localization as with SKP1, NDUFV1 and TPM1 (Figure 5C). Green fluorescent signals corresponding to protein fusions with circNEB peptide were detected in the nucleus and cytoplasm, implying that circNEB peptide may be involved in regulatory processes such as transcription and translation. SKP1 was mainly localized in the nucleus with a small amount distributed in the cytoplasm, and the signal distributed in the nucleus coincided with the green fluorescence of circNEB peptide, showing co‐localization within the nucleus. NDUFV1, TPM1 signals co‐localized with the green fluorescence of circNEB peptide in the cytoplasm, implying that it regulated the proliferation and differentiation of myoblasts through mutual interaction in the cytoplasm.

Among these interacting proteins, SKP1 serves as a subunit of ubiquitinase and has been shown to regulate cell cycle processes. We validated the interaction between SKP1 protein and circNEB‐encoded proteins using a SKP1 antibody co‐IP assay. The corresponding spectrum of the bound circNEB junction sequence‐encoded peptide is depicted in Figure S8 (Supporting Information). Therefore, SKP1 protein and circNEB encoded protein can bind to each other and have the same cellular localization, indicating that circNEB encoded protein potentially regulates myoblast ubiquitination and affects cell proliferation.

#### 2.2.6 CircNEB Protein Regulates Myoblast Ubiquitination

The results obtained from the aforementioned experiments suggest that bos‐circNEB protein promotes myoblast proliferation and potentially mediates ubiquitination modification via SKP1. To elucidate the underlying mechanism through which circNEB protein enhances myoblast function, we performed Western blot analysis to examine the overall cellular level of ubiquitination protein modification. The results revealed that circNEB increased the overall ubiquitination level in myoblast (Figure S9, Supporting Information). Subsequently, specific antibodies were used to detect three types of ubiquitination modifications, namely K63, K48, and K11. Our findings demonstrated no significant difference in K63 ubiquitinated protein modification, while K48 and K11 ubiquitination were significantly increased following circNEB‐peptide overexpression (Figure S9, Supporting Information). K48 represents a polyubiquitinated modification that influences complex physiological processes, while K11 primarily regulates the cell cycle.

Previous results showed that circNEB increased the proportion of proliferating myoblasts in the S phase and upregulated the expression of the SCF (Skp1‐Cul1‐F‐box) ubiquitinating enzyme. Upon overexpression of circNEB, we utilized CHX to block neoprotein synthesis and MG132 to inhibit the ubiquitination degradation pathway. The results revealed a significant decrease in p27‐Kip1 expression and no significant difference in p57‐Kip2 expression (Figure 5E). Given that proteins downstream of K11 ubiquitination modification, such as p27‐Kip1 and p57‐Kip2, act as negative regulators of the cell cycle and that circNEB increased K11 ubiquitination modification of p27‐Kip1 and p57‐Kip2 (Figure 5F), it raises the possibility that SCF ubiquitinating enzymes modulate the ubiquitination of p27‐Kip1 and p57‐Kip2. To further explore this question, we manipulated the expression of SKP1, a key subunit of the SCF ubiquitinating enzyme, and added the ubiquitinating enzyme inhibitor MG132. Subsequently, we examined the changes in the expression patterns of cell cycle inhibitors and downstream proliferative genes associated with p27‐Kip1 and p57‐Kip2. The results are presented in Figure 5G. Up‐regulation of SKP1 led to a down‐regulation in p27‐Kip1 and p57‐Kip2 protein expression, along with an upregulation in CDK2, CyclinD1, and PCNA expression. Conversely, interference with SKP1 resulted in the opposite outcome. These findings suggest that SCF ubiquitinating enzymes mediate the degradation of p27‐Kip1 and p57‐Kip2, leading to the accumulation of intracellular proliferative proteins CDK2, CyclinD1, and PCNA, ultimately promoting cell proliferation.

**Figure 6:**
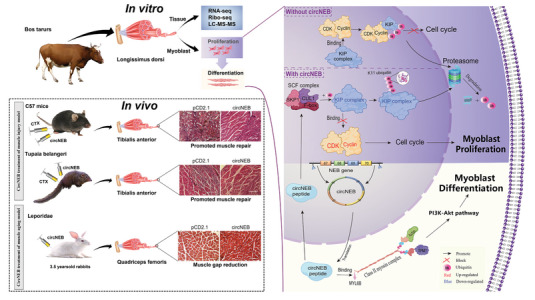
Mechanism of circNEB‐peptide regulating myoblasts. In the figure, circNEB was created by the reverse splicing of exons 67–70 of the *NEB* gene, circularized and transported into cytoplasm for rolling circle translation. The molecular weight of circNEB polypeptide was 99 kDa. The polypeptide was located in the nucleus and cytoplasm. On the one hand, the circNEB polypeptide in the nucleus promotes the formation of SCF ubiquitinase III, and SCF ubiquitinase modifies the ubiquitination of KIP complex (negative regulator of cell cycle) and degrades it through proteasome. The inhibition of KIP complex on CDK and cyclin was relieved, and then the proliferation of myoblasts was promoted. On the other hand, circNEB polypeptides localized in the cytoplasm was targeted to TPM1 in the myosin class 2 complex, which is an important structure of myofilaments and promotes myoblasts differentiation to functional muscle structures. In addition, the downstream PI3K‐Akt signal pathway was activated. The expression of PI3CA and Akt1 transcription was significantly up‐regulated, which jointly regulated the myogenic differentiation of myoblasts.

Based on the aforementioned findings and a series of mechanistic validation experiments, we elucidated the regulatory mechanism of circNEB‐peptide in myoblasts (**Figure** **6**). Specifically, we observed that circNEB‐peptide facilitated the formation of SCF ubiquitinating enzyme in myoblasts. Through K11 ubiquitination modification, circNEB‐peptide facilitated the degradation of p27‐Kip1 within the KIP complex. Consequently, the expression of p27‐Kip1 protein was down‐regulated, thereby relieving its inhibitory effect on CDK2 and cyclinD1, two key proteins involved in cell proliferation, and subsequently promoting cell proliferation. During myoblast differentiation, circNEB‐peptide interacted with the myosin complex II and upregulated the PI3K‐AKT signaling pathway, thus promoting myoblast differentiation. This intracellular regulatory mechanism of circNEB‐peptide unveils distinct pathways governing proliferation and differentiation processes.

### 2.3 Effects of circNEB Peptide on Injured and Aged Muscles In Vivo

#### 2.3.1 Over‐Expression of circNEB In Vivo Promotes the Repair of Muscle Injury

**Figure 7:**
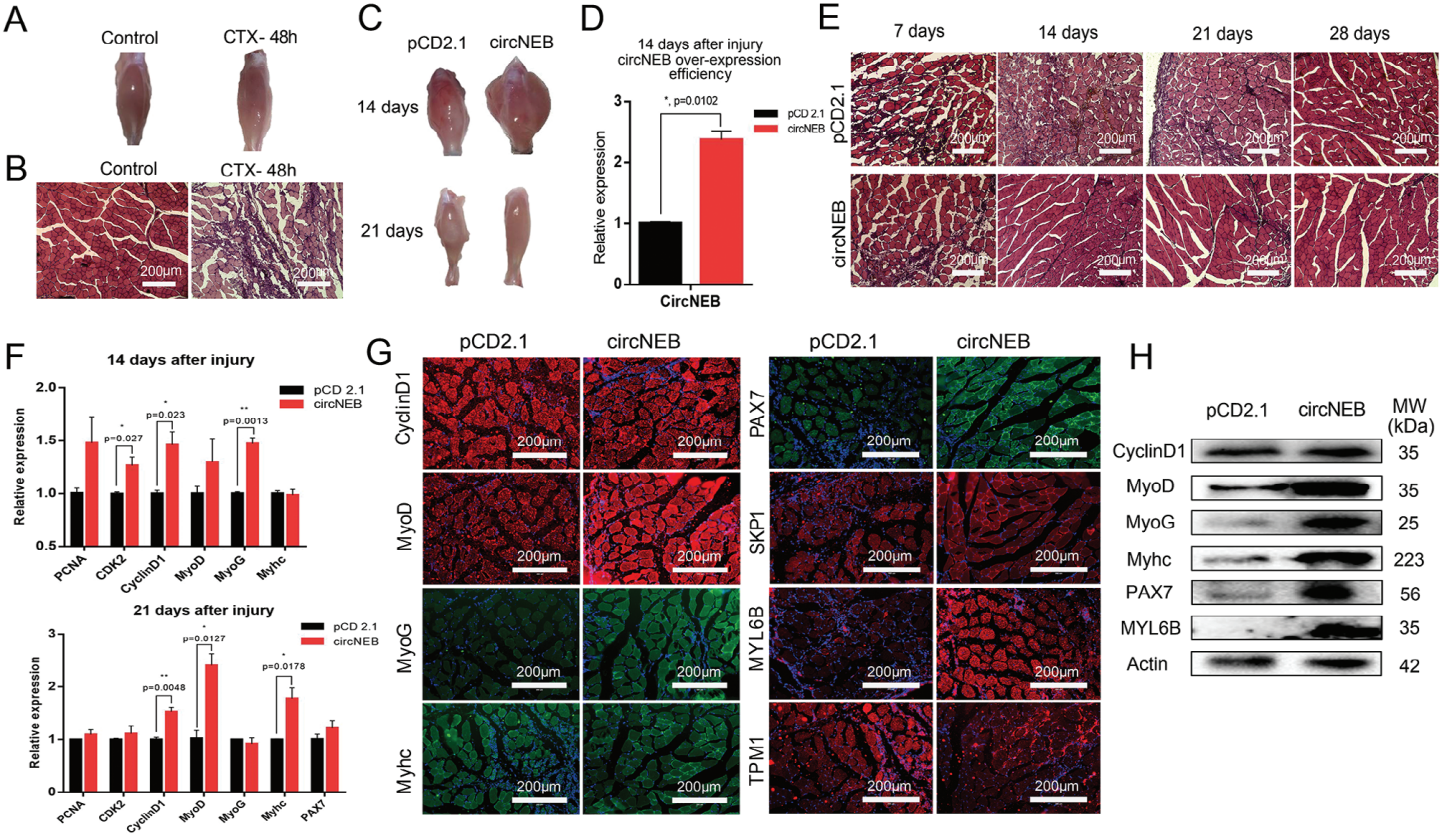
CircNEB promotes regeneration after mice tibialis anterior muscle injury. A) *CTX* were injected into the tibialis anterior muscle of mice to construct the muscle injury model. After 48 h of *CTX* injection, white deposits between muscles were visible. B) HE staining after sectioning showed that muscle fibers dissolved 48 h after *CTX* injection, accompanied by inflammatory cell infiltration. C) After *CTX* muscle injury, mus‐circNEB was overexpressed at the injury site. Staining of tibialis anterior muscle sections at 14th and 21st days, we could observe that the recovery phenomenon was evident in the circNEB group, while muscle damage were still visible in the pCD2.1 control group. D) The efficiency of mus‐circNEB overexpression in vivo was detected by qPCR (*n* = 5). E) HE staining were performed with analysis of muscle tissue sections, and muscle fiber recovery was continuously observed at 7, 14, 21, and 28 days. The circNEB group recovered faster than the control group, and the recovery effect was evident at 14 days. F) qPCR was performed to analyze the relative expression pattern of genes in tibialis anterior muscle 14 and 21 days after overexpression of mus‐circNEB, respectively (*n* = 5). The internal reference gene was actin and normalized with the gene expression of pCD2.1 control group as the reference. G) Muscle tissues were cryosectioned and analyzed by immunofluorescence. H) Western blotting analysis of differentially expressed proteins for 14 days after muscle injury repair (*n* = 3). Data are represented as the mean ± SEM and analyzed by Student's *t*‐test. ^*^, ^**^ represent *p* < 0.05, < 0.01, respectively. *p*‐values < 0.05 were considered statistically significant.

After muscle injury, myoblasts will proliferate and differentiate in large numbers to promote the repair of injury. In order to study the repair function of circNEB on muscle injury, we first constructed *CTX* anterior tibial muscle injury model. Muscle showed significant myolysis with inflammatory cell infiltration 48 h after *CTX* injection (**Figure** **7**A,B). Subsequently, mus‐circNEB was overexpressed locally at the injury site, and the muscle repair status was continuously observed (Figure 7C–E). From the results of HE staining of muscle tissue sections, we observed that the muscle recovered significantly, and the repair time of muscle in circNEB groups were less than that in control groups. In order to analyze the difference of gene expression at different time points of muscle repair, we selected the most evident 14 and 21 days to detect the expression of proliferation and differentiation genes by qPCR. Compared with the control group, the expressions of *CDK2*, *CyclinD1* and *MyoG* in the 14 days samples of circNEB group were significantly up‐regulated. By 21 days, *CyclinD1*, *MyoD* and *Myhc* were significantly upregulated (Figure 7F). This suggests that circNEB promotes the expression of muscle proliferation and differentiation related genes and plays a function in different stages of repair. After that, we examined the protein expression levels in muscles overexpressing circNEB for 14 days by immunofluorescence and western blotting (Figure 7G,H). Findings were consistent with the qPCR results. In addition, we performed muscle injury assays in tree shrews and showed that circNEB‐peptide promoted their muscle regeneration (Figure S10, Supporting Information). Taking these results together, we observed that circNEB promoted the process of muscle injury repair in favor of muscle regeneration.

#### 2.3.2 Over‐Expression of bos‐circNEB In Vivo Promotes Regeneration of Ageing Skeletal Muscle

**Figure 8:**
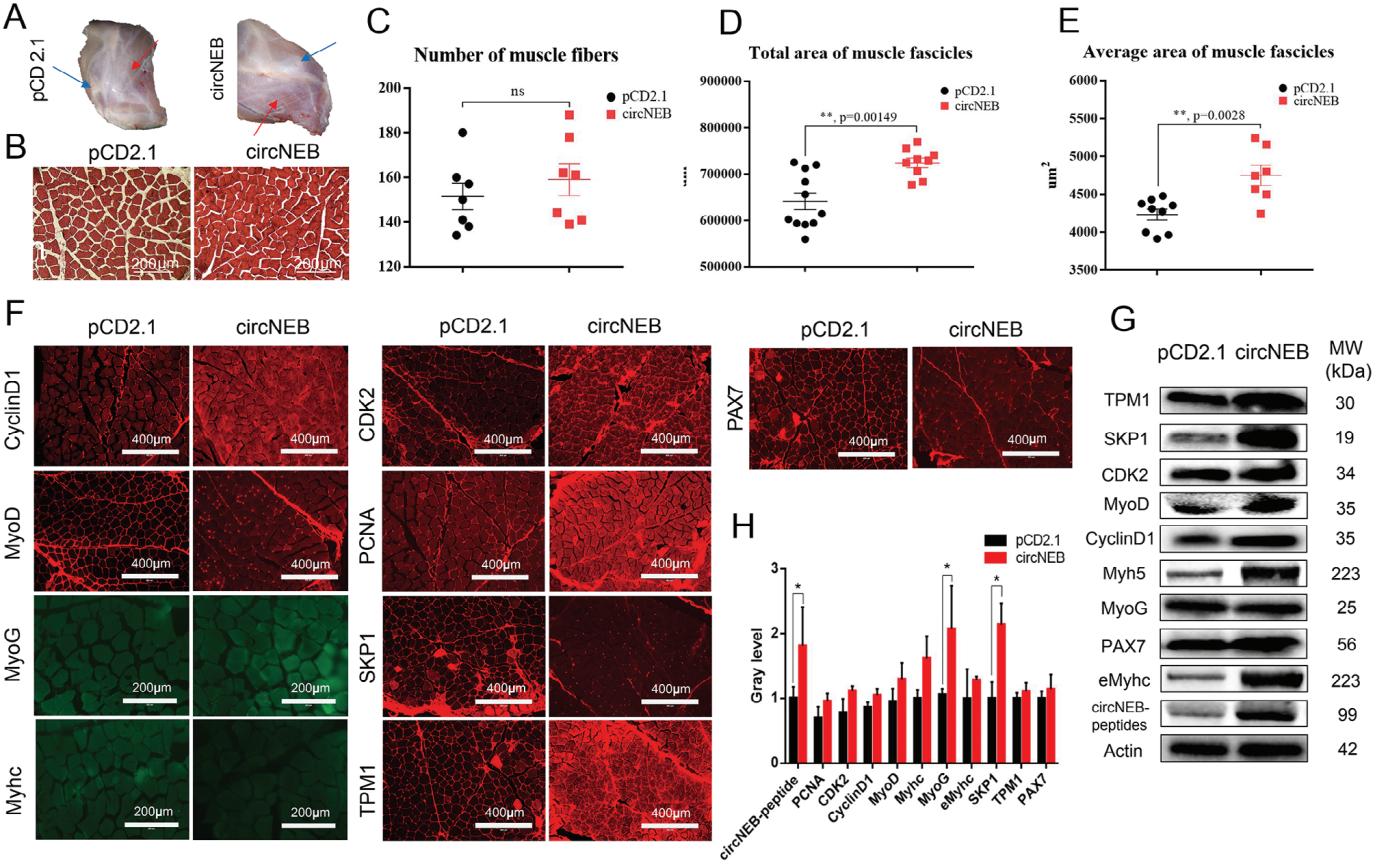
CircNEB improves aging muscle fiber atrophy. A) Overexpression of pCD2.1 and bos‐circNEB in the left and right hind legs of 3.5‐year‐old rabbits, respectively. The blue arrow in the figure is the site of the biceps femoris injection, and the red arrow is the site of the quadriceps femoris injection. Two injections were made at multiple sites per site. B) Muscle fiber cross‐sections were observed by HE staining of muscles from the two groups at 10 days after injection, respectively. C) There was no significant difference in the number of muscle fibers per unit area between the two groups (*n* = 7). D) The total area of muscle fibers per unit area in circNEB group (*n* = 9) was significantly higher than that in control group (*n* = 11). E) The mean cross‐sectional area of muscle fibers in circNEB group (*n* = 7) was significantly higher than that in control group (*n* = 9). F) Immunofluorescence analysis of in situ expression of muscle tissue proteins. G) The expression of muscle development protein was detected by western blotting. H) Statistical analysis of grayscale values in Western blotting assay (*n* = 3). Data are represented as the mean ± SEM and analyzed by Student's *t*‐test. ns, ^*^, ^**^ represent *p* > 0.05, < 0.05, < 0.01, respectively. *p*‐values < 0.05 were considered statistically significant.

Muscle satellite cells are present in muscle and can rapidly proliferate to form myoblasts after injury and promote muscle regeneration. However, in physiologically ageing muscle, satellite cells do not initiate proliferation, so muscles exhibit age‐dependent atrophy. To explore whether circNEB could improve muscle atrophy induced by ageing, we overexpressed circNEB in the biceps femoris and quadriceps femoris muscles of ageing rabbits (**Figure** **8**A). After overexpression of circNEB, a decrease of muscle fiber gap was observed (Figure 8B). Therefore, we counted the number and cross‐sectional area of muscle fibers in the visual field. Notably, although the number of myofibers was not significantly different between circNEB and control groups (Figure 8C), the total myofiber area and mean myofiber area were significantly increased (Figure 8D,E). Proliferation and differentiation related genes were examined by qPCR, immunofluorescence and western blotting (Figure 8F–H). Results showed that PAX7 protein expression was significantly upregulated, representing muscle satellite cell activation. The amount of PCNA, CDK2 and CyclinD1 protein expression increased, illustrating that circNEB promoted senescent cell proliferation. Increased expression of MyoD and Myhc revealed that myocyte fusion was also promoted. In addition, we also detected the protein interacting with circNEB peptide, and found that the expression of TPM1 and SKP1 protein increased. Therefore, it is suggested that circNEB polypeptide also promotes the proliferation and differentiation of muscle cells in aging and atrophic muscles.

## 3 Discussion

CircRNA is a new type of RNA molecule with different biological functions and pathological significance. Among these various functions, the role as “miRNA sponge” is the most prominent.^[^ ^13^ ^]^ However, the general role of circRNAs remains unclear. Interestingly, artificial circRNAs were shown to be translatable in eukaryotic cells.^[^ ^14^ ^]^ Current evidence also suggests that other types of so‐called “non‐coding RNA” can initiate protein synthesis, raising the question of whether endogenous circRNAs can encode proteins in mammalian cells.^[^ ^15^ ^]^ In this study, combining Ribo‐seq, RNA‐seq and peptidome data, we obtained five circRNAs with coding potential in cattle skeletal muscle. One of the interesting circRNAs with coding potential, bos‐circNEB, was formed by the splicing of exons 67–70 of the *NEB* gene. The identification of the circNEB peptide by mass spectrometry demonstrated the authenticity of circNEB encoding in cattle skeletal muscle. Furthermore, sequence analysis showed that the ORF length of circNEB is 729 bp and the rolling loop is translated into a protein with 907 amino acids.

Skeletal muscle formation begins with the specification of myocyte fate by the myogenic transcription factors *Pax7* ^[^ ^16^ ^]^ and *MyoD*,^[^ ^2b^ ^]^ followed by the expression of numerous genes that establish muscle structure and function. An essential step in this process is the fusion of mononuclear myoblasts to form multinucleated muscle fibers. In this study, we found that circNEB encoded a peptide that promotes myoblasts proliferation and differentiation in vitro and in vivo. The robust skeletal muscle‐specific expression of circNEB‐peptide has the ability to synergistically promote fusion together with SKP1 and TPM1. The SKP1 is a key subunit of the Skp1/Cullin/F‐box—type E3 ubiquitin ligase complexes (SCF).^[^ ^17^ ^]^ KIP complex, as a negative regulator of cell cycle, mediates cell exit from cell cycle and stop proliferation by degrading CDK and cyclin families.^[^ ^18^ ^]^ The ubiquitination of KIP complex mediated by SCF ubiquitinase leads to its degradation in cells.^[^ ^19^ ^]^ The present study proposed the hypothesis that circNEB‐peptide promoted SCF formation, ubiquitinated and degraded the KIP complex, a negative regulator of the cell cycle, to rescue myoblasts from exiting the cell cycle and promote myoblast proliferation. In vitro assays confirmed that circNEB‐peptide promotes the degradation of p27‐Kip1 and p57‐Kip2 (KIP complex) by SCF ubiquitinase III, thereby promoted the expression of CDK2 and CyclinD1, and maintained the rapid proliferation of skeletal muscle cells. TPM1, a circNEB‐peptide interaction protein, highly expressed in muscle tissue, plays a central role, in association with the troponin complex, in the calcium dependent regulation of vertebrate striated muscle contraction.^[^ ^20^ ^]^ Mutations in the TPM1 gene cause hypertrophic cardiomyopathy.^[^ ^21^ ^]^ In this study, co‐IP assay detected that circNEB‐peptide combined with TPM1 protein, which promoted the differentiation of muscle fibers and the expression of *MyoD*, *MyoG*, and *Myhc* muscle differentiation genes.

Likewise, in response to injury, myogenic progenitor cells in adult muscle tissue are activated and fuse to generate new muscle fibers.^[^ ^22^ ^]^ Although many of the initial steps in myoblast fusion are similar to other fusion cell types, the components and molecular basis of myoblast fusion have not been fully defined. CircRNAs are important in regulating gene expression. Therefore, circRNAs have received extensive attention as key roles in various physiological and pathological processes such as skeletal muscle regeneration and ageing.^[^ ^23^ ^]^ In this study, we demonstrated that circNEB peptide repaired muscles injured by *CTX* in mouse and tree shrews by promoting the expression of proliferation and differentiation genes. Meanwhile another in vivo experiment demonstrated that circNEB peptide significantly reduced the muscle fiber gap and increased the average muscle fiber area in ageing rabbits. This suggests that the circENB peptide has an important function in repairing injured skeletal muscle and improving the state of ageing muscle.

This study is limited to cultured cells, muscle regeneration, and animal models of ageing. The research implications of circNEB‐peptide may also need to be studied in larger cohorts. In addition, although the role of circNEB‐peptide in promoting myogenesis is well established, its application in animal husbandry production and clinical application still has a long way to go. However, we provided clear evidence that circRNAs encode functional proteins in vivo. Although circRNAs have been reported to be important regulators in key biological processes, no reports of translatable circRNAs or their products have been reported during ruminant myogenesis. Our findings extend current understanding of circRNAs and suggest that the coding potential of circRNAs is largely underestimated. Specifically, evidences from circNEB‐peptide strongly suggest that noncoding RNAs and their translated proteins may play a role in skeletal muscle development as well as muscle regeneration and ageing.

## 4 Experimental Section

### Sample Collection

*Longissimus dorsi* from adult cattle (24 months old, *n* = 30) of an Longlin cattle were collected from the local slaughterhouse in Nanning. After the muscle collection, they were quick‐frozen in liquid nitrogen and stored for transcriptome, ribosome profiling, protein mass spectrometry analysis and verification experiments. C57/6J SPF grade mice (5 weeks old, *n* = 50) were purchased from the laboratory animal center of Guangxi Medical University and sacrificed by neck amputation at the end of the experiment. New Zealand white rabbits were bred by the college of animal science and technology of Guangxi University and raised until 3.5 years (*n* = 4) to serve as an ageing amyotrophic rabbit model. The rabbits were sacrificed by injecting air into ear vein, and the muscle tissues of quadriceps femoris and biceps femoris were taken. All animal experiments followed the procedures of animal ethics committee of Guangxi University (Grant No. GXU‐2021‐158).

### Transcriptome Library Construction and Bioinformatics Analysis

Cattle *Longissimus dorsi* samples were prepared by freezing and grinding with liquid nitrogen the *Longissimus dorsi* of 30 cattles, and an aliquot of ≈ 1 g was taken from each sample to form a mixed sample. Ribosomal RNA was removed after total RNA extraction by TRIzol reagent (Invitrogen, Shanghai, China). The constructed libraries were sequenced with Illumina HiSeq 4000 platform. In the sequencing data, short reads alignment tool Bowtie2 (version 2.4.5)^[^ ^24^ ^]^ was used for mapping reads to ribosome RNA (rRNA) database. The rRNA removed reads of each sample were then mapped to reference genome by TopHat2 (version 2.1.1).^[^ ^25^ ^]^ Anchor reads that aligned in the reversed orientation (head‐to tail) indicated circRNA splicing and then were subjected to find_circ to identify circRNAs.^[^ ^26^ ^]^ For the analysis of the present study with reference transcriptomes, the reference genome versions of cattle were GCF_ 002263795.1_ ARS‐UCD1.2. Transcriptome analysis were performed as described previously.^[^ ^27^ ^]^

### Non‐Coding RNA Open Reading Frames Prediction

In this study, custom NCBI ORFfinder tool (https://www.ncbi.nlm.nih.gov/orffinder/) searches in sequences was performed whose transcripts were annotated as non‐coding regions, including 5′‐, 3′‐untranslated regions and intergenic regions. ORFs using only the ATG as the start codon and 60–450 bp in length (without stop codon) were extracted. The cORF pipeline script was used to search for circRNAs and ensure that circORFs span the junction sequence of circRNAs.^[^ ^28^ ^]^ Each circRNA sequence was multiplied four times and the longest ORF across the circRNA junction site was selected for each of frames (cORF threshold > 20 amino acids).

### IRES Site Prediction of circRNAs

The NCBI tool IRESfinder was used to identify IRES sequences within circRNA.^[^ ^29^ ^]^ The sliding window method was used to evaluate the score of each circRNA sequence with window size of 174 bp and a step size of 50 bp. Sequences with a score >0.5 were screened, and the regions with the highest scores were considered as potential IRES sequences within circRNA.

### Ribo‐Seq Pretreatment, Library Construction, and Sequencing

Tissues were immediately frozen in liquid nitrogen and ground to powder with a mortar in liquid nitrogen before being dissolved in 400 µL of lysis buffer. An incubation step on ice for 10 min followed. Afterward, samples were centrifuged at 20 000 ***g*** for 10 min at 4°C and supernatant was collected. To prepare ribosome footprints (RFs), 7.5 µL RNase I and 5 µL deoxyribonuclease I were added to 300 µL of lysate and incubated at room temperature on a shaker with gentle mixing for 45 min. Subsequently to ribosome recovery, the liquid was passed through a size exclusion column (illustra MicroSpin S‐400 HR Columns; GE Healthcare; catalog no. 27‐5140‐01) and centrifuged at 600 ***g*** for 4 min at room temperature. 100 µL of digested RFs was added to the column and centrifuged at 600 ***g*** for 2 min. Next, 10 µL of 10% (wt/vol) SDS was added to the eluate, and RFs > 17 bp in size were isolated with the RNA clean and concentrator‐25 Kit (Zymo Research; R1017). RNase H and DNase I were then used to digest the probes and purify the RFs with magnetic beads (vazyme). After recovering ribosome footprints, NEBNext Multiple Small RNA Library Prep Set for Illumina (cat. nos. E7300S, E7300L) was used to create the ribo‐seq library. Finally, sequencing was performed by Illumina 2500 platform (Gene Denovo Biotechnology Co., Guangzhou, China).

### Peptidome Library Construction and Mass Spectrometry

Muscle tissues were ground in protein buffer (500 mm Tris HCl, 50 mm EDTA, 700 mm sucrose, 100 mm KCl, 2% β‐Mercaptoethanol, and 1 mm phenylmethylsulfonyl fluoride, pH 8.0), followed by the addition of an equal volume of Tris‐saturated phenol to extract protein. Individual samples (100 µg of protein) were taken for protein enzymolysis using the FASP method and the post enzymatic peptides were subsequently analyzed by LC‐MS‐MS with Q‐EXACTIVE (Thermo, USA).^[^ ^30^ ^]^ The collected data were analyzed with Proteome Discoverer 2.1.0182 (Thermo, USA). Relevant data processing parameters were set as follows, Search engine: Sequest HT; Protein database: UniProt database and transcriptome prediction ncRNAs coding polypeptide library; Enzyme: trypsin; Miss cleavages: 2; Peptide mass tolerance: ± 10 ppm; Fragment mass tolerance: ± 0.02 Da; Peptide FDR: < 1%; Protein q‐value: < 1%.

### qPCR

RNA extraction from muscle tissue and myocytes was performed as previously described. The total RNA was transformed into cDNA by using Hiscript III reverse transcription Kit (Vazyme, Nanjing, China). qPCR was performed on LightCycler480 II (Roche, Basel, Switzerland) using AceQ qPCR SYBR Green Master Mix (Vazyme, Nanjing, China). The primers used in this study are listed in Table S3 (Supporting Information).

### Cell Cycle Analysis

Cattle myoblasts were cultured in 60 mm dish. 24 h after transfection with pCD2.1‐circNEB plasmid, the cells were digested with trypsin when the cell confluence was ≈ 80%. Ethanol (70%) was added dropwise and the cells were fixed overnight at −20°C. Subsequently for the propidium iodide (PI) staining, cells were washed twice with PBS and incubated in FxCycle PI/Nnase Staining Solution (Thermo, USA) for 30 min in the dark at room temperature. The cell cycle was subsequently analyzed by Attune NxT (Thermo, USA), and the generated data were analyzed by FlowJo 10 software for the proportion of each cell cycle.

### CircNEB Peptide Rabbit Antibody Preparation

Prediction of antigen epitopes of bos‐circNEB encoded peptides by B cells was accomplished on neural network algorithm through online tools (https://webs.iiitd.edu.in/raghava/abcpred/). The antigen prediction parameters were as follows: ABCpred, threshold >0.8; length = 16 aa.

Synthesis of antigenic epitope amino acid sequences located in the N‐terminus with highest scores for the production of bos‐circNEB polypeptide primary antibodies by immunization of rabbits: The immunization procedures were: primary immunization (Freund's adjuvant complete mixed with 10 mg antigen), and three boost immunizations (Freund's adjuvant incomplete mixed with 5 mg antigen). An interval of 10 days separated each immunization. The antibody were determined by protein dot blot hybridization using the method referred to previous studies.^[^ ^31^ ^]^

### Cell Proliferation Assay

The proliferation state of cattle *Longissimus dorsi* cells was investigated with Cell Counting Kit‐8 (CCK‐8) (Vazyme, Nanjing, China) and Cell‐Light EdU Apollo 567 In vitro Imaging Kit (RiboBio, Guangzhou, China). The manufacturer's instructions were followed for both procedures.

### Western Blotting Analysis

Total protein extraction from samples was performed with RIPA buffer containing 1% PMSF (Solarbio, Beijing, China). The protein concentration was detected by BCA Kit (Solarbio, Beijing, China). Subsequently, the total proteins were separated by electrophoresis in 10% SDS‐PAGE gel. The gel protein was transferred to 0.22 µm nitrocellulose membrane (Pall, New York, USA) by Trans‐Blot SD instrument (Bio‐Rad, California, USA). Nitrocellulose membranes were blocked in 5% skimmed milk. Primary antibodies (β‐actin: 66009‐1‐Ig, CyclinD1: WL01435a, PCNA: WL03213, CDK2: 60312‐1‐Ig, MyoD: 18943‐1‐AP, MyoG: 67082‐1‐Ig, Myhc: ab207926, TPM1: 28477‐1‐AP, SKP1: 10990‐2‐AP, SKP1‐IP: 32–3800, PAX7: bs‐2413R) and HRP‐conjugated secondary antibodies (Anti‐Rabbit: SA00001‐2, Anti‐Mouse: SA00001‐1) corresponding to the two species were incubated. Finally, the nitrocellulose membranes were imaged by chemidoc XRS+ system (Bio‐Rad, California, USA) in ECL Plus solution (Solarbio, Beijing, China).

### Immunofluorescence

Cells were grown to 70%–80% confluence, fixation were performed by 4% paraformaldehyde for 15 min at room temperature, followed by PBS washing. The membrane was permeabilized with 0.5% Triton X‐100 in PBS for 20 min at room temperature. The solution was subsequently discarded. Fixed material was washed in PBS three times and blocked with 10% goat serum for 1 h. Dilutions of primary antibodies (TPM1: 28477‐1‐AP, SKP1: 10990‐2‐AP, MyoD: 18943‐1‐AP, MyoG: 67082‐1‐Ig, Myhc: ab207926, CyclinD1: WL01435a, PCNA: WL03213, CDK2: 60312‐1‐Ig, and PAX7: bs‐2413R) were prepared according to the reagent manufacturer's instructions and incubation was performed overnight in a wet box at 4 °C. The next day, the primary antibody solution was discarded and fixed cells were washed with PBST (0.5% Tween 20 PBS solution) three times for 3 min each. Then, the fluorescent secondary antibody (SA00009‐2, SA00003‐1, Proteintech, Hubei, China) was applied with a dilution ratio of 1:200 and all was incubated at room temperature for 1 h. Further three washing steps with PBS were accomplished. Nuclei were stained with DAPI for 5 min at room temperature. Fluorescence images were acquired under a Nikon fluorescence microscope after staining was completed.

### CircRNAs Translation In Vitro

Total RNA from muscle tissue was extracted as described previously. Total RNA samples were digested by RNase R (Geneseed, Guangzhou, China) and linear RNA were broken. The translation potential of circRNA was subsequently detected by rabbit reticulocyte lysate system (Thermo, USA). The manufacturer's instructions were followed. The translation products were subsequently analyzed by western blotting to determine whether the circNEB had translation products. Primary antibody used was rabbit antibody as described previously, and secondary antibody was anti‐Rabbit (Proteintech group, Wuhan, China).

### Cell Culture and Treatment

*Longissimus dorsi* muscle of 4‐month‐old cattle fetus with body length of ≈ 15 cm were used for primary cell isolation and culture. The cell isolation method was consistent with previous studies.^[^ ^27b^ ^]^ In this study, muscle tissue cells were isolated and purified from myofibroblasts through enzymatic digestion followed by 2–3 rounds of differential walling. During each round, the total cell population was incubated for 1 h, after which the cells attached to the wall were discarded, and the non‐attached cells were collected for further incubation. This process was repeated 2–3 times to effectively eliminate myogenic fibroblasts. The purity of the isolated cells was confirmed by inducing their differentiation and detecting the expression of muscle marker genes such as MyoD and MyoG. Pending a near 85% confluence of the cells, myoblasts were induced to fuse and differentiate with 2% horse serum (Gibco, MA, USA). Mature myotubes were visible after 5 days of induced differentiation.

### Overexpression and Knockout of circNEB in Myoblasts

The full‐length cattle and mouse circNEB sequences were cloned by back‐to‐back primers, respectively, and cloning primers are listed in Table S3 (Supporting Information). The cloned fragments were linked to the pCD2.1 vector (Geneseed, Guangzhou, China) through *Kpn* I and *BamH* I restriction enzyme sites. The pCD2.1‐bos‐circNEB and pCD2.1‐mus‐circNEB vectors were obtained. pCD2.1‐bos‐circNEB vector was used to overexpress circNEB in fetal cattle myoblasts. pCD2.1‐mus‐circNEB vector overexpressed circNEB in C2C12 cells. Cas9 knockdown vector was constructed by applying px458 plasmid and ligated by restriction enzyme cleavage site Bbs1.^[^ ^32^ ^]^ The knockdown site was located within 1 kb bases of the intron on both sides of circNEB, and the corresponding sgRNA sequences have been listed in Table S3 (Supporting Information). The overexpression plasmid was transfected into the cells by Exfect Transfection Reagent (Vazyme, Nanjing, China). The transfection method was performed in reference to the instruction manual.

### Overexpression and Interference of SKP1 in Myoblasts

The full‐length cattle SKP1 gene sequence was obtained from NCBI nucleotide database (Accession: NM_001034781.2), and the sequence was synthesized by Sangon Biotech Co. and ligated into pCDNA3.1 vector by enzymatic cleavage site to construct the overexpression bovine SKP1 plasmid. Three interfering fragments were designed and synthesized by Geneseed Co., and the sequences are listed in Table S3 (Supporting Information). Overexpression plasmid and interference fragments were transfected into myoblasts in the same way as in the previous section.

### Statistics of Myotubes and Fusion Cells

Pictures analyzed derived from immunofluorescence staining of myoblasts overexpressing circNEB at the differentiation stage. Counting was performed manually by Image J software, and data were analyzed and plotted by GraphPad software. The number of myotubes in bright field 4x magnification, and the number of myotube fusion cells with fluorescence in 10x magnification were counted in 20 fields, respectively. The differentiation myoblasts transfected with pCD2.1 plasmid was used as the control group.

### Co‐Immunoprecipitation

Total protein was extracted and quantified as described previously. The Co‐Immunoprecipitation step was performed according to Pierce Co‐Immunoprecipitation Kit (Thermo, USA) instructions. In brief, steps are as follows: 1000 ***g*** centrifugation for 1 min to remove the resin preservation solution, and then add 200 µL crosslinked buffer to wash and resuspend the resin. Add 10 µg of rabbit‐derived circNEB peptide antibody, and SKP1 antibody as Co‐IP capture antibody, respectively. Incubated with 3 µL of sodium cyanoborohydride solution for 120 min at room temperature. Then add 200 µL crosslinking buffer into the column, wash twice, and centrifuge to discard the liquid. Add 200 µL quenching buffer and incubate with 3 µL sodium cyanobohydride solution for 15 min. Wash the resin with crosslinking buffer twice and washing buffer six times respectively. The antibody bound resin was incubated with muscle protein lysate at 4 °C overnight. The protein lysate was removed by centrifugation and washed with 200 µL IP lysis buffer for three times. Finally, the resin was eluted twice with 50 µL elution buffer to obtain the captured protein for mass spectrometry identification.

### Muscle Injury Model Construction and circNEB Expression In Vivo

C57/6J mice strain aged 5 weeks (*n* = 50) were used to construct muscle injury model. Cardiotoxin (50 µL) at a concentration of 10 µm was injected into the tibialis anterior muscle of the left leg, and 50 µL of 5% glucose solution was injected into the right leg as a self‐control, respectively. After 24 h of injection, three mice were sacrificed and the anterior tibial muscles of the left and right legs were stripped and fixed with formalin solution for 24 h. Muscle samples were dehydrated successively by 10%, 20%, and 30% sucrose solution. OCT compound (Sakura, USA) was used for embedding tissues. Finally, frozen sections were stained with hematoxylin and eosin (HE) to observe muscle injury. When the model construction was successful, the endotoxin free plasmids pCD2.1‐circNEB and pCD2.1 were respectively transfected into the site of tibialis anterior muscle injury in the left leg by the Entranster‐in vivo (Engreen Biosystem, Beijing, China) transfection reagent. The transfection reagent and plasmid complex were injected once a week for a total of three times.

### Myocyte Ubiquitination Assay

Bovine myoblasts overexpressed and knocked down circNEB, and the SKP1 gene was overexpressed and disrupted in a manner consistent with the methods described previously. 24 h after transfection of the plasmid, cells were replaced with fresh DMEM complete medium and the ubiquitination inhibitor MG132 (SJ‐BP0049A, SparkJade), at a final concentration of 5 µm, and actinomycinone (HY‐12320, MCE), at a final concentration of 100 µg mL^−1^, were added to the medium according to the grouping, respectively. Cell samples were collected after 8 h of incubation to detect ubiquitination levels. Ubiquitin modifications were detected using total ubiquitination antibodies (Ubiquitin Rabbit pAb, A0162, ABclonal), K11 (K11‐linkage Specific Polyubiquitin Rabbit pAb, A18197, ABclonal), K48 (K48‐linkage Specific Ubiquitin Antibody, T55964, Abmart) and K63 (K63‐linkage Specific Ubiquitin Antibody, T56579, Abmart) specific ubiquitinating antibodies were detected by western blotting assay.

### Expression of circNEB in Ageing Muscle

The biceps femoris and quadriceps femoris of 40 months old rabbits (*n* = 4) were chosen as injection sites. In vivo transfection reagent was mixed with pCD2.1‐bos‐circNEB over‐expression plasmid, and pCD2.1 plasmid was used as the control group. Two injections were done with an interval of 4 days. After 10 days, the rabbits were killed after ether anesthesia, and the muscle tissue was dissected and stripped. Fixation and dehydration treatment are the same as described above. The morphological changes of muscle fibers were observed by tissue section and HE staining, and the cross‐sectional area and number of muscle fibers were analyzed by ImageJ software. Finally, the expression levels of genes related to muscle ageing and regeneration were examined.

### Statistical Analysis

Images were counted by Image J (Java 1.8.0_112, 64‐bit) software for cell number and area. The acquired data were applied GraphPad (v6.01) statistical analysis. The data preprocessing method mainly analyzes the data through the normalization method. *t*‐test was used for the analysis between the two groups, whereas variance analysis was performed for multiple groups. *p* < 0.05 was considered as a significant difference, *p* < 0.01 identified as extremely significant difference and marked with ^*^ and ^**^, respectively.

## Conflict of Interest

The authors declare no conflict of interest.

## Author Contributions

K.H., Z.L., and D.Z. contributed equally to this work. H.L., K.H., Z.L., and Q.L. designed and directed the research. K.H., D.Z., X.Y., Y.Y., and K.C. participated in the sampling and preparation of rabbit antibody. K.H. and H.L. analyzed the data and wrote the paper with results from all authors; K.H., D.Z., X.W., T.F., X.L., X.Y., and Y.Y. participated in the production of the chart. K.H., Y.Z., J.H., M.L., X.T., Q.L., D.S., Y.J., A.P., G.H., S.U.R., and H.L. participated in the revision of the article. All authors read and approved the final manuscript.

## Supporting information

Supporting Information

## Data Availability

The data that support the findings of this study are available from the corresponding author upon reasonable request.
