# Supplementary material for: A Circular RNA Generated from Nebulin (NEB) Gene Splicing Promotes Skeletal Muscle Myogenesis in Cattle as Detected by a Multi‐Omics Approach
Source: Adv Sci (Weinh). 2023 Nov 30;11(3):2300702. doi: 10.1002/advs.202300702 (PMC10797441; doi:10.1002/advs.202300702)
Supplement: Supplementary file 1 — Supporting Information [file ADVS-11-2300702-s001.pdf]

ADVANCED SCIENCE
Open Access

Supporting Information

for *Adv. Sci.*, DOI 10.1002/advs.202300702

A Circular RNA Generated from Nebulin (*NEB*) Gene Splicing Promotes Skeletal Muscle Myogenesis in Cattle as Detected by a Multi-Omics Approach

Kongwei Huang, Zhipeng Li, Dandan Zhong, Yufeng Yang, Xiuying Yan, Tong Feng, Xiaobo Wang, Liyin Zhang, Xinyue Shen, Mengjie Chen, Xier Luo, Kuiqing Cui, Jieping Huang, Saif Ur Rehman, Yu Jiang, Deshun Shi, Alfredo Pauciullo, Xiangfang Tang, Qingyou Liu* and Hui Li**

Supplementary Figures

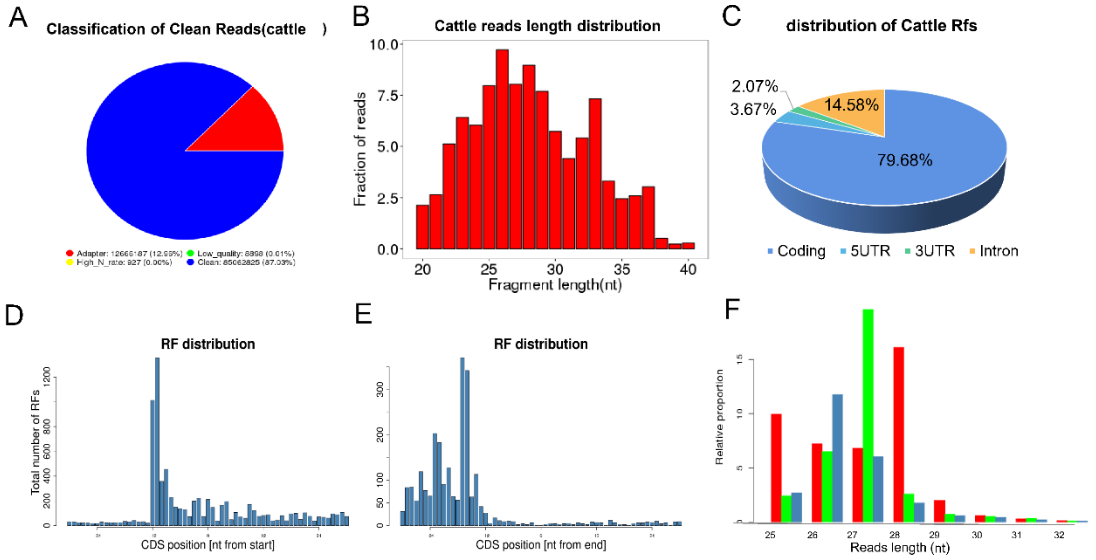

Figure S1. Ribosome binding transcripts of cattle skeletal muscle identified by Ribo-seq. A) Ribo-seq data filtering and quality control. B) Length distribution of ribosome binding sequence. C) Proportion of ribosome binding sequences distributed in different regions of the reference genome. D) Distribution statistics of ribosome binding sequences at the 5’-end of the CDS region. E) Distribution statistics of ribosome binding sequences in the 3’-footprints of the CDS region. F) Ribosome footprints map of codon positions at the 5’-end of a ribosome footprint alignment. In the figure, red, green and blue represent the first, second and third codons of the CDS region, respectively.

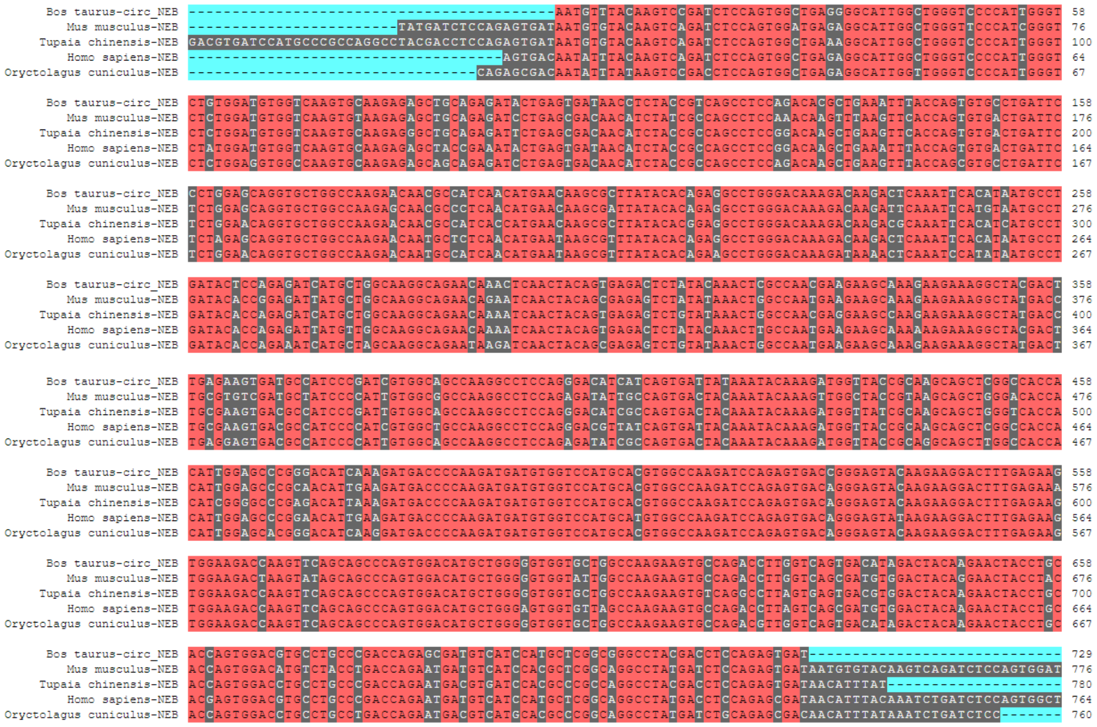

Figure S2. Multiple sequence alignment of bos-circNEB and NEB gene. The exons 67 – 70 of bovine NEB gene sequence was aligned with mouse, tree shrew, human, and rabbit NEB sequences. Bases in red are consensus sequences and bases in gray are imperfect consensus sequences.

This article is protected by copyright. All rights reserved.

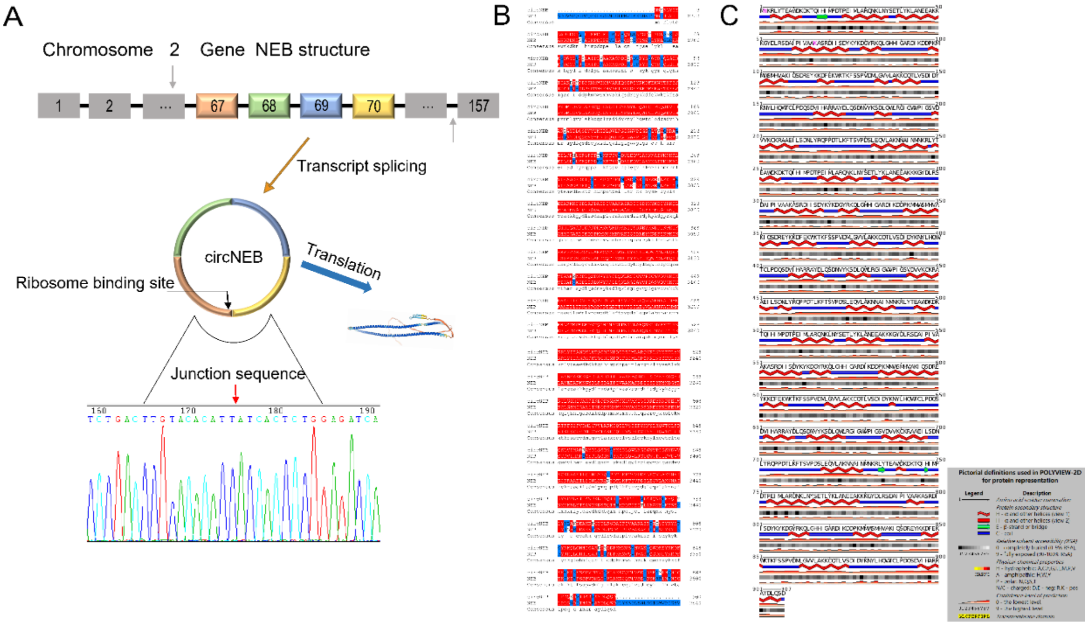

Figure S3. Composition of circNEB and sequence characteristics of translated polypeptides. A) The structure of bos-circNEB in cattle. B) Alignment of bos-circNEB coding peptide and source gene NEB protein sequence homology, the red part is the consistent amino acid sequence. C) The University of Cincinnati College of Medicine's online protein structure Prediction tool (based on sequence prediction) predicts 2D structure maps of bos-circNEB-encoded peptides.

This article is protected by copyright. All rights reserved.

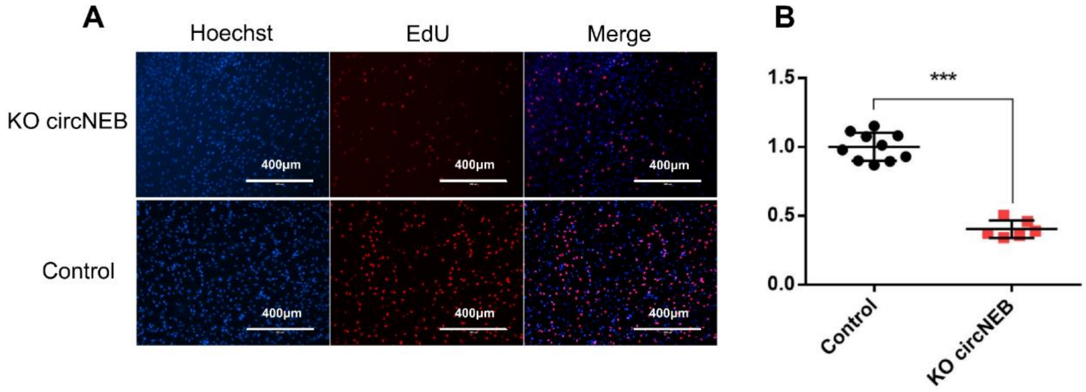

Figure S4. Knockout circNEB-treated myoblast EdU proliferation assay. A) EdU staining plots of cells from bos-circNEB knockout and control groups. B) Data statistics of cell proliferation ratio in control (n = 10) and knockout circNEB groups (n = 6). Data are represented as the mean ± SEM and analyzed by Student's t-test. *** $P < 0.001$ for groups connected by horizontal lines. P-values < 0.05 were considered statistically significant.

This article is protected by copyright. All rights reserved.

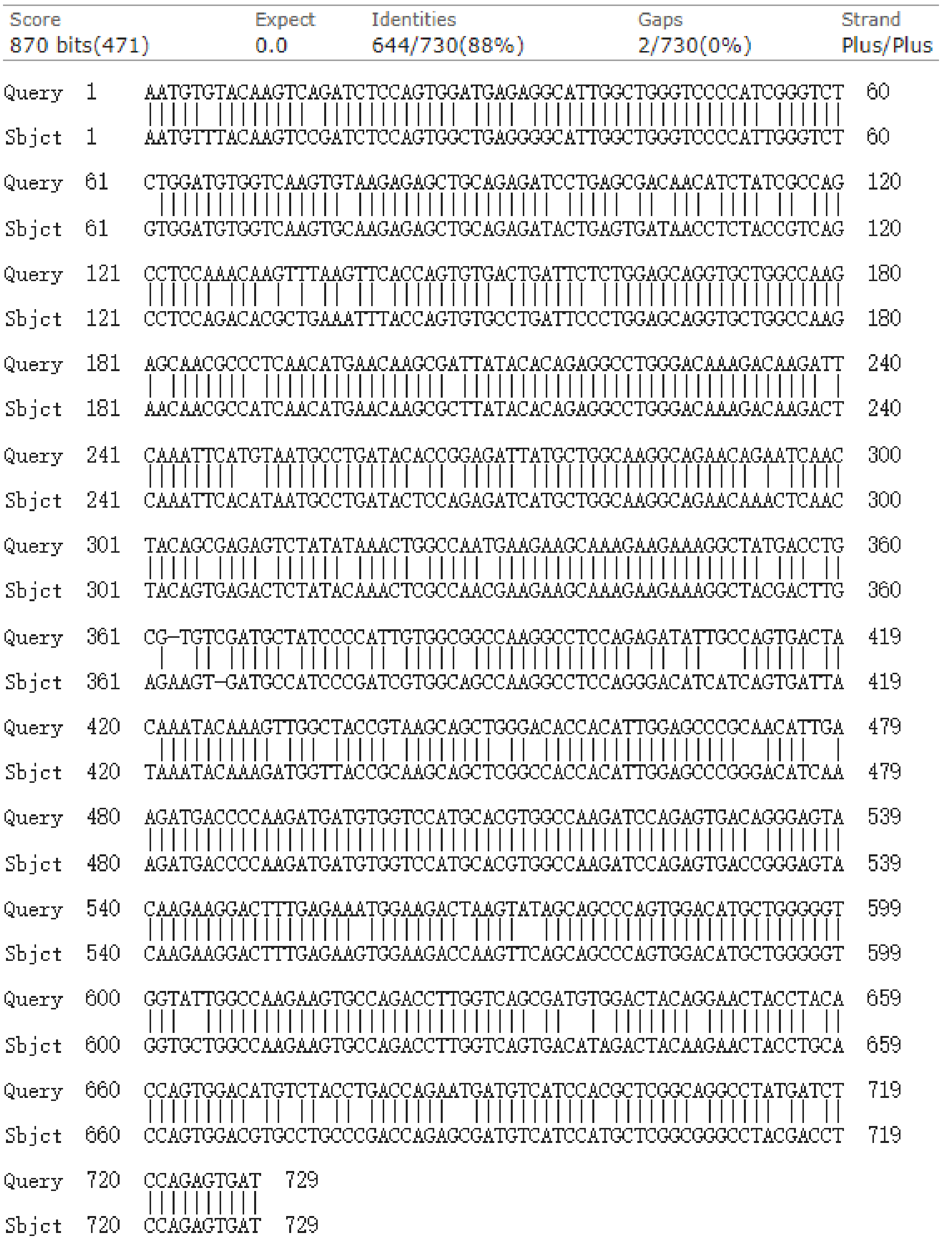

Figure S5. Bos-circNEB and mus-circNEB DNA sequence alignment. NCBI blast tool was used to compare the similarity of bos-circNEB and mus-circNEB sequences. Sbjct sequence in the figure is bovine circNEB sequence, and Query is mouse circNEB sequence. The two sequences were identical in length with 88% similarity and 86 base inconsistencies.

This article is protected by copyright. All rights reserved.

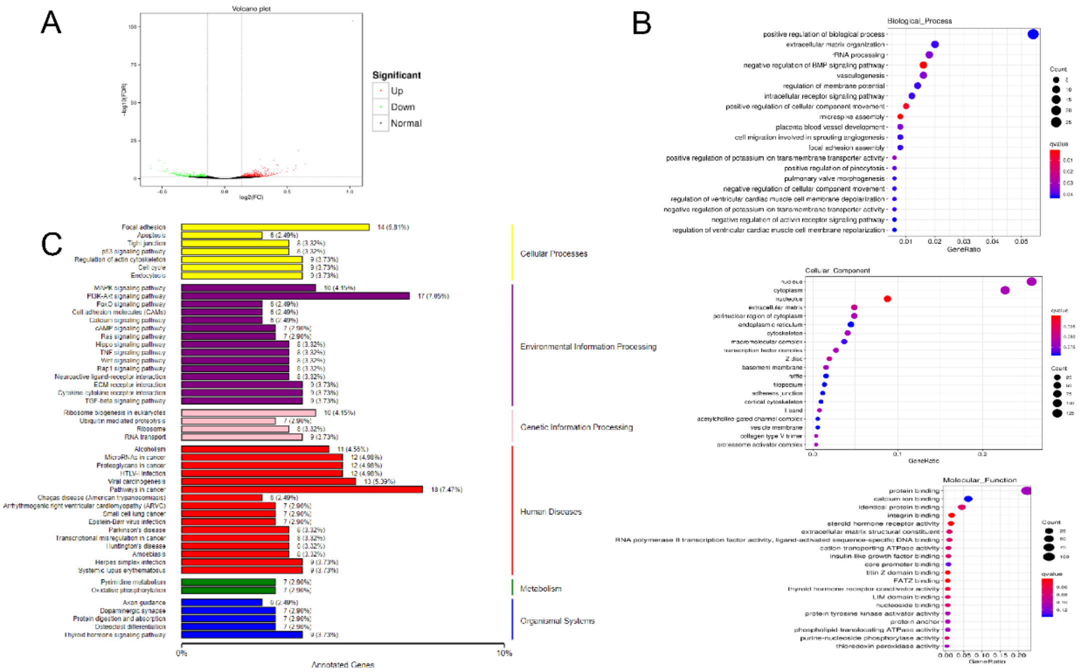

Figure S6. RNA-seq analysis of differentially expressed genes during the proliferative phase of circNEB over-expressed in fetal cattle myoblasts. A) Volcano plot of differentially expressed genes. There were 569 differentially expressed genes during proliferation of myoblasts, of which 351 genes were up-regulated and 218 genes were down-regulated (n = 3). B) GO enrichment analysis of differential genes, including molecular functions, cell components and biological processes. The differential genes were enriched in the nucleolus, proteasome activator complex, positive regulation of cellular component movement, and titin Z domain binding. C) KEGG analysis was used to discover the role of differentially expressed genes during proliferation of fetal cattle myoblasts. The differentially expressed genes were mainly enriched in cell cycle, ubiquitin mediated proteolysis, and regulation of actin cytoskeleton.

This article is protected by copyright. All rights reserved.

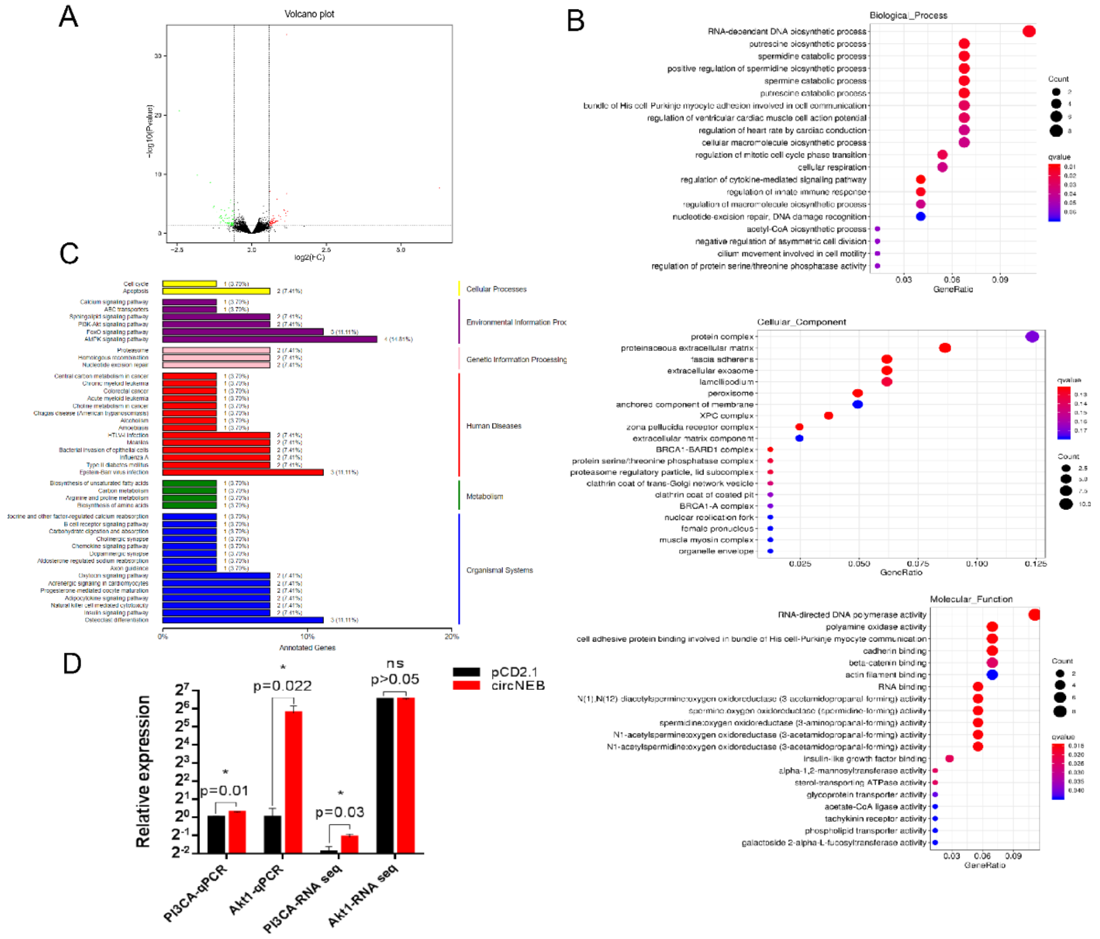

Figure S7. RNA-seq analysis of differentially expressed genes during differentiation from overexpressed circNEB in fetal cattle myoblasts. A) Volcano plot of differentially expressed genes (n = 3). There were 114 differentially expressed genes during differentiation of myoblasts, of which 50 genes were up-regulated and 64 genes were down-regulated. B) GO enrichment analysis of differential genes, including molecular functions, cell components and biological processes. The differential genes were enriched in actin filament binding, RNA-dependent DNA synthesis process, polyamine oxidase activity, etc. C) KEGG analysis was used to discover the role of differentially expressed genes during differentiation in fetal cattle myoblasts. By KEGG enrichment analysis, the differential genes were enriched in AMPK, Foxo, PI3K Akt signaling pathway and so on. D) The differential gene expression of PI3K-Akt signal pathway was verified by qPCR assay (n = 3). Data are represented as the mean ± SEM and analyzed by Student's t-test. ns $p > 0.05$, * $p < 0.05$ for groups connected by horizontal lines. *P*-values < 0.05 were considered statistically significant.

This article is protected by copyright. All rights reserved.

This article is protected by copyright. All rights reserved.

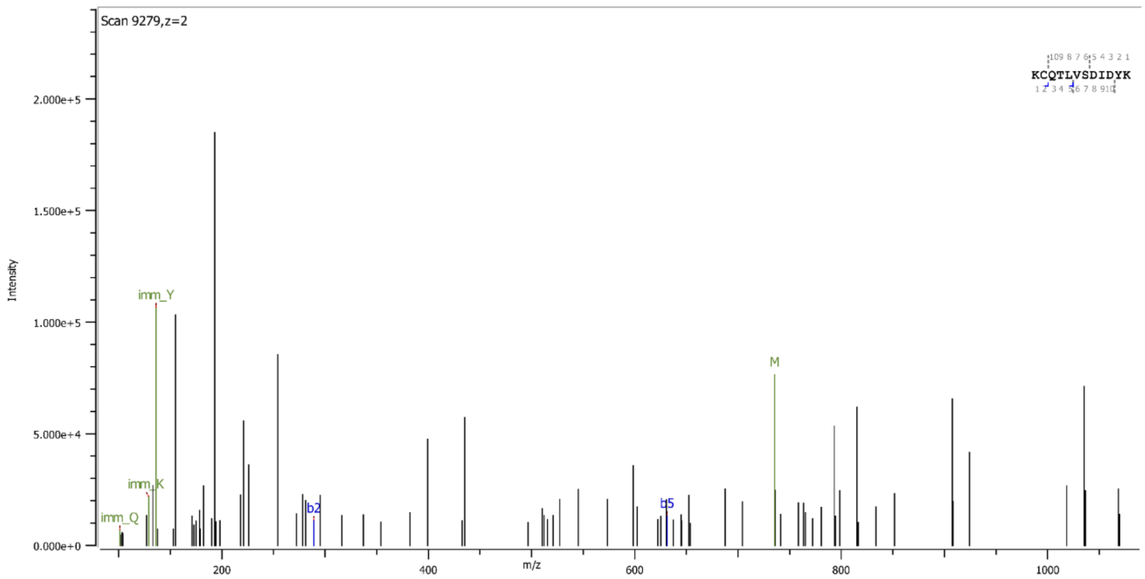

Figure S8. Mass spectral profile of peptides encoded by the ORF junction sequence of circNEB. The profile obtained from co-IP assay, utilizing an antibody against the SKP1 protein, provides characterization of the pulled-down protein. Through mass spectrometry analysis, it mapped to the specific bos-circNEB junction-encoded peptide.

This article is protected by copyright. All rights reserved.

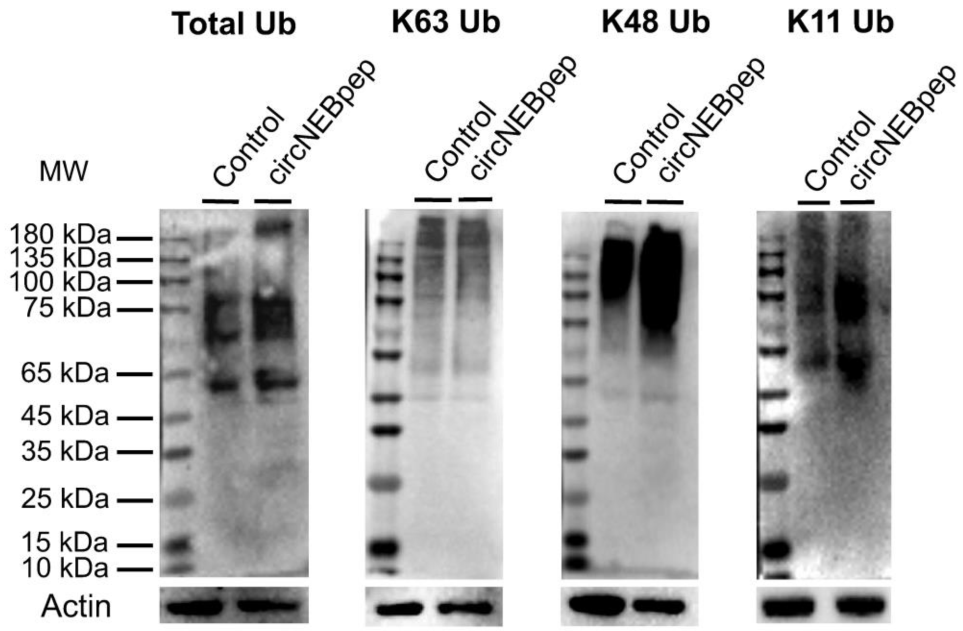

Figure S9. Detection of the effect of circNEB-peptide on the level of ubiquitination in myoblasts. Total ubiquitination (Total Ub), K63, K48 and K11 specific ubiquitination were detected in myoblasts control and overexpression of bos-circNEB, respectively. The control group was transfected with pCD2.1 plasmid, and the circNEBpep group overexpressed bos-circNEB. Actin protein was used as a reference for consistency correction of total protein.

This article is protected by copyright. All rights reserved.

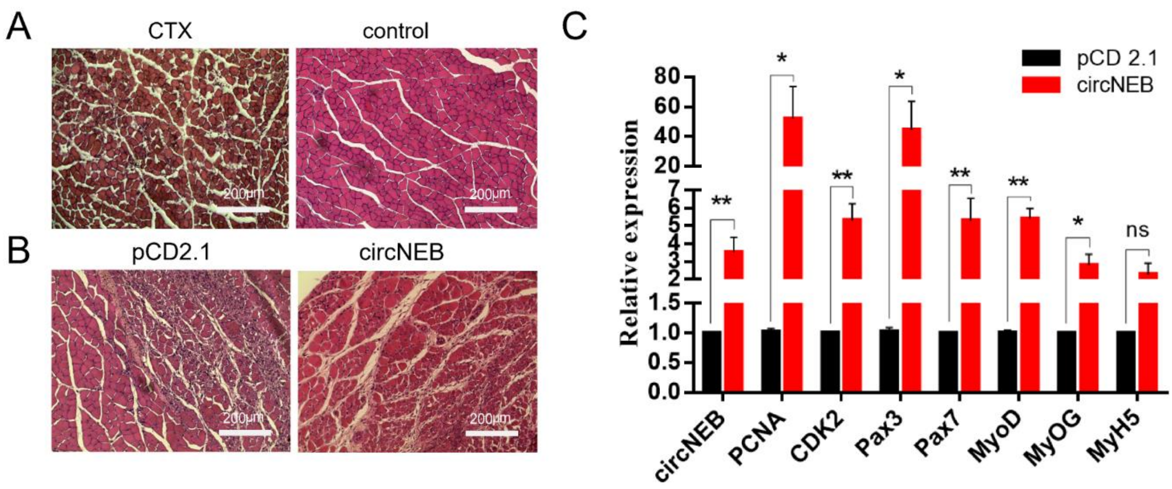

Figure S10. CircNEB-peptide promotes tree shrews muscle injury repair and qPCR detection. A) HE staining of muscle tissues from the tibialis anterior muscle injury model in tree shrews. CTX group was the model of cardiotoxin skeletal muscle injury (48 h after injury), while the control group was at the same site without muscle injury. B) Bos-circNEB was overexpressed locally in the muscle (compared with pCD2.1) to treat the muscle injury model. The length of the scale is 400 μm. C) qPCR was used to detect the gene expression in the damaged muscle of tree shrews (n = 3). Data are represented as the mean ± SEM and analyzed by Student's t-test. ns, *, ** represent $p > 0.05$, < 0.05, < 0.01, respectively. P-values < 0.05 were considered statistically significant.

This article is protected by copyright. All rights reserved.

Table S1

circID	source_gene	IRES_start	IRES_end	Score
novel_circ_000859	NM_001035032.2	244	417	0.732526
novel_circ_000860	XM_024995829.1	25	198	0.822869
novel_circ_000861	NM_001102269.1	1	174	0.839619
novel_circ_000862	NM_001102269.1	51	224	0.919659
novel_circ_000864	XM_024995517.1	301	474	0.774785
novel_circ_000865	XM_024995713.1	851	1024	0.834663
novel_circ_000866	NM_001040573.3	1	174	0.714262
novel_circ_000867	NM_001206123.2	751	924	0.729592
novel_circ_000868	XM_015472641.2	540	713	0.829063
novel_circ_000870	XM_010808145.3	301	474	0.892526
novel_circ_000871	XM_010808145.3	301	474	0.892526
novel_circ_000873	XM_005210398.4	651	824	0.884801
novel_circ_000874	XM_005210398.4	651	824	0.884801
novel_circ_000875	NM_001034597.1	151	324	0.888331
novel_circ_000876	NM_001098905.1	157	330	0.816484
novel_circ_000879	NM_001192821.2	101	274	0.774325
novel_circ_000884	XM_015472675.2	182	355	0.591831
novel_circ_000888	NM_001076387.1	1	174	0.620104
novel_circ_000889	XM_015472673.2	1	174	0.688428
novel_circ_000890	XM_015472673.2	351	524	0.692687
novel_circ_000891	XM_024996310.1	51	224	0.784829
novel_circ_000892	XM_024996320.1	151	324	0.640933
novel_circ_000893	XR_235716.4	343	516	0.565672
novel_circ_000894	XM_024995969.1	301	474	0.805206
novel_circ_000895	XM_024996622.1	1651	1824	0.914452
novel_circ_000896	XM_024996873.1	151	324	0.957521
novel_circ_000899	XM_024996925.1	501	674	0.888945
novel_circ_000900	XM_024996925.1	238	411	0.77518
novel_circ_000902	XR_003036306.1	167	340	0.773339
novel_circ_000903	XR_003036306.1	201	374	0.933965
novel_circ_000904	XM_002690081.6	51	224	0.825299
novel_circ_000906	XM_002690097.6	479	652	0.898126
novel_circ_000908	XM_024996679.1	401	574	0.916232
novel_circ_000909	XR_003036348.1	901	1074	0.951363
novel_circ_000910	XR_003036348.1	901	1074	0.951363
novel_circ_000912	XM_024996589.1	201	374	0.755144
novel_circ_000913	XM_024996522.1	155	328	0.860908
novel_circ_000914	XM_005210800.4	301	474	0.904822
novel_circ_000916	XR_001500979.2	101	274	0.886075
novel_circ_000917	NM_001101174.1	51	224	0.911586
novel_circ_000918	XM_005210804.4	1151	1324	0.682286
novel_circ_000920	XM_024996667.1	201	374	0.826825
novel_circ_000921	XM_024996667.1	51	224	0.795144
novel_circ_000922	XM_024996807.1	251	424	0.941905
novel_circ_000924	XM_005210850.4	1	174	0.826527

novel_circ_000925	XM_005210850.4	1	174	0.826527
novel_circ_000926	XM_005210850.4	451	624	0.899153
novel_circ_000929	XM_010808532.2	51	224	0.712854
novel_circ_000930	XM_024996659.1	151	324	0.781715
novel_circ_000931	NM_001034637.2	140	313	0.751767
novel_circ_000932	XM_024996817.1	1	174	0.839923
novel_circ_000933	XM_002690220.5	1	174	0.698883
novel_circ_000934	XM_002690220.5	1	174	0.810885
novel_circ_000935	XM_002690220.5	101	274	0.659896
novel_circ_000936	XM_024996504.1	101	274	0.694965
novel_circ_000937	XM_010808571.3	201	374	0.674591
novel_circ_000938	NM_001206212.1	351	524	0.906812
novel_circ_000939	XM_024997012.1	201	374	0.939482
novel_circ_000940	XM_024997024.1	414	587	0.843057
novel_circ_000941	XM_024997024.1	51	224	0.822178
novel_circ_000942	XM_024996767.1	301	474	0.883231
novel_circ_000943	XM_005211000.4	15	188	0.952019
novel_circ_000944	XM_010808643.3	201	374	0.85789
novel_circ_000945	XM_024996738.1	301	474	0.692779
novel_circ_000946	XM_024996738.1	1	174	0.603134
novel_circ_000947	XM_005211032.4	1	174	0.664078
novel_circ_000948	XM_005211039.2	101	274	0.662285
novel_circ_000949	NM_001102164.1	151	324	0.844231
novel_circ_000951	XM_002690355.6	101	274	0.883804
novel_circ_000952	XM_002690355.6	651	824	0.913725
novel_circ_000956	XM_024996707.1	51	224	0.774206
novel_circ_000958	XM_024997115.1	601	774	0.969999
novel_circ_000960	XM_002690372.6	301	474	0.884949
novel_circ_000961	XM_024996433.1	151	324	0.738861
novel_circ_000962	XM_024996431.1	51	224	0.726935
novel_circ_000965	XM_015472980.2	251	424	0.657886
novel_circ_000966	NM_205792.2	201	374	0.746505
novel_circ_000968	XM_024997568.1	351	524	0.953348
novel_circ_000969	XM_024997568.1	201	374	0.89371
novel_circ_000970	XM_002690410.5	69	242	0.801843
novel_circ_000972	XM_024997900.1	251	424	0.767287
novel_circ_000973	NM_001075690.1	101	274	0.885474
novel_circ_000974	XM_005211208.4	324	497	0.898385
novel_circ_000975	XM_005211208.4	401	574	0.939124
novel_circ_000976	XM_005211208.4	201	374	0.94292
novel_circ_000978	XM_024997671.1	151	324	0.796292
novel_circ_000979	XM_005211340.3	1	174	0.927706
novel_circ_000985	XM_002690460.5	1	174	0.568089
novel_circ_000986	XM_002690460.5	1	174	0.835772
novel_circ_000987	XM_024997678.1	151	324	0.921888
novel_circ_000988	XM_024997904.1	1351	1524	0.803274
novel_circ_000990	XM_005211352.3	342	515	0.820419

novel_circ_000991	XM_005211324.4	51	224	0.865036
novel_circ_000992	XM_005211324.4	1	174	0.880154
novel_circ_000993	XM_005211278.4	51	224	0.87578
novel_circ_000994	XM_010808862.3	601	774	0.710438
novel_circ_000995	XM_024997927.1	801	974	0.846289
novel_circ_000996	XM_024997927.1	501	674	0.843301
novel_circ_000997	XM_024997935.1	51	224	0.843516
novel_circ_000998	XM_024997935.1	51	224	0.843516
novel_circ_000999	XM_024997935.1	601	774	0.792901
novel_circ_001000	XM_024997937.1	501	674	0.82258
novel_circ_001003	NM_001192392.1	1	174	0.850116
novel_circ_001004	XM_005211486.4	51	224	0.685507
novel_circ_001005	NM_001098091.1	1051	1224	0.834378
novel_circ_001006	XM_024997622.1	301	474	0.831389
novel_circ_001007	XM_024997622.1	451	624	0.912501
novel_circ_001008	XM_015473138.2	101	274	0.920775
novel_circ_001011	XR_003036943.1	151	324	0.610444
novel_circ_001012	XM_010809202.3	112	285	0.827135
novel_circ_001013	XM_010809202.3	101	274	0.859997
novel_circ_001014	NM_001014948.3	51	224	0.50986
novel_circ_001015	XM_024997980.1	551	724	0.887543
novel_circ_001017	NM_001101090.1	883	1056	0.86428
novel_circ_001018	NM_001103282.2	601	774	0.894447
novel_circ_001021	XM_005211684.4	20	193	0.553959
novel_circ_001025	XM_002690847.5	51	224	0.895661
novel_circ_001027	XM_005211719.4	301	474	0.909959
novel_circ_001028	XM_005211717.4	401	574	0.861561
novel_circ_001029	NM_174496.2	251	424	0.871445
novel_circ_001030	XM_010809306.3	1	174	0.891918
novel_circ_001031	XM_002690858.6	177	350	0.82944
novel_circ_001032	XM_003586572.5	1	174	0.832583
novel_circ_001036	XR_003036617.1	701	874	0.879736
novel_circ_001038	NM_001076126.1	751	924	0.847631
novel_circ_001039	XM_024997556.1	1	174	0.716853
novel_circ_001040	XM_024997557.1	401	574	0.673387
novel_circ_001041	XM_005211831.4	1	174	0.698951
novel_circ_001042	XM_024997787.1	1	174	0.52987
novel_circ_001043	XR_003036636.1	601	774	0.901642
novel_circ_001044	XM_024998070.1	1	174	0.913959
novel_circ_001045	XM_024998070.1	1	174	0.775882
novel_circ_001046	XR_236196.4	251	424	0.932546
novel_circ_001049	XR_003036597.1	201	374	0.882071
novel_circ_001050	XM_024997237.1	65	238	0.62729
novel_circ_001051	XM_002691041.4	278	451	0.783635
novel_circ_001053	XM_010809505.3	651	824	0.897808
novel_circ_001054	XM_024998104.1	1151	1324	0.835113
novel_circ_001055	XM_024998104.1	301	474	0.914953

novel_circ_001056	XM_024998104.1	651	824	0.937345
novel_circ_001058	XM_015473301.2	1	174	0.889704
novel_circ_001059	XM_005212175.4	1	174	0.776483
novel_circ_001061	NM_001206687.1	501	674	0.835951
novel_circ_001062	XM_024998170.1	251	424	0.7099
novel_circ_001063	XM_002707712.5	151	324	0.739311
novel_circ_001065	XM_024998334.1	270	443	0.84862
novel_circ_001068	XM_024999718.1	101	274	0.846515
novel_circ_001069	XM_024998705.1	201	374	0.841728
novel_circ_001070	XM_002691185.6	251	424	0.760866
novel_circ_001071	XM_002691185.6	101	274	0.820547
novel_circ_001072	XM_002691185.6	30	203	0.771071
novel_circ_001073	XM_002691185.6	51	224	0.785606
novel_circ_001076	XM_024998650.1	51	224	0.867746
novel_circ_001077	XM_024998650.1	301	474	0.887819
novel_circ_001078	NM_001192476.1	51	224	0.896444
novel_circ_001079	NM_001192476.1	201	374	0.732426
novel_circ_001080	NM_001192476.1	1451	1624	0.853071
novel_circ_001082	NM_001076395.1	264	437	0.75206
novel_circ_001083	NM_001076395.1	1	174	0.730345
novel_circ_001084	NM_001075766.1	1	174	0.521646
novel_circ_001085	XM_024999372.1	301	474	0.851079
novel_circ_001086	XR_003037059.1	301	474	0.746662
novel_circ_001087	XR_003037059.1	51	224	0.876194
novel_circ_001088	XR_003037059.1	851	1024	0.940083
novel_circ_001089	XR_003037059.1	401	574	0.849073
novel_circ_001090	XM_024998488.1	401	574	0.847754
novel_circ_001091	XM_024998488.1	1	174	0.784192
novel_circ_001092	XM_024998401.1	51	224	0.74456
novel_circ_001093	XM_024998401.1	51	224	0.74456
novel_circ_001094	XM_024998401.1	501	674	0.600626
novel_circ_001095	NM_001352124.1	51	224	0.783916
novel_circ_001096	NM_001352124.1	51	224	0.783916
novel_circ_001097	NM_001352124.1	1	174	0.722351
novel_circ_001098	NM_001352124.1	101	274	0.743135
novel_circ_001100	XM_024998429.1	201	374	0.770204
novel_circ_001101	XM_024998352.1	1	174	0.713551
novel_circ_001102	XM_010809914.3	601	774	0.911851
novel_circ_001103	XM_024999054.1	251	424	0.733155
novel_circ_001104	XR_003037050.1	251	424	0.807987
novel_circ_001107	XM_005212593.4	51	224	0.789252
novel_circ_001108	XM_024999073.1	254	427	0.602053
novel_circ_001109	XM_005212572.4	1	174	0.820079
novel_circ_001110	NM_001206209.1	51	224	0.748078
novel_circ_001111	XM_005212622.4	1	174	0.870065
novel_circ_001112	XM_024998816.1	51	224	0.676354
novel_circ_001113	XM_024999408.1	51	224	0.650424

novel_circ_001114	XM_024999405.1	649	822	0.933315
novel_circ_001116	XM_005212778.4	51	224	0.842575
novel_circ_001117	XM_024998869.1	1	174	0.559442
novel_circ_001118	XR_003037202.1	351	524	0.923924
novel_circ_001119	XR_003037202.1	1401	1574	0.929611
novel_circ_001120	XR_003037202.1	51	224	0.863733
novel_circ_001121	XM_024998851.1	1	174	0.741083
novel_circ_001122	NM_174583.2	51	224	0.865289
novel_circ_001123	XM_005212845.4	151	324	0.928463
novel_circ_001124	XM_024999461.1	151	324	0.71349
novel_circ_001125	XM_024998986.1	51	224	0.919205
novel_circ_001127	XM_024998826.1	286	459	0.782097
novel_circ_001128	NM_174335.2	229	402	0.674901
novel_circ_001129	NM_174335.2	301	474	0.633986
novel_circ_001130	NM_174335.2	126	299	0.674901
novel_circ_001133	XM_024998663.1	1451	1624	0.902845
novel_circ_001134	XM_024998664.1	151	324	0.895807
novel_circ_001136	XM_024998664.1	51	224	0.881399
novel_circ_001137	XM_024998664.1	1	174	0.652707
novel_circ_001138	XM_024998664.1	1	174	0.832842
novel_circ_001139	XM_002691475.5	101	274	0.826556
novel_circ_001141	NM_001206156.2	1	174	0.552551
novel_circ_001142	XM_024998328.1	901	1074	0.906995
novel_circ_001143	XM_024999552.1	172	345	0.963263
novel_circ_001144	XM_010810343.2	1	174	0.864363
novel_circ_001145	XM_010810343.2	151	324	0.82787
novel_circ_001146	XM_010810343.2	278	451	0.773742
novel_circ_001147	XM_024998627.1	501	674	0.764519
novel_circ_001148	NM_001081603.2	351	524	0.748749
novel_circ_001149	XM_024999576.1	51	224	0.762254
novel_circ_001150	XM_024999248.1	114	287	0.790347
novel_circ_001151	XM_024998675.1	1	174	0.61284
novel_circ_001152	XM_024998680.1	51	224	0.895491
novel_circ_001153	XM_024998675.1	1	174	0.61284
novel_circ_001154	XM_024999607.1	1	174	0.845319
novel_circ_001155	XM_024999607.1	101	274	0.798392
novel_circ_001159	XM_024998807.1	101	274	0.671825
novel_circ_001160	NM_001103100.1	464	637	0.739157
novel_circ_001161	NM_001103100.1	201	374	0.725871
novel_circ_001162	XM_025000021.1	151	324	0.593828
novel_circ_001163	NM_001098895.2	271	444	0.516477
novel_circ_001164	XM_015473802.2	501	674	0.79459
novel_circ_001165	XM_005213706.4	1001	1174	0.805925
novel_circ_001166	NM_001075493.1	51	224	0.667041
novel_circ_001168	XM_024999836.1	501	674	0.87386
novel_circ_001170	NM_001192773.2	201	374	0.855764
novel_circ_001171	XM_005213721.4	751	924	0.785104

novel_circ_001174	XM_024999833.1	551	724	0.854109
novel_circ_001176	XM_025000215.1	378	551	0.859262
novel_circ_001178	NM_001205616.1	96	269	0.65718
novel_circ_001179	NM_001205616.1	401	574	0.925631
novel_circ_001181	XM_010810724.3	201	374	0.744
novel_circ_001182	XM_025000079.1	1	174	0.767026
novel_circ_001183	NM_001098889.2	1	174	0.786034
novel_circ_001186	XM_025000118.1	21	194	0.686947
novel_circ_001187	NM_001206310.2	283	456	0.71325
novel_circ_001188	NM_001206310.2	151	324	0.661488
novel_circ_001189	XM_005213851.4	801	974	0.749076
novel_circ_001190	XM_005213851.4	2151	2324	0.848813
novel_circ_001191	XM_005213851.4	1651	1824	0.800045
novel_circ_001192	XM_005213851.4	951	1124	0.840492
novel_circ_001193	XM_025000139.1	830	1003	0.743916
novel_circ_001194	XM_010810892.3	1051	1224	0.891032
novel_circ_001195	NM_001205720.1	51	224	0.875777
novel_circ_001196	XM_005213943.3	401	574	0.967788
novel_circ_001198	XM_024999968.1	1	174	0.821847
novel_circ_001200	NM_001206453.2	251	424	0.795321
novel_circ_001201	NM_001206453.2	251	424	0.795321
novel_circ_001202	XM_025000166.1	111	284	0.759216
novel_circ_001203	XM_024999882.1	1	174	0.565833
novel_circ_001204	XM_024999882.1	1	174	0.804386
novel_circ_001206	NM_001098003.1	551	724	0.637708
novel_circ_001207	XM_025000318.1	1	174	0.645249
novel_circ_001208	XM_005214079.2	101	274	0.689871
novel_circ_001209	XR_003037931.1	1	174	0.778837
novel_circ_001210	NM_001080274.2	51	224	0.873822
novel_circ_001211	XM_015473957.2	1	174	0.902913
novel_circ_001212	XM_003586747.4	251	424	0.747498
novel_circ_001213	XM_003586747.4	749	922	0.834481
novel_circ_001214	XM_025000659.1	51	224	0.889586
novel_circ_001216	XM_005214173.4	101	274	0.962728
novel_circ_001217	XM_025000803.1	157	330	0.688753
novel_circ_001218	XM_005214227.4	151	324	0.659865
novel_circ_001219	XM_025000949.1	217	390	0.830304
novel_circ_001220	XM_025000949.1	651	824	0.887795
novel_circ_001223	XM_025000959.1	238	411	0.507033
novel_circ_001226	XR_003037676.1	1	174	0.821096
novel_circ_001227	XM_015473998.2	301	474	0.892079
novel_circ_001228	XM_005214283.4	151	324	0.840079
novel_circ_001229	XM_025000332.1	51	224	0.902182
novel_circ_001231	XM_025001009.1	51	224	0.94313
novel_circ_001233	XM_010811127.3	301	474	0.543036
novel_circ_001240	XM_025000263.1	83	256	0.705754
novel_circ_001243	XM_025000272.1	27	200	0.657656

novel_circ_001245	XM_010811314.3	51	224	0.880494
novel_circ_001246	XM_010811314.3	1	174	0.939604
novel_circ_001247	XM_010811314.3	1	174	0.939604
novel_circ_001248	XM_005214460.4	101	274	0.676063
novel_circ_001250	XM_010811327.2	42	215	0.79251
novel_circ_001251	XM_005214444.4	401	574	0.735214
novel_circ_001252	XM_005214444.4	329	502	0.59555
novel_circ_001253	XM_002692364.6	301	474	0.936086
novel_circ_001254	XM_002692364.6	201	374	0.805577
novel_circ_001255	XM_005214564.4	1	174	0.722822
novel_circ_001256	XM_025001074.1	151	324	0.859158
novel_circ_001257	XR_237007.4	151	324	0.859158
novel_circ_001258	XM_025000260.1	251	424	0.672768
novel_circ_001259	XM_010811460.3	9	182	0.805163
novel_circ_001260	NM_001075669.1	951	1124	0.876803
novel_circ_001261	XM_005214474.3	51	224	0.689719
novel_circ_001262	XM_025001173.1	701	874	0.870968
novel_circ_001264	XM_005214769.4	901	1074	0.589062
novel_circ_001265	XM_002692442.4	101	274	0.968621
novel_circ_001266	XM_025001204.1	51	224	0.865899
novel_circ_001267	NM_001076508.2	51	224	0.640771
novel_circ_001270	NM_001101134.1	101	274	0.785634
novel_circ_001276	XM_025001320.1	51	224	0.829134
novel_circ_001277	XM_025001308.1	101	274	0.91811
novel_circ_001278	XM_025001320.1	101	274	0.821933
novel_circ_001279	XM_025000769.1	801	974	0.893758
novel_circ_001280	XM_005215060.4	201	374	0.845342
novel_circ_001283	XM_025001332.1	101	274	0.818572
novel_circ_001284	XM_025001332.1	51	224	0.814577
novel_circ_001289	NM_205794.1	101	274	0.808785
novel_circ_001295	XM_005215358.4	1	174	0.932059
novel_circ_001297	XM_005215415.4	51	224	0.844988
novel_circ_001298	XM_025001451.1	101	274	0.715214
novel_circ_001301	XM_003586888.5	5	178	0.690253
novel_circ_001302	XM_025001590.1	301	474	0.849496
novel_circ_001303	NM_001192526.1	301	474	0.830258
novel_circ_001304	NM_001192526.1	301	474	0.830258
novel_circ_001305	NM_001192526.1	1	174	0.788082
novel_circ_001306	NM_001192526.1	401	574	0.775319
novel_circ_001307	NM_001192526.1	251	424	0.690594
novel_circ_001308	XM_010812085.3	51	224	0.924411
novel_circ_001309	XM_010812086.3	51	224	0.924411
novel_circ_001310	XM_025001389.1	201	374	0.755205
novel_circ_001311	XM_025001389.1	1	174	0.771731
novel_circ_001312	XM_025001389.1	201	374	0.755205
novel_circ_001313	XM_025001389.1	201	374	0.755205
novel_circ_001314	XM_025001389.1	101	274	0.645606

novel_circ_001317	XR_003038038.1	690	863	0.769538
novel_circ_001318	XM_005215522.4	601	774	0.800426
novel_circ_001319	XM_005215535.4	1	174	0.612209
novel_circ_001320	XM_025001706.1	551	724	0.841336
novel_circ_001321	XM_005215568.4	401	574	0.883071
novel_circ_001322	XM_010812116.3	251	424	0.711389
novel_circ_001323	XM_025001963.1	551	724	0.938759
novel_circ_001324	XM_025001963.1	51	224	0.754945
novel_circ_001325	XM_025001963.1	301	474	0.754945
novel_circ_001326	XM_025001974.1	136	309	0.86661
novel_circ_001327	XM_010812167.3	1	174	0.62887
novel_circ_001328	XM_025001580.1	244	417	0.741136
novel_circ_001329	NM_001102556.1	101	274	0.90303
novel_circ_001330	NM_001102556.1	1001	1174	0.930035
novel_circ_001331	NM_001102556.1	1	174	0.855668
novel_circ_001332	XM_005215688.3	1	174	0.759731
novel_circ_001333	XM_025001459.1	201	374	0.925044
novel_circ_001334	XM_025001459.1	451	624	0.937701
novel_circ_001336	XM_002692846.6	251	424	0.946638
novel_circ_001338	NM_001075423.1	1	174	0.829982
novel_circ_001339	NM_001075837.2	240	413	0.884071
novel_circ_001340	XR_003038031.1	401	574	0.803831
novel_circ_001341	XR_003038031.1	91	264	0.748649
novel_circ_001343	NM_001206791.1	1	174	0.926066
novel_circ_001344	XM_025001757.1	51	224	0.5473
novel_circ_001345	XM_025001757.1	51	224	0.5473
novel_circ_001348	XM_024975290.1	251	424	0.899788
novel_circ_001349	XM_024975404.1	1	174	0.641294
novel_circ_001350	XM_024975300.1	451	624	0.898696
novel_circ_001352	XM_024975300.1	301	474	0.89118
novel_circ_001353	XM_024975300.1	201	374	0.921072
novel_circ_001356	XM_024975217.1	285	458	0.759371
novel_circ_001358	XM_005215850.4	601	774	0.523367
novel_circ_001360	XM_005215850.4	601	774	0.523367
novel_circ_001363	XM_005215841.4	1	174	0.893394
novel_circ_001364	XM_005215841.4	1	174	0.865118
novel_circ_001365	XM_024975501.1	251	424	0.864198
novel_circ_001366	XM_024975295.1	101	274	0.922049
novel_circ_001368	XM_024975378.1	251	424	0.759781
novel_circ_001370	XM_005216057.4	101	274	0.924959
novel_circ_001371	XM_015474678.2	1	174	0.851462
novel_circ_001372	XM_010812454.3	127	300	0.861492
novel_circ_001373	XM_010812454.3	151	324	0.857037
novel_circ_001375	XM_005215917.4	691	864	0.75245
novel_circ_001378	XM_024975797.1	451	624	0.71034
novel_circ_001380	XM_010812538.3	251	424	0.728761
novel_circ_001381	NM_001243290.1	351	524	0.810805

novel_circ_001382	XM_005215931.4	51	224	0.770293
novel_circ_001383	XM_005215931.4	551	724	0.922513
novel_circ_001384	XM_005215931.4	51	224	0.932082
novel_circ_001385	XM_015474712.2	251	424	0.846752
novel_circ_001386	XM_002693428.6	201	374	0.884114
novel_circ_001390	XM_024975871.1	1	174	0.713491
novel_circ_001391	XM_024975871.1	1	174	0.639359
novel_circ_001393	XM_024975047.1	51	224	0.626084
novel_circ_001394	XM_024975246.1	251	424	0.865182
novel_circ_001396	XM_010812757.3	290	463	0.872188
novel_circ_001397	XM_010812730.3	351	524	0.943443
novel_circ_001398	XM_010812730.3	351	524	0.943443
novel_circ_001399	XR_003029362.1	301	474	0.790514
novel_circ_001401	XM_024975434.1	801	974	0.844911
novel_circ_001402	NM_001193007.2	401	574	0.901424
novel_circ_001403	NM_001193007.2	301	474	0.89078
novel_circ_001404	XM_024975273.1	550	723	0.697801
novel_circ_001410	XM_024975266.1	251	424	0.678979
novel_circ_001412	XM_005216463.4	295	468	0.738119
novel_circ_001413	NM_001105630.1	51	224	0.834887
novel_circ_001419	XM_010813095.3	288	461	0.6355
novel_circ_001423	NM_001034289.1	401	574	0.966646
novel_circ_001424	NM_001034338.2	1	174	0.824007
novel_circ_001426	XM_024976698.1	51	224	0.675219
novel_circ_001427	XR_003029612.1	201	374	0.790747
novel_circ_001428	XM_024976087.1	801	974	0.940555
novel_circ_001429	XM_024976087.1	101	274	0.92053
novel_circ_001430	XM_024975966.1	301	474	0.751418
novel_circ_001431	XM_024976270.1	251	424	0.897553
novel_circ_001432	XM_024976270.1	951	1124	0.864408
novel_circ_001434	XM_010813216.2	1	174	0.883682
novel_circ_001436	XM_024976417.1	1	174	0.676673
novel_circ_001437	XM_024976417.1	651	824	0.797817
novel_circ_001438	XM_024976729.1	251	424	0.824531
novel_circ_001440	XM_002694049.5	251	424	0.829876
novel_circ_001441	XM_002694049.5	101	274	0.829281
novel_circ_001443	XM_024976559.1	101	274	0.77182
novel_circ_001444	XM_005217088.4	101	274	0.865548
novel_circ_001445	XM_024976878.1	235	408	0.795189
novel_circ_001446	XM_024976878.1	401	574	0.816338
novel_circ_001451	NM_001205853.2	229	402	0.809471
novel_circ_001452	NM_001205853.2	101	274	0.922897
novel_circ_001454	XM_005217353.4	1801	1974	0.861163
novel_circ_001455	XM_010813575.2	551	724	0.878012
novel_circ_001456	NM_001076044.1	301	474	0.918516
novel_circ_001457	XM_024976438.1	1	174	0.912735
novel_circ_001459	XM_024976900.1	51	224	0.666774

novel_circ_001460	XM_024976892.1	1	174	0.855951
novel_circ_001461	NM_173903.4	1	174	0.574571
novel_circ_001462	XM_024977060.1	151	324	0.789633
novel_circ_001463	XM_024977060.1	151	324	0.921565
novel_circ_001465	XM_024977387.1	1101	1274	0.685453
novel_circ_001466	XM_024977104.1	301	474	0.76956
novel_circ_001467	XM_024977104.1	401	574	0.796594
novel_circ_001468	XM_024977104.1	301	474	0.76956
novel_circ_001469	XM_024977104.1	51	224	0.756777
novel_circ_001470	XM_024977104.1	151	324	0.767165
novel_circ_001471	XM_024977333.1	51	224	0.562097
novel_circ_001472	XM_010813681.3	51	224	0.650623
novel_circ_001473	XM_010813681.3	701	874	0.815746
novel_circ_001474	XM_010813681.3	401	574	0.837281
novel_circ_001475	XM_024977540.1	236	409	0.777074
novel_circ_001476	XM_024977376.1	1551	1724	0.864832
novel_circ_001477	XM_024977157.1	301	474	0.752925
novel_circ_001478	XM_024977157.1	240	413	0.755597
novel_circ_001479	XM_024977157.1	315	488	0.755597
novel_circ_001480	XM_024977157.1	381	554	0.755597
novel_circ_001482	XM_015475366.2	651	824	0.644466
novel_circ_001483	XM_005217509.3	1	174	0.812941
novel_circ_001484	XM_024977406.1	201	374	0.861085
novel_circ_001485	XM_024977406.1	201	374	0.861085
novel_circ_001487	NM_001105416.1	96	269	0.539856
novel_circ_001491	XM_024977417.1	51	224	0.956461
novel_circ_001492	XM_024977417.1	51	224	0.956461
novel_circ_001494	XM_024977580.1	651	824	0.782646
novel_circ_001495	XM_005217547.4	1	174	0.66583
novel_circ_001496	XM_024977586.1	423	596	0.884634
novel_circ_001497	XM_015475394.2	1051	1224	0.885222
novel_circ_001498	XM_005217603.4	3001	3174	0.867416
novel_circ_001499	XM_024977596.1	1	174	0.804933
novel_circ_001500	XM_024977596.1	201	374	0.783471
novel_circ_001501	XM_024977596.1	201	374	0.783471
novel_circ_001506	NM_001101927.2	370	543	0.851694
novel_circ_001507	NM_001101927.2	401	574	0.817943
novel_circ_001509	XM_005217738.4	151	324	0.726179
novel_circ_001510	XM_024977317.1	201	374	0.822005
novel_circ_001511	XM_024977392.1	1	174	0.781738
novel_circ_001512	XM_024977142.1	601	774	0.825165
novel_circ_001513	XR_812339.3	1	174	0.508609
novel_circ_001517	XM_002694505.5	101	274	0.694858
novel_circ_001518	NM_001205475.1	151	324	0.595542
novel_circ_001521	XM_015475528.2	201	374	0.829736
novel_circ_001522	NM_001205333.1	1	174	0.565077
novel_circ_001523	NM_001205333.1	1351	1524	0.732906

novel_circ_001524	NM_001205333.1	151	324	0.543475
novel_circ_001525	NM_001205333.1	151	324	0.541344
novel_circ_001526	NM_001205333.1	51	224	0.674232
novel_circ_001528	XM_002694607.6	151	324	0.530211
novel_circ_001533	XM_010814393.2	351	524	0.626204
novel_circ_001534	NM_001038532.1	87	260	0.696427
novel_circ_001535	XM_024978934.1	401	574	0.905123
novel_circ_001536	XM_024978469.1	701	874	0.586935
novel_circ_001538	XM_005218615.3	212	385	0.694326
novel_circ_001539	XM_024978403.1	51	224	0.954732
novel_circ_001541	NM_001206039.1	1801	1974	0.872109
novel_circ_001542	NM_001206039.1	1115	1288	0.870576
novel_circ_001545	XM_005218707.4	1	174	0.940785
novel_circ_001546	XM_005218707.4	1	174	0.940785
novel_circ_001547	XM_024979003.1	1	174	0.891432
novel_circ_001548	XM_024978188.1	1101	1274	0.839677
novel_circ_001555	XM_024978458.1	2601	2774	0.931807
novel_circ_001561	XM_024979438.1	1320	1493	0.677368
novel_circ_001564	XM_005219885.4	401	574	0.905134
novel_circ_001566	XM_024980618.1	151	324	0.826965
novel_circ_001567	XM_002695642.6	1	174	0.827629
novel_circ_001568	XM_002695618.6	301	474	0.58936
novel_circ_001569	XM_024979607.1	851	1024	0.735692
novel_circ_001570	NM_001205587.2	111	284	0.849724
novel_circ_001571	NM_001122728.1	351	524	0.890958
novel_circ_001572	XM_024980669.1	601	774	0.570664
novel_circ_001573	XM_015458589.2	351	524	0.642319
novel_circ_001578	XM_010815936.2	51	224	0.759162
novel_circ_001579	XM_024979733.1	384	557	0.808747
novel_circ_001581	XM_015458671.2	151	324	0.74273
novel_circ_001588	XM_015458740.2	401	574	0.920215
novel_circ_001590	XR_003030627.1	370	543	0.8677
novel_circ_001591	NM_001101109.1	351	524	0.861737
novel_circ_001595	XM_010816429.3	201	374	0.895302
novel_circ_001596	XM_010816429.3	751	924	0.7724
novel_circ_001597	XM_010816381.3	51	224	0.950207
novel_circ_001599	XM_010816367.3	351	524	0.888834
novel_circ_001601	XM_024980007.1	1451	1624	0.835006
novel_circ_001602	XM_015458932.2	358	531	0.798677
novel_circ_001603	XM_024980968.1	401	574	0.753599
novel_circ_001605	XM_005221050.3	147	320	0.504679
novel_circ_001606	NM_001192189.2	201	374	0.651502
novel_circ_001607	XM_005221185.4	1	174	0.764654
novel_circ_001608	XR_003030599.1	1	174	0.789313
novel_circ_001609	XM_024979892.1	1494	1667	0.758042
novel_circ_001611	XM_002696210.3	1	174	0.643376
novel_circ_001612	XM_024981135.1	951	1124	0.900365

novel_circ_001614	XM_024981420.1	1	174	0.610775
novel_circ_001615	XM_024981423.1	151	324	0.916676
novel_circ_001616	XM_024981423.1	501	674	0.977723
novel_circ_001617	XM_024981423.1	501	674	0.977723
novel_circ_001618	XM_024981423.1	301	474	0.973201
novel_circ_001619	XM_005221433.2	201	374	0.784072
novel_circ_001620	XM_024981302.1	51	224	0.803378
novel_circ_001621	XM_024981302.1	1	174	0.743967
novel_circ_001622	XM_024981407.1	151	324	0.639809
novel_circ_001624	XM_005221436.4	301	474	0.571417
novel_circ_001625	XM_010816742.3	349	522	0.794088
novel_circ_001626	XM_002696322.6	101	274	0.888761
novel_circ_001627	XM_024981405.1	101	274	0.965416
novel_circ_001628	NM_001080329.2	101	274	0.94924
novel_circ_001629	NM_001099012.1	1	174	0.928731
novel_circ_001630	XM_002696335.6	651	824	0.926595
novel_circ_001631	XM_002696335.6	1	174	0.777026
novel_circ_001632	XM_005221521.4	401	574	0.768842
novel_circ_001633	XM_005221530.4	104	277	0.560846
novel_circ_001635	NM_001076070.2	251	424	0.539393
novel_circ_001636	XM_005221557.3	51	224	0.69349
novel_circ_001637	XM_005221557.3	501	674	0.911596
novel_circ_001641	XM_010816811.3	1	174	0.852735
novel_circ_001642	XM_010816811.3	651	824	0.923239
novel_circ_001643	XM_024981350.1	101	274	0.911346
novel_circ_001644	XM_024981350.1	101	274	0.911346
novel_circ_001645	XM_024981350.1	651	824	0.931071
novel_circ_001647	XM_024981491.1	232	405	0.501142
novel_circ_001648	NM_001206584.1	201	374	0.879359
novel_circ_001649	XM_005221585.4	1	174	0.577015
novel_circ_001650	XM_005221585.4	301	474	0.789577
novel_circ_001651	NM_001075948.1	1	174	0.570155
novel_circ_001652	XM_024981525.1	201	374	0.50085
novel_circ_001654	XM_024981527.1	501	674	0.832619
novel_circ_001655	XM_024981532.1	51	224	0.61833
novel_circ_001656	XM_024981538.1	359	532	0.692103
novel_circ_001657	XM_024981538.1	25	198	0.842296
novel_circ_001659	XM_005221700.4	1	174	0.697838
novel_circ_001660	XM_005221700.4	1	174	0.697838
novel_circ_001662	XM_005221718.4	1	174	0.805686
novel_circ_001663	XM_024981831.1	351	524	0.721959
novel_circ_001665	NM_001102181.1	301	474	0.544349
novel_circ_001671	XM_010816948.2	1	174	0.528996
novel_circ_001675	XM_024982325.1	301	474	0.754237
novel_circ_001676	XM_024982325.1	301	474	0.754237
novel_circ_001678	XM_015459183.2	551	724	0.72328
novel_circ_001681	XM_015459209.2	451	624	0.61162

novel_circ_001683	XM_015459194.2	1	174	0.562378
novel_circ_001684	NM_001191456.1	1	174	0.758229
novel_circ_001685	XM_024981947.1	201	374	0.730456
novel_circ_001687	NM_174719.2	451	624	0.766438
novel_circ_001688	XM_005221922.2	469	642	0.869216
novel_circ_001690	NM_001075822.1	251	424	0.829382
novel_circ_001691	XM_015459266.2	451	624	0.657512
novel_circ_001692	XM_005221959.4	101	274	0.771681
novel_circ_001693	XM_024981582.1	201	374	0.835155
novel_circ_001694	XM_005222071.4	801	974	0.930225
novel_circ_001695	XM_010817167.3	51	224	0.907268
novel_circ_001697	XM_024982062.1	1651	1824	0.938431
novel_circ_001698	XM_024982062.1	291	464	0.92053
novel_circ_001699	XM_005222056.4	101	274	0.554927
novel_circ_001700	XM_005222080.4	151	324	0.920969
novel_circ_001702	XM_005222083.4	51	224	0.839418
novel_circ_001703	XM_005222083.4	51	224	0.839418
novel_circ_001705	XM_005222083.4	251	424	0.887094
novel_circ_000002	XM_005201093.4	245	418	0.627607
novel_circ_000003	XM_005201093.4	1151	1324	0.854477
novel_circ_000004	XM_002707686.6	51	224	0.647129
novel_circ_000007	XM_010800940.3	201	374	0.693526
novel_circ_000010	XM_010800945.3	289	462	0.86691
novel_circ_000011	XM_010800945.3	151	324	0.900172
novel_circ_000012	XM_024993874.1	1	174	0.879366
novel_circ_000013	XM_024992141.1	101	274	0.766413
novel_circ_000015	XR_804132.3	1	174	0.644541
novel_circ_000016	XR_804132.3	1	174	0.644541
novel_circ_000017	XR_804132.3	351	524	0.875075
novel_circ_000018	XR_804132.3	401	574	0.93719
novel_circ_000019	XR_804132.3	251	424	0.944732
novel_circ_000022	XM_005201221.4	1	174	0.795595
novel_circ_000024	XM_005201228.4	451	624	0.930568
novel_circ_000026	XM_025001795.1	120	293	0.628278
novel_circ_000027	XM_024989742.1	151	324	0.875489
novel_circ_000029	XM_024994252.1	901	1074	0.918055
novel_circ_000032	NM_001103294.1	301	474	0.856857
novel_circ_000033	XM_010801199.3	1001	1174	0.681366
novel_circ_000034	XM_010801177.3	101	274	0.832373
novel_circ_000035	XM_024990023.1	51	224	0.937771
novel_circ_000038	XM_005201416.4	51	224	0.935041
novel_circ_000039	XM_005201416.4	101	274	0.957229
novel_circ_000040	XM_010801175.3	251	424	0.85118
novel_circ_000041	NM_001193008.1	551	724	0.787699
novel_circ_000042	NM_001193008.1	201	374	0.832723
novel_circ_000043	XM_024994827.1	201	374	0.831388
novel_circ_000044	XM_024994827.1	51	224	0.884579

novel_circ_000045	XM_024994827.1	51	224	0.884579
novel_circ_000046	XM_024991209.1	101	274	0.668441
novel_circ_000048	XM_024994948.1	1	174	0.853211
novel_circ_000049	XM_024994948.1	1	174	0.853211
novel_circ_000050	XM_005201527.4	101	274	0.856527
novel_circ_000053	XM_024984550.1	1	174	0.796893
novel_circ_000054	XM_024984710.1	401	574	0.811181
novel_circ_000055	XM_005201597.4	351	524	0.749875
novel_circ_000056	XM_005201597.4	351	524	0.92844
novel_circ_000058	XM_015472368.2	51	224	0.694609
novel_circ_000059	XM_015472368.2	201	374	0.682075
novel_circ_000060	XM_015472368.2	1	174	0.826826
novel_circ_000061	XM_015472368.2	451	624	0.819211
novel_circ_000063	XM_015472368.2	251	424	0.727339
novel_circ_000064	XM_015472368.2	136	309	0.591865
novel_circ_000065	XM_024991737.1	1	174	0.77463
novel_circ_000067	XM_024990400.1	1	174	0.848859
novel_circ_000069	NM_001075420.2	51	224	0.687877
novel_circ_000071	NM_001075351.1	101	274	0.91119
novel_circ_000072	NM_001206149.1	1	174	0.559022
novel_circ_000073	XM_024979838.1	101	274	0.742801
novel_circ_000074	XM_024990577.1	301	474	0.860981
novel_circ_000076	XM_024992575.1	151	324	0.862159
novel_circ_000078	XM_024990996.1	101	274	0.915408
novel_circ_000079	XM_015473092.2	751	924	0.861486
novel_circ_000080	XM_024998582.1	51	224	0.921988
novel_circ_000081	XM_024998247.1	1	174	0.769281
novel_circ_000082	XM_024998166.1	1	174	0.769281
novel_circ_000084	XM_015473196.2	1	174	0.825012
novel_circ_000085	XM_024996179.1	1	174	0.624171
novel_circ_000087	XM_024996300.1	51	224	0.636635
novel_circ_000088	XM_024987430.1	151	324	0.715205
novel_circ_000089	XM_024996382.1	51	224	0.827014
novel_circ_000090	XM_024996382.1	151	324	0.86144
novel_circ_000091	XM_024996382.1	251	424	0.691505
novel_circ_000092	XM_024991344.1	401	574	0.830158
novel_circ_000093	XR_003034553.1	251	424	0.823141
novel_circ_000094	XR_003034553.1	201	374	0.889242
novel_circ_000095	XM_024994087.1	201	374	0.851983
novel_circ_000096	NM_001103174.2	701	874	0.852234
novel_circ_000097	NM_001103174.2	701	874	0.852234
novel_circ_000098	NM_001103174.2	251	424	0.825567
novel_circ_000099	XM_005201971.4	13	186	0.568687
novel_circ_001706	XM_024982094.1	751	924	0.905605
novel_circ_001707	XM_010817290.3	601	774	0.911524
novel_circ_001708	NM_001206397.1	601	774	0.899907
novel_circ_001709	XM_005222133.4	601	774	0.952197

novel_circ_001711	XM_010817306.3	601	774	0.860973
novel_circ_001712	XM_024981613.1	601	774	0.827646
novel_circ_001713	XM_024981789.1	351	524	0.86281
novel_circ_001714	NM_001038224.2	48	221	0.502485
novel_circ_001715	NM_001038224.2	151	324	0.704202
novel_circ_001716	XM_024982146.1	201	374	0.836838
novel_circ_001717	XM_024982146.1	351	524	0.84402
novel_circ_001718	XM_024982157.1	251	424	0.624095
novel_circ_001720	XM_024982183.1	73	246	0.587255
novel_circ_001724	XM_015459415.2	451	624	0.97123
novel_circ_001726	NM_001097570.1	1	174	0.878276
novel_circ_001727	XM_024982789.1	51	224	0.816331
novel_circ_001728	XM_024982789.1	151	324	0.891835
novel_circ_001729	XM_024982789.1	151	324	0.785253
novel_circ_001731	XM_002696926.6	352	525	0.879869
novel_circ_001733	NM_001076524.1	351	524	0.845877
novel_circ_001736	NM_001102169.1	701	874	0.915303
novel_circ_001738	NM_174841.2	351	524	0.633274
novel_circ_001739	XM_024982697.1	101	274	0.781592
novel_circ_001740	XM_024982697.1	101	274	0.781592
novel_circ_001741	XM_024982697.1	101	274	0.781592
novel_circ_001748	XM_005222766.4	1	174	0.906163
novel_circ_001749	XR_239403.4	201	374	0.706768
novel_circ_001750	XR_239403.4	51	224	0.764992
novel_circ_001751	XM_010817698.3	80	253	0.801972
novel_circ_001752	XM_005223006.4	101	274	0.558545
novel_circ_001754	XR_814565.3	951	1124	0.745273
novel_circ_001755	XM_010817854.2	401	574	0.779616
novel_circ_001756	XM_002697034.5	101	274	0.833097
novel_circ_001757	XM_015459535.2	51	224	0.824415
novel_circ_001767	XM_024983314.1	1051	1224	0.522254
novel_circ_001769	XM_010818002.3	1	174	0.593712
novel_circ_001770	XM_010818002.3	1	174	0.593712
novel_circ_001771	NM_001079594.2	51	224	0.727871
novel_circ_001772	XM_024983749.1	466	639	0.672142
novel_circ_001773	NM_001206906.1	246	419	0.672142
novel_circ_001775	XM_010818054.3	337	510	0.792722
novel_circ_001776	XM_024983983.1	101	274	0.651994
novel_circ_001778	XM_015459754.2	1301	1474	0.908276
novel_circ_001779	XM_015459754.2	1301	1474	0.908276
novel_circ_001780	XM_024983830.1	1851	2024	0.908276
novel_circ_001781	XM_024983830.1	1	174	0.826223
novel_circ_001784	XM_024983954.1	1	174	0.563099
novel_circ_001785	XM_024983725.1	201	374	0.819541
novel_circ_001786	XM_010818199.3	1	174	0.755507
novel_circ_001787	XM_005223375.4	101	274	0.594575
novel_circ_001788	XM_024983638.1	351	524	0.771308

novel_circ_001789	XM_024983638.1	101	274	0.75316
novel_circ_001790	XM_024983638.1	1	174	0.730608
novel_circ_001791	NM_001076911.1	1	174	0.525252
novel_circ_001792	XM_024983643.1	201	374	0.753434
novel_circ_001794	XM_024983464.1	293	466	0.584548
novel_circ_001796	NM_001101903.1	51	224	0.759138
novel_circ_001797	XM_024983697.1	101	274	0.832564
novel_circ_001798	NM_001205754.1	351	524	0.87878
novel_circ_001801	NM_001080255.1	251	424	0.948863
novel_circ_001802	NM_001080255.1	251	424	0.948863
novel_circ_001803	XM_024984556.1	51	224	0.623672
novel_circ_001804	NM_001099067.1	1	174	0.666635
novel_circ_001805	XM_010818685.3	151	324	0.794002
novel_circ_001806	XM_024984539.1	351	524	0.68946
novel_circ_001808	NM_001206030.1	751	924	0.738286
novel_circ_001809	XM_024984367.1	351	524	0.891031
novel_circ_001810	XM_024984367.1	601	774	0.850984
novel_circ_001811	XM_024984367.1	101	274	0.899996
novel_circ_001812	XM_005224246.4	1	174	0.683967
novel_circ_001813	XM_005224246.4	1	174	0.683967
novel_circ_001814	XM_005224246.4	301	474	0.685494
novel_circ_001816	XM_005224173.4	101	274	0.651334
novel_circ_001817	XM_003587790.5	551	724	0.94791
novel_circ_001818	XM_003587790.5	101	274	0.933258
novel_circ_001819	XM_024984532.1	451	624	0.861986
novel_circ_001820	XM_024984532.1	351	524	0.848531
novel_circ_001822	XM_005224250.3	401	574	0.876078
novel_circ_001825	XM_024984399.1	201	374	0.605721
novel_circ_001826	XM_002697836.6	301	474	0.683544
novel_circ_001828	XM_024984572.1	2801	2974	0.788688
novel_circ_001829	XM_024984572.1	451	624	0.807193
novel_circ_001830	XM_024984528.1	201	374	0.727973
novel_circ_001831	XM_024985289.1	401	574	0.679573
novel_circ_001835	XM_005224670.4	51	224	0.76778
novel_circ_001837	XM_010819082.2	138	311	0.788748
novel_circ_001840	XM_024984647.1	351	524	0.568193
novel_circ_001842	XM_024984647.1	1	174	0.615345
novel_circ_001844	XM_010819325.3	101	274	0.932959
novel_circ_001845	XM_002698023.3	151	324	0.907324
novel_circ_001847	NM_001304554.1	950	1123	0.818653
novel_circ_001848	XM_024985304.1	51	224	0.828619
novel_circ_001852	XM_005224925.4	351	524	0.691195
novel_circ_001853	XM_005224894.3	1	174	0.760766
novel_circ_001856	NM_001192753.1	501	674	0.553035
novel_circ_001857	NM_001192753.1	551	724	0.686594
novel_circ_001859	XM_003587856.5	51	224	0.897627
novel_circ_001860	XM_005225169.4	1	174	0.712061

novel_circ_001862	XM_024985162.1	1	174	0.585297
novel_circ_001864	XM_024985725.1	29	202	0.706366
novel_circ_001865	NM_001035462.1	51	224	0.78793
novel_circ_001866	NM_001205581.1	151	324	0.777309
novel_circ_001867	XM_005225411.4	180	353	0.628576
novel_circ_001869	XM_024986072.1	201	374	0.777569
novel_circ_001871	XM_024986069.1	51	224	0.864302
novel_circ_001873	XM_010819775.3	751	924	0.902535
novel_circ_001874	XR_815635.3	351	524	0.672013
novel_circ_001875	XR_815635.3	1	174	0.887596
novel_circ_001876	XR_815635.3	151	324	0.884335
novel_circ_001877	XR_815635.3	151	324	0.870461
novel_circ_001878	XM_015460634.2	351	524	0.820982
novel_circ_001879	NM_001193217.2	501	674	0.934712
novel_circ_001880	XM_024985871.1	101	274	0.692056
novel_circ_001881	NM_174021.2	151	324	0.755478
novel_circ_001882	XM_005225607.4	151	324	0.934724
novel_circ_001883	XM_005225607.4	451	624	0.962329
novel_circ_001884	XM_024985593.1	151	324	0.65416
novel_circ_001885	XM_024985593.1	151	324	0.65416
novel_circ_001886	XM_005225584.4	151	324	0.814631
novel_circ_001890	XM_010820037.3	1151	1324	0.595849
novel_circ_001891	XM_010820029.3	1	174	0.969676
novel_circ_001894	NM_001077948.1	101	274	0.894176
novel_circ_001895	XM_024985963.1	251	424	0.92168
novel_circ_001896	NM_001105411.1	251	424	0.74304
novel_circ_001898	XM_024986064.1	251	424	0.854573
novel_circ_001901	XM_024985640.1	330	503	0.73655
novel_circ_001902	XM_002698547.5	601	774	0.932329
novel_circ_001903	XM_010820119.3	2601	2774	0.562764
novel_circ_001907	XM_005225872.4	1	174	0.675831
novel_circ_001908	XM_005225872.4	1	174	0.675831
novel_circ_001909	XM_005225872.4	451	624	0.712003
novel_circ_001910	XM_005225872.4	1	174	0.637268
novel_circ_001911	XM_024986270.1	351	524	0.58274
novel_circ_001912	XM_024986270.1	51	224	0.582133
novel_circ_001916	XM_015460848.2	651	824	0.904445
novel_circ_001917	XM_015460848.2	51	224	0.656883
novel_circ_001919	XM_024986290.1	301	474	0.690217
novel_circ_001920	NM_001192992.2	1	174	0.598081
novel_circ_001921	NM_001192992.2	1201	1374	0.879525
novel_circ_001922	XM_010820287.3	1101	1274	0.949479
novel_circ_001923	XM_005225982.1	51	224	0.801819
novel_circ_001924	NM_001034312.1	231	404	0.836933
novel_circ_001925	XM_024986205.1	1	174	0.838704
novel_circ_001926	NM_001075598.2	351	524	0.923117
novel_circ_001929	XM_005226146.4	201	374	0.539085

novel_circ_001930	NM_001034764.1	78	251	0.532811
novel_circ_001931	NM_001034764.1	401	574	0.737158
novel_circ_001933	XM_024986264.1	51	224	0.780391
novel_circ_001934	XM_024986357.1	201	374	0.935131
novel_circ_001935	XM_024986287.1	1251	1424	0.78284
novel_circ_001936	XM_005226178.4	180	353	0.651611
novel_circ_001943	XM_024986813.1	101	274	0.535381
novel_circ_001944	NM_001206046.2	84	257	0.515848
novel_circ_001945	XR_003033146.1	351	524	0.525862
novel_circ_001947	XM_010820410.2	201	374	0.819513
novel_circ_001948	XM_024986498.1	201	374	0.819513
novel_circ_001949	XM_010820410.2	201	374	0.819513
novel_circ_001953	NM_001034635.1	301	474	0.643459
novel_circ_001954	NM_001034635.1	1	174	0.778993
novel_circ_001955	XM_024986503.1	36	209	0.619454
novel_circ_001956	XM_005226253.4	1	174	0.785042
novel_circ_001958	XM_024986626.1	301	474	0.843556
novel_circ_001959	XM_005226302.4	319	492	0.633642
novel_circ_001961	XM_005226412.3	433	606	0.884264
novel_circ_001962	XM_005226412.3	670	843	0.884264
novel_circ_001964	XM_005226412.3	201	374	0.792552
novel_circ_001965	XM_024986845.1	1	174	0.83059
novel_circ_001967	XM_024986773.1	601	774	0.793222
novel_circ_001968	XM_005226430.4	1	174	0.747055
novel_circ_001969	XM_024986797.1	51	224	0.552221
novel_circ_001971	XM_024986717.1	1	174	0.700751
novel_circ_001972	XM_024986717.1	1	174	0.691397
novel_circ_001973	XM_024986807.1	401	574	0.932188
novel_circ_001974	XM_005226500.3	601	774	0.832377
novel_circ_001979	XM_010820667.3	94	267	0.590299
novel_circ_001980	XM_010820667.3	101	274	0.607411
novel_circ_001986	NM_001076800.1	451	624	0.922267
novel_circ_001987	XM_024986904.1	1	174	0.784929
novel_circ_001988	XM_024986740.1	201	374	0.925047
novel_circ_001989	XM_005226586.4	251	424	0.843597
novel_circ_001990	NM_001192974.1	51	224	0.814558
novel_circ_001993	NM_001205767.1	1	174	0.741705
novel_circ_001995	NM_001034730.2	651	824	0.93903
novel_circ_001996	NM_001034730.2	451	624	0.92235
novel_circ_001997	NM_001034730.2	651	824	0.93903
novel_circ_001998	NM_001034730.2	651	824	0.93903
novel_circ_001999	NM_001034730.2	451	624	0.92235
novel_circ_002000	NM_001034730.2	651	824	0.93903
novel_circ_002001	NM_001034730.2	1	174	0.832594
novel_circ_002002	NM_001034730.2	1	174	0.832594
novel_circ_002004	XR_240567.4	101	274	0.523647
novel_circ_002005	XM_024987087.1	51	224	0.924374

novel_circ_002006	XM_024987087.1	51	224	0.924374
novel_circ_002007	XM_024987087.1	51	224	0.924374
novel_circ_002008	XM_024987087.1	51	224	0.924374
novel_circ_002009	XM_024987087.1	51	224	0.924374
novel_circ_002010	XM_024987087.1	51	224	0.924374
novel_circ_002011	XM_024987087.1	529	702	0.808103
novel_circ_002012	XM_024987087.1	151	324	0.922958
novel_circ_002013	XM_024987087.1	1	174	0.875127
novel_circ_002014	XM_015461131.2	436	609	0.925291
novel_circ_002015	XM_024987556.1	301	474	0.785793
novel_circ_002017	XM_024987007.1	280	453	0.807531
novel_circ_002018	XM_024987007.1	251	424	0.870904
novel_circ_002019	XM_024987007.1	101	274	0.796473
novel_circ_002020	XM_024986963.1	201	374	0.964971
novel_circ_002021	XM_024986963.1	201	374	0.964971
novel_circ_002022	XM_005226807.4	101	274	0.642595
novel_circ_002023	XM_005226791.4	251	424	0.569476
novel_circ_002025	XM_015461225.2	651	824	0.863406
novel_circ_002039	XM_024987357.1	651	824	0.83205
novel_circ_002040	XM_024987357.1	280	453	0.676804
novel_circ_002041	XM_024987126.1	77	250	0.875836
novel_circ_002044	NM_001354660.1	1	174	0.800255
novel_circ_002045	NM_001206067.1	1	174	0.856507
novel_circ_002046	NM_001076064.1	227	400	0.75928
novel_circ_002047	XM_024988009.1	251	424	0.828356
novel_circ_002050	XR_003033407.1	869	1042	0.695256
novel_circ_002051	XM_005227835.4	51	224	0.873691
novel_circ_002052	XM_005227892.4	1	174	0.524034
novel_circ_002053	XM_024988374.1	51	224	0.755639
novel_circ_002054	XM_024988374.1	51	224	0.755639
novel_circ_002055	XM_010821771.2	1	174	0.847323
novel_circ_002056	XM_010821771.2	51	224	0.63568
novel_circ_002057	XM_024988013.1	301	474	0.768741
novel_circ_002058	XM_010821806.2	301	474	0.784871
novel_circ_002059	XM_024988217.1	94	267	0.602834
novel_circ_002060	XM_002699937.6	301	474	0.866319
novel_circ_002061	XM_002699937.6	51	224	0.873771
novel_circ_002062	XM_024988203.1	2416	2589	0.928289
novel_circ_002063	XM_005227978.4	1451	1624	0.911088
novel_circ_002064	XM_005227978.4	501	674	0.894373
novel_circ_002067	XM_005227992.4	217	390	0.862895
novel_circ_002068	XM_024988424.1	351	524	0.630555
novel_circ_002069	NM_001098070.2	51	224	0.814615
novel_circ_002070	XM_005228048.4	301	474	0.817767
novel_circ_002071	XM_005228048.4	3601	3774	0.879981
novel_circ_002072	NM_001110004.1	751	924	0.775112
novel_circ_002074	NM_001110004.1	301	474	0.886779

novel_circ_002075	NM_001110004.1	451	624	0.886518
novel_circ_002076	XM_024988789.1	1	174	0.708931
novel_circ_002077	XM_015461707.2	51	224	0.74659
novel_circ_002078	XM_024988171.1	551	724	0.8125
novel_circ_002079	XM_005228254.4	1	174	0.689812
novel_circ_002080	XM_024987733.1	101	274	0.879288
novel_circ_002084	XM_005228292.4	1051	1224	0.902156
novel_circ_002085	XM_010822076.3	101	274	0.50745
novel_circ_002086	XM_010822076.3	101	274	0.50745
novel_circ_002090	XM_024988359.1	351	524	0.715135
novel_circ_002091	XM_024988359.1	201	374	0.756285
novel_circ_002092	XM_024988359.1	901	1074	0.787473
novel_circ_002093	XM_024988359.1	1	174	0.735954
novel_circ_002096	XM_024988359.1	201	374	0.779848
novel_circ_002097	XM_024988359.1	1	174	0.542054
novel_circ_002098	XM_024987738.1	51	224	0.644983
novel_circ_002099	XM_024988856.1	51	224	0.848241
novel_circ_002101	XM_005228422.4	151	324	0.677669
novel_circ_002102	XM_024988274.1	151	324	0.786744
novel_circ_000101	XM_024989492.1	1	174	0.706722
novel_circ_000102	NM_001076328.1	1	174	0.731405
novel_circ_000103	XR_233448.4	1639	1812	0.666465
novel_circ_000104	XM_005202085.4	401	574	0.947287
novel_circ_000115	XM_005202056.4	501	674	0.873098
novel_circ_000116	XM_005202082.2	110	283	0.611425
novel_circ_000117	XM_005202082.2	501	674	0.544507
novel_circ_000118	XM_024990067.1	151	324	0.659523
novel_circ_000119	XM_024990067.1	801	974	0.865122
novel_circ_000120	XM_024990067.1	101	274	0.682689
novel_circ_000121	NM_001100314.2	401	574	0.848144
novel_circ_000122	NM_001100314.2	401	574	0.848144
novel_circ_000127	XM_024991838.1	1	174	0.664226
novel_circ_000128	NM_001102040.2	1151	1324	0.737664
novel_circ_000129	NM_001102040.2	490	663	0.664194
novel_circ_000130	XM_024997590.1	1	174	0.777996
novel_circ_000131	XM_010800689.3	1	174	0.890517
novel_circ_000134	XM_002685231.5	201	374	0.937272
novel_circ_000136	XM_024979698.1	301	474	0.744719
novel_circ_000137	NM_001077981.2	265	438	0.82633
novel_circ_000138	XM_024975202.1	101	274	0.97293
novel_circ_000140	XM_024979808.1	219	392	0.843697
novel_circ_000141	XM_024978543.1	1	174	0.739464
novel_circ_000150	XM_024979882.1	51	224	0.509598
novel_circ_000156	XM_024979882.1	101	274	0.512631
novel_circ_000158	XM_024979882.1	51	224	0.84092
novel_circ_000162	XM_024979882.1	101	274	0.657169
novel_circ_000163	XM_024979882.1	1	174	0.539007

novel_circ_000170	XM_024979882.1	1	174	0.519964
novel_circ_000173	XM_024979882.1	1	174	0.572781
novel_circ_000175	XM_024979882.1	1	174	0.559136
novel_circ_000262	XM_010801849.3	401	574	0.679985
novel_circ_000263	XM_024979980.1	501	674	0.829665
novel_circ_000266	XM_024977517.1	271	444	0.844401
novel_circ_000267	XM_024977517.1	101	274	0.883992
novel_circ_000269	XM_024980246.1	101	274	0.875002
novel_circ_000270	NM_001102031.1	1082	1255	0.932394
novel_circ_000271	NM_001102031.1	429	602	0.957506
novel_circ_000274	XR_003030703.1	1	174	0.821767
novel_circ_000276	XM_005202471.4	201	374	0.823513
novel_circ_000277	XM_010801997.3	1	174	0.88594
novel_circ_000279	XM_024980659.1	40	213	0.77845
novel_circ_000281	XM_024980659.1	951	1124	0.714368
novel_circ_000283	XM_024980659.1	501	674	0.8521
novel_circ_000284	XM_024980659.1	1	174	0.698734
novel_circ_000286	XM_024980659.1	1401	1574	0.602258
novel_circ_000287	XM_024980659.1	1151	1324	0.554288
novel_circ_000288	XM_024980659.1	1701	1874	0.596191
novel_circ_000291	XM_024980659.1	367	540	0.567079
novel_circ_000293	XM_024976769.1	601	774	0.789123
novel_circ_000295	XM_005202444.4	351	524	0.931647
novel_circ_000297	NM_174227.3	251	424	0.721347
novel_circ_000298	NM_001101242.1	151	324	0.864026
novel_circ_000299	XM_024977435.1	1	174	0.689807
novel_circ_000300	XM_005202563.4	51	224	0.756444
novel_circ_000301	XM_005202563.4	1	174	0.528559
novel_circ_000302	XM_005202579.3	101	274	0.734971
novel_circ_000304	NM_001168704.1	351	524	0.937614
novel_circ_000305	NM_001168704.1	1	174	0.827355
novel_circ_000306	NM_001168704.1	1	174	0.827355
novel_circ_000307	NM_001191279.2	1201	1374	0.884825
novel_circ_000308	XM_024999354.1	201	374	0.787888
novel_circ_000309	XM_005202681.4	286	459	0.887292
novel_circ_000310	XM_024999390.1	401	574	0.909017
novel_circ_000311	XM_025000250.1	301	474	0.895004
novel_circ_000312	XM_005202713.4	551	724	0.746752
novel_circ_000313	XM_005202713.4	251	424	0.646048
novel_circ_000314	XM_015458833.2	251	424	0.96129
novel_circ_000315	XM_024976507.1	201	374	0.555932
novel_circ_000316	XM_003585764.5	101	274	0.846867
novel_circ_000317	XM_024981453.1	151	324	0.916072
novel_circ_000318	XM_024981453.1	151	324	0.916072
novel_circ_000319	XM_024981453.1	151	324	0.916072
novel_circ_000321	XM_010802318.3	1	174	0.870806
novel_circ_000322	XM_010802318.3	51	224	0.899617

novel_circ_000324	XM_024999420.1	1	174	0.843784
novel_circ_000325	NM_001205468.2	115	288	0.760585
novel_circ_000326	NM_001205468.2	201	374	0.7179
novel_circ_000327	NM_001205468.2	151	324	0.710636
novel_circ_000328	NM_001046542.2	288	461	0.820868
novel_circ_000330	XM_005202939.4	451	624	0.871891
novel_circ_000332	XM_025001187.1	2051	2224	0.860907
novel_circ_000333	XM_025001187.1	601	774	0.764189
novel_circ_000334	XM_025001187.1	901	1074	0.71897
novel_circ_000336	XM_024977589.1	451	624	0.978934
novel_circ_000337	XM_005202942.4	101	274	0.683
novel_circ_000338	NM_001205785.1	401	574	0.742426
novel_circ_000339	NM_174570.2	1	174	0.515755
novel_circ_000340	NM_001046035.2	1	174	0.64265
novel_circ_000341	XM_005203319.2	101	274	0.790386
novel_circ_000344	NM_001046332.1	301	474	0.768137
novel_circ_000345	XM_024982968.1	301	474	0.571691
novel_circ_000346	XM_024999272.1	51	224	0.512764
novel_circ_000347	XM_025001359.1	293	466	0.548351
novel_circ_000348	XM_005203374.4	201	374	0.764753
novel_circ_000349	XM_005203402.4	239	412	0.809844
novel_circ_000350	XM_015461056.2	359	532	0.809844
novel_circ_000351	XM_005203393.4	51	224	0.753372
novel_circ_000352	XM_024989365.1	767	940	0.84607
novel_circ_000353	XM_024989365.1	101	274	0.75877
novel_circ_000355	NM_001076158.1	444	617	0.729381
novel_circ_000357	XM_005203519.4	251	424	0.688897
novel_circ_000358	NM_001046404.1	1051	1224	0.847903
novel_circ_000359	XM_024989877.1	173	346	0.64795
novel_circ_000364	XM_010802923.3	2001	2174	0.944799
novel_circ_000365	XM_010802923.3	2001	2174	0.944799
novel_circ_000366	XM_010802923.3	201	374	0.780731
novel_circ_000367	XM_010802923.3	201	374	0.780731
novel_circ_000372	XM_024989703.1	1	174	0.803529
novel_circ_000373	XM_024989690.1	51	224	0.845641
novel_circ_000374	XM_024989748.1	1	174	0.935462
novel_circ_000376	XM_024989996.1	2451	2624	0.736923
novel_circ_000377	XM_024989992.1	101	274	0.736476
novel_circ_000381	NM_001206877.1	51	224	0.818272
novel_circ_000383	XM_024987693.1	951	1124	0.797923
novel_circ_000384	XM_024989548.1	751	924	0.973202
novel_circ_000385	XM_024989548.1	201	374	0.968735
novel_circ_000386	XM_024989470.1	1	174	0.766983
novel_circ_000387	XM_024989483.1	371	544	0.825576
novel_circ_000388	NM_174448.4	1	174	0.747441
novel_circ_000390	XR_003033859.1	151	324	0.804107
novel_circ_000391	XM_010803166.3	101	274	0.945217

novel_circ_000392	XM_005204117.4	551	724	0.920581
novel_circ_000393	XM_005204113.4	401	574	0.830853
novel_circ_000394	XM_024990064.1	51	224	0.920358
novel_circ_000395	XM_024985771.1	1	174	0.618665
novel_circ_000396	NM_001102485.1	199	372	0.869588
novel_circ_000397	XM_024985771.1	227	400	0.746438
novel_circ_000398	XM_024985771.1	251	424	0.770858
novel_circ_000399	XM_024985771.1	51	224	0.803044
novel_circ_000400	XM_024990069.1	1	174	0.811354
novel_circ_000401	XM_024990069.1	201	374	0.802825
novel_circ_000404	XM_024990074.1	101	274	0.579206
novel_circ_000406	NM_001082613.1	2251	2424	0.939397
novel_circ_000408	NM_001075993.1	129	302	0.74899
novel_circ_000409	NM_001192598.1	368	541	0.867973
novel_circ_000410	NM_001192598.1	241	414	0.867973
novel_circ_000411	XM_005204373.4	401	574	0.863281
novel_circ_000412	NM_001192943.2	501	674	0.827938
novel_circ_000413	XM_002686295.6	101	274	0.812404
novel_circ_000414	XM_024990106.1	201	374	0.921738
novel_circ_000415	XM_024990106.1	201	374	0.921738
novel_circ_000416	XM_024990106.1	51	224	0.883182
novel_circ_000417	XM_005204395.3	451	624	0.813949
novel_circ_000418	XM_010803398.3	101	274	0.810142
novel_circ_000420	XM_005204460.4	151	324	0.762403
novel_circ_000421	XM_005204460.4	1	174	0.888547
novel_circ_000422	XM_005204460.4	51	224	0.878798
novel_circ_000424	XM_005204465.4	400	573	0.500534
novel_circ_000425	XM_002686332.6	451	624	0.893711
novel_circ_000429	XM_005204520.4	151	324	0.711094
novel_circ_000430	XM_005204520.4	401	574	0.890537
novel_circ_000431	XM_005204520.4	601	774	0.93347
novel_circ_000432	XM_024989559.1	101	274	0.854353
novel_circ_000435	XM_002686348.5	551	724	0.812557
novel_circ_000436	XR_003033849.1	180	353	0.560113
novel_circ_000437	XR_003033849.1	151	324	0.580569
novel_circ_000438	XM_005204536.3	801	974	0.922565
novel_circ_000439	XM_024989604.1	379	552	0.778232
novel_circ_000440	NM_001205605.1	51	224	0.841427
novel_circ_000441	NM_001076473.2	1	174	0.532133
novel_circ_000442	XM_010803500.3	401	574	0.741059
novel_circ_000443	NM_001102247.1	401	574	0.884115
novel_circ_000444	XM_024989410.1	451	624	0.879889
novel_circ_000445	XM_024989410.1	451	624	0.879889
novel_circ_000446	XM_024989410.1	1351	1524	0.922448
novel_circ_000447	XM_024989410.1	51	224	0.88349
novel_circ_000448	XM_024990138.1	851	1024	0.857915
novel_circ_000449	XM_010803527.3	239	412	0.863601

novel_circ_000453	XM_024985422.1	251	424	0.60434
novel_circ_000455	XM_005204917.3	201	374	0.675507
novel_circ_000456	XM_005204917.3	1351	1524	0.909721
novel_circ_000457	XM_005204917.3	901	1074	0.920437
novel_circ_000458	XM_005204917.3	801	974	0.916245
novel_circ_000459	NM_001205939.1	151	324	0.875825
novel_circ_000460	XM_002686481.6	201	374	0.885508
novel_circ_000461	XM_005204865.3	401	574	0.906615
novel_circ_000463	XM_024990238.1	351	524	0.816852
novel_circ_000464	XM_015467201.2	501	674	0.624046
novel_circ_000466	XM_024990960.1	1	174	0.715152
novel_circ_000468	XM_024990530.1	701	874	0.630699
novel_circ_000469	XM_024990551.1	351	524	0.884181
novel_circ_000470	NM_001105413.2	51	224	0.901713
novel_circ_000471	NM_001105413.2	246	419	0.873299
novel_circ_000472	XM_024990772.1	251	424	0.69138
novel_circ_000474	XM_003585951.5	51	224	0.914806
novel_circ_000475	XM_003585951.5	51	224	0.914806
novel_circ_000476	XM_003585951.5	51	224	0.914806
novel_circ_000478	XM_005205239.4	401	574	0.608752
novel_circ_000479	XM_005205239.4	101	274	0.851942
novel_circ_000480	XM_024990889.1	151	324	0.716096
novel_circ_000482	XM_024990627.1	631	804	0.851059
novel_circ_000483	XM_024990627.1	651	824	0.882898
novel_circ_000484	NM_001278621.1	51	224	0.848632
novel_circ_000486	XR_003034239.1	451	624	0.950703
novel_circ_000487	XM_002686750.5	1	174	0.854006
novel_circ_000489	XM_024991323.1	169	342	0.803566
novel_circ_000490	XM_024991321.1	151	324	0.795667
novel_circ_000491	NM_001206458.1	165	338	0.653673
novel_circ_000492	XM_005205362.4	251	424	0.844194
novel_circ_000493	XM_005205362.4	51	224	0.782222
novel_circ_000494	XM_024991043.1	151	324	0.549921
novel_circ_000495	XM_003585967.5	251	424	0.896968
novel_circ_000496	XM_005205444.4	401	574	0.883999
novel_circ_000499	XM_015469121.2	1	174	0.775799
novel_circ_000501	XM_024990871.1	151	324	0.823175
novel_circ_000502	XM_002686809.6	101	274	0.763538
novel_circ_000506	XM_024990619.1	351	524	0.777181
novel_circ_000507	XM_024990619.1	212	385	0.525094
novel_circ_000508	XM_005205465.4	101	274	0.839315
novel_circ_000509	NM_001075558.1	1	174	0.821413
novel_circ_000510	XM_005205480.4	451	624	0.911769
novel_circ_000511	XM_005205480.4	1	174	0.867332
novel_circ_000512	XM_024991087.1	1	174	0.642738
novel_circ_000513	XM_024991087.1	449	622	0.809373
novel_circ_000517	XM_010804337.3	451	624	0.744613

novel_circ_000518	XM_010804337.3	201	374	0.751165
novel_circ_000519	XM_010804337.3	201	374	0.751165
novel_circ_000520	XM_005205612.4	101	274	0.805621
novel_circ_000521	XM_005205631.4	1	174	0.6301
novel_circ_000523	XR_003034399.1	101	274	0.856783
novel_circ_000524	XR_003034399.1	51	224	0.771154
novel_circ_000525	XR_003034399.1	151	324	0.874976
novel_circ_000526	XR_003034399.1	151	324	0.874976
novel_circ_000527	XM_015470366.2	151	324	0.874976
novel_circ_000528	XM_024990698.1	651	824	0.722381
novel_circ_000529	XM_005205644.4	301	474	0.870593
novel_circ_000530	XM_024990475.1	251	424	0.687814
novel_circ_000533	XM_005205713.4	259	432	0.793976
novel_circ_000534	NM_001034591.2	351	524	0.85674
novel_circ_000535	XM_002686941.6	51	224	0.84035
novel_circ_000537	XM_024990571.1	230	403	0.604779
novel_circ_000541	XM_024991189.1	151	324	0.911401
novel_circ_000542	XM_024991189.1	51	224	0.925812
novel_circ_000546	NM_001143871.1	175	348	0.794033
novel_circ_000550	XM_015470756.2	1	174	0.714902
novel_circ_000551	XM_002687138.4	107	280	0.912579
novel_circ_000553	XM_024991282.1	51	224	0.824345
novel_circ_000554	XM_005205980.4	119	292	0.706726
novel_circ_000555	XM_005205980.4	63	236	0.706726
novel_circ_000556	XM_005205980.4	151	324	0.616913
novel_circ_000557	XM_024990564.1	201	374	0.724689
novel_circ_000558	XM_024990564.1	51	224	0.755979
novel_circ_000559	XM_024990564.1	51	224	0.755979
novel_circ_000560	XM_024990564.1	201	374	0.666107
novel_circ_000561	XM_024990564.1	151	324	0.557681
novel_circ_000562	XM_005206023.4	151	324	0.760439
novel_circ_000563	XM_010804732.3	101	274	0.92773
novel_circ_000564	XM_024991845.1	1	174	0.757647
novel_circ_000565	XM_024991981.1	1795	1968	0.836083
novel_circ_000567	NM_001102137.2	292	465	0.781033
novel_circ_000570	XM_024991623.1	251	424	0.79613
novel_circ_000571	XM_005206128.4	151	324	0.534582
novel_circ_000573	NM_001075289.1	1	174	0.681358
novel_circ_000574	XM_024992004.1	201	374	0.801713
novel_circ_000575	XM_024992005.1	351	524	0.907446
novel_circ_000576	XM_024992006.1	401	574	0.9022
novel_circ_000577	XM_024991865.1	351	524	0.922509
novel_circ_000580	XM_002687323.6	1	174	0.834222
novel_circ_000581	XM_002687323.6	101	274	0.86738
novel_circ_000582	XM_005206409.4	101	274	0.862996
novel_circ_000583	XM_005206409.4	151	324	0.913808
novel_circ_000584	XM_005206409.4	201	374	0.869085

novel_circ_000586	XM_024991940.1	222	395	0.765516
novel_circ_000587	XM_024991940.1	201	374	0.670095
novel_circ_000588	XM_024991940.1	201	374	0.670095
novel_circ_000589	XM_024991940.1	251	424	0.801497
novel_circ_000590	XM_024991940.1	51	224	0.792226
novel_circ_000591	XM_005206425.4	225	398	0.683591
novel_circ_000592	XR_003034591.1	1	174	0.667901
novel_circ_000593	NM_001113261.1	201	374	0.925427
novel_circ_000594	XR_806676.3	51	224	0.918746
novel_circ_000595	XR_003034581.1	51	224	0.918746
novel_circ_000596	XM_005206467.4	1251	1424	0.81547
novel_circ_000597	XM_005206467.4	701	874	0.71511
novel_circ_000598	XM_002687544.5	1	174	0.688178
novel_circ_000599	XM_024992491.1	1	174	0.948561
novel_circ_000602	NM_001035333.2	51	224	0.787698
novel_circ_000603	XM_005206550.4	151	324	0.599071
novel_circ_000604	NM_001191507.1	190	363	0.739894
novel_circ_000605	NM_001191507.1	401	574	0.886594
novel_circ_000606	NM_001191507.1	151	324	0.94141
novel_circ_000607	XM_024991779.1	951	1124	0.942523
novel_circ_000608	XM_024991609.1	1	174	0.646058
novel_circ_000609	XM_024991609.1	401	574	0.735766
novel_circ_000610	XM_024991609.1	151	324	0.795404
novel_circ_000611	XM_024991609.1	151	324	0.795404
novel_circ_000612	XM_024991609.1	151	324	0.795404
novel_circ_000614	XM_024991609.1	201	374	0.674635
novel_circ_000615	XM_024991609.1	501	674	0.704611
novel_circ_000616	XM_024991609.1	101	274	0.652859
novel_circ_000617	XM_024991609.1	1	174	0.628895
novel_circ_000618	XM_024991609.1	1	174	0.606297
novel_circ_000619	XM_024991564.1	1	174	0.844591
novel_circ_000620	XM_024991564.1	1	174	0.844591
novel_circ_000621	XM_024991564.1	1	174	0.844591
novel_circ_000622	XM_024991564.1	195	368	0.857659
novel_circ_000623	NM_174625.4	551	724	0.855771
novel_circ_000624	XM_002687646.6	1	174	0.867173
novel_circ_000631	XM_005206853.4	351	524	0.882007
novel_circ_000632	XM_024992558.1	201	374	0.667382
novel_circ_000633	XM_024992558.1	401	574	0.725316
novel_circ_000634	XM_024992558.1	1	174	0.806951
novel_circ_000635	NM_001206120.1	551	724	0.883307
novel_circ_000636	XM_024991859.1	201	374	0.877901
novel_circ_000638	XM_024991354.1	101	274	0.557245
novel_circ_000639	XM_024991354.1	351	524	0.834644
novel_circ_000640	XM_024991354.1	151	324	0.951741
novel_circ_000641	XM_005206964.4	101	274	0.716674
novel_circ_000642	XM_005206964.4	601	774	0.941385

novel_circ_000643	XM_005206964.4	601	774	0.941385
novel_circ_000644	XM_002687752.5	51	224	0.67451
novel_circ_000645	XM_010805458.3	301	474	0.768404
novel_circ_000646	NM_001015567.2	351	524	0.867426
novel_circ_000647	XM_002687783.6	251	424	0.8452
novel_circ_000648	XM_002687783.6	51	224	0.785326
novel_circ_000649	XM_002687783.6	251	424	0.89831
novel_circ_000650	XM_002687783.6	1651	1824	0.916389
novel_circ_000653	XM_005207191.4	151	324	0.791306
novel_circ_000654	XM_024992728.1	201	374	0.857643
novel_circ_000655	XM_024992728.1	201	374	0.715206
novel_circ_000656	XM_024991758.1	51	224	0.610566
novel_circ_000659	XM_024992770.1	401	574	0.553985
novel_circ_000660	XM_010805721.3	251	424	0.562932
novel_circ_000662	XM_005207402.4	283	456	0.619978
novel_circ_000663	XM_005207402.4	382	555	0.860408
novel_circ_000665	XM_024991794.1	2551	2724	0.820129
novel_circ_000666	NM_001077051.2	601	774	0.799449
novel_circ_000667	NM_001163188.1	101	274	0.719579
novel_circ_000671	NM_001075652.2	1	174	0.894297
novel_circ_000672	XM_010805859.3	351	524	0.656118
novel_circ_000674	XM_005207588.4	311	484	0.763735
novel_circ_000676	XM_005207674.4	901	1074	0.918986
novel_circ_000677	XM_005207674.4	901	1074	0.918986
novel_circ_000679	XM_024993418.1	301	474	0.820975
novel_circ_000680	XM_010805945.3	301	474	0.885386
novel_circ_000681	XM_010805945.3	561	734	0.877921
novel_circ_000682	XM_010805945.3	201	374	0.867914
novel_circ_000685	XM_024993072.1	351	524	0.769877
novel_circ_000687	XM_005207750.4	451	624	0.712039
novel_circ_000688	XM_024993526.1	101	274	0.885377
novel_circ_000689	XM_024993526.1	101	274	0.885377
novel_circ_000690	XM_024993526.1	101	274	0.885377
novel_circ_000691	NM_177945.3	701	874	0.905078
novel_circ_000692	NM_001098018.1	201	374	0.901947
novel_circ_000694	XM_015471558.2	184	357	0.765767
novel_circ_000695	NM_001076938.1	251	424	0.933822
novel_circ_000696	XM_005207901.4	301	474	0.873668
novel_circ_000697	XM_024993548.1	601	774	0.967584
novel_circ_000700	XM_002688216.6	396	569	0.852423
novel_circ_000701	XM_002688216.6	140	313	0.852423
novel_circ_000702	XM_024993046.1	401	574	0.795796
novel_circ_000704	XM_024993301.1	251	424	0.857808
novel_circ_000705	XM_024993301.1	251	424	0.857808
novel_circ_000706	XM_024993354.1	399	572	0.896325
novel_circ_000707	NM_001102188.1	37	210	0.563385
novel_circ_000708	XM_024993205.1	429	602	0.754156

novel_circ_000709	XM_024993205.1	251	424	0.663591
novel_circ_000710	XM_010806136.3	151	324	0.577295
novel_circ_000712	XM_005208030.4	551	724	0.853664
novel_circ_000713	NM_001083438.2	101	274	0.894167
novel_circ_000716	XM_024993362.1	293	466	0.717822
novel_circ_000717	XM_005208070.4	51	224	0.917464
novel_circ_000721	XM_024993211.1	201	374	0.910704
novel_circ_000722	XM_024993211.1	201	374	0.910704
novel_circ_000723	NM_001206068.1	1	174	0.872349
novel_circ_000724	XM_024993248.1	51	224	0.855299
novel_circ_000725	XM_005208161.4	351	524	0.922842
novel_circ_000726	XM_015471693.2	351	524	0.922842
novel_circ_000728	XM_005208200.4	156	329	0.793656
novel_circ_000732	XM_005208232.4	151	324	0.531618
novel_circ_000734	XM_005208378.4	1	174	0.898144
novel_circ_000735	NM_001046369.1	201	374	0.843681
novel_circ_000736	NM_001099708.1	251	424	0.876817
novel_circ_000746	XM_024994747.1	1	174	0.626709
novel_circ_000748	XM_024994836.1	284	457	0.598406
novel_circ_000749	XM_024994872.1	51	224	0.648758
novel_circ_000750	XM_024994872.1	351	524	0.757261
novel_circ_000751	XM_024994103.1	51	224	0.628219
novel_circ_000752	XM_002688832.6	151	324	0.895177
novel_circ_000753	XM_005208907.4	401	574	0.787012
novel_circ_000754	XM_024995008.1	3201	3374	0.698834
novel_circ_000755	XM_024995051.1	451	624	0.903778
novel_circ_000756	XM_024995051.1	251	424	0.878384
novel_circ_000757	XM_024995051.1	701	874	0.907652
novel_circ_000758	XM_024995051.1	101	274	0.856365
novel_circ_000759	XM_005209087.3	51	224	0.878046
novel_circ_000760	XM_005209087.3	251	424	0.89862
novel_circ_000761	NM_001206000.1	462	635	0.844264
novel_circ_000762	NM_001206000.1	359	532	0.717232
novel_circ_000763	NM_001105329.2	51	224	0.899714
novel_circ_000764	NM_001105329.2	51	224	0.899714
novel_circ_000765	NM_001038519.1	101	274	0.831444
novel_circ_000766	XM_024993870.1	101	274	0.549923
novel_circ_000768	NM_001114192.2	151	324	0.617498
novel_circ_000769	NM_001077107.3	51	224	0.848979
novel_circ_000770	NM_001077107.3	51	224	0.848979
novel_circ_000771	XM_005209365.1	101	274	0.837571
novel_circ_000772	XM_024995194.1	1	174	0.78114
novel_circ_000773	XM_024994302.1	51	224	0.688544
novel_circ_000775	XM_005209442.4	151	324	0.689429
novel_circ_000776	NM_001206634.1	1039	1212	0.814794
novel_circ_000779	XM_002689290.5	301	474	0.771373
novel_circ_000783	NM_001035454.2	1	174	0.804886

novel_circ_000784	NM_001191263.2	1	174	0.544704
novel_circ_000787	XM_024994404.1	1101	1274	0.934203
novel_circ_000788	XM_015472353.2	1101	1274	0.934203
novel_circ_000789	XM_015472353.2	1051	1224	0.929792
novel_circ_000790	XM_010807593.3	1	174	0.691375
novel_circ_000791	XM_024994142.1	51	224	0.55296
novel_circ_000792	XM_024994238.1	1551	1724	0.947739
novel_circ_000794	XM_024995322.1	201	374	0.753174
novel_circ_000795	XM_024995322.1	151	324	0.839081
novel_circ_000796	XM_015472367.2	460	633	0.91556
novel_circ_000797	XM_015472367.2	651	824	0.891102
novel_circ_000799	XM_010807625.3	51	224	0.868889
novel_circ_000800	XM_002689410.5	301	474	0.629585
novel_circ_000801	XM_002689410.5	801	974	0.755714
novel_circ_000802	XM_024993933.1	1001	1174	0.856246
novel_circ_000803	XM_010807645.3	451	624	0.865525
novel_circ_000804	XM_010807645.3	51	224	0.918806
novel_circ_000806	XM_024995338.1	1	174	0.85148
novel_circ_000807	XM_024994407.1	51	224	0.580162
novel_circ_000809	XM_024994255.1	1101	1274	0.860472
novel_circ_000811	XM_005209800.4	261	434	0.885149
novel_circ_000812	XM_024996055.1	51	224	0.769339
novel_circ_000814	NM_001038120.1	1	174	0.843073
novel_circ_000815	XM_024995513.1	51	224	0.689136
novel_circ_000816	XM_002689565.6	701	874	0.932293
novel_circ_000817	XM_024995808.1	151	324	0.877433
novel_circ_000818	XM_010807784.3	951	1124	0.845702
novel_circ_000819	XM_005209927.4	201	374	0.925643
novel_circ_000820	NM_001081534.1	101	274	0.9282
novel_circ_000822	XM_024996097.1	151	324	0.763888
novel_circ_000823	XM_024995852.1	1201	1374	0.845264
novel_circ_000824	XM_024995852.1	951	1124	0.858621
novel_circ_000825	XM_024995852.1	951	1124	0.858621
novel_circ_000826	XM_015472464.2	751	924	0.77968
novel_circ_000827	XM_024995922.1	1	174	0.765551
novel_circ_000828	XM_002689598.5	601	774	0.843159
novel_circ_000829	XM_002689598.5	601	774	0.843159
novel_circ_000830	XM_002689598.5	401	574	0.856043
novel_circ_000831	XM_015472474.2	101	274	0.835207
novel_circ_000833	XM_024996132.1	1	174	0.787651
novel_circ_000834	XR_003035878.1	351	524	0.884072
novel_circ_000835	XR_003035878.1	501	674	0.869764
novel_circ_000836	XM_024996151.1	1	174	0.841535
novel_circ_000837	NM_001102335.1	251	424	0.582732
novel_circ_000839	XM_005210026.4	51	224	0.795039
novel_circ_000841	XM_024995705.1	51	224	0.843241
novel_circ_000842	XM_024995705.1	1	174	0.845728

novel_circ_000845	XM_002689664.6	451	624	0.915082
novel_circ_000846	XM_002689664.6	101	274	0.933984
novel_circ_000847	XM_024996187.1	51	224	0.737492
novel_circ_000848	XM_024996187.1	665	838	0.819768
novel_circ_000849	XM_024996187.1	251	424	0.95968
novel_circ_000850	XM_024996187.1	601	774	0.9305
novel_circ_000851	XM_024996187.1	201	374	0.925578
novel_circ_000852	XM_024995558.1	154	327	0.55189
novel_circ_000854	XM_024995785.1	251	424	0.647013
novel_circ_000856	XM_002689728.6	301	474	0.941122
novel_circ_000857	XM_015472543.2	151	324	0.858749
novel_circ_000858	XM_003586402.4	251	424	0.67276

Table S2

Sample1-10

Protein FD	Master	Accession	Description	Sum PEP Sc	Coverage	# Peptides	# PSMs	# Unique P
High	IsMasterPr	novel_circ_	novel_circ_001953	25.63497	42.98643	9	73	9
High	IsMasterPr	novel_circ_	novel_circ_000771	24.75738	27.71084	5	31	5
High	IsMasterPr	novel_circ_	novel_circ_000674	19.21632	37.15847	5	29	5
High	IsMasterPr	novel_circ_	novel_circ_000948	19.17149	19.9005	3	28	3
High	IsMasterPr	novel_circ_	novel_circ_001780	16.83904	19.79876	10	20	10
High	IsMasterPr	novel_circ_	novel_circ_000612	15.28164	17.35219	10	11	4
High	IsMasterPr	novel_circ_	novel_circ_001954	13.31736	37.43842	3	14	3
High	IsMasterPr	novel_circ_	novel_circ_000609	12.23989	20.59448	7	7	1
High	IsMasterPr	novel_circ_	novel_circ_001264	10.22216	12.19512	2	13	2
High	IsMasterPr	novel_circ_	novel_circ_000286	9.017788	9.827116	5	6	5
High	IsMasterPr	novel_circ_	novel_circ_000281	8.835712	16.50335	6	7	6
High	IsMasterPr	novel_circ_	novel_circ_001977	6.848422	21.6	2	4	2
High	IsMasterPr	novel_circ_	novel_circ_000754	6.677653	14.20534	4	6	4
High	IsMasterPr	novel_circ_	novel_circ_001681	6.312475	8.51927	2	3	2
High	IsMasterPr	novel_circ_	novel_circ_000465	5.150294	15.26971	4	5	4
High	IsMasterPr	novel_circ_	novel_circ_000312	5.018184	12.53561	3	7	3
High	IsMasterPr	novel_circ_	novel_circ_001122	4.178089	23.33333	2	5	2
High	IsMasterPr	novel_circ_	novel_circ_001166	4.10808	16.26506	2	2	2
High	IsMasterPr	novel_circ_	novel_circ_000291	3.112461	13.53276	2	2	2
High	IsMasterPr	novel_circ_	novel_circ_000284	2.944405	13.12018	3	3	2
High	IsMasterPr	novel_circ_	novel_circ_000346	2.836204	25.92593	2	5	2
High	IsMasterPr	novel_circ_	novel_circ_001852	2.631618	10.96491	2	2	2
High	IsMasterPr	novel_circ_	novel_circ_000393	2.142848	5.128205	1	1	1
High	IsMasterPr	novel_circ_	novel_circ_001551	2.052076	16.04278	1	1	1
High	IsMasterPr	novel_circ_	novel_circ_000283	2.043946	11.93548	3	3	2
High	IsMasterPr	novel_circ_	novel_circ_000937	2.041197	14.15929	1	1	1
High	IsMasterPr	novel_circ_	novel_circ_000784	1.98464	10.29412	1	1	1
High	IsMasterPr	novel_circ_	novel_circ_001763	1.796152	7.027027	1	1	1
High	IsMasterPr	novel_circ_	novel_circ_000666	1.793714	4.779412	1	1	1
High	IsMasterPr	novel_circ_	novel_circ_000388	1.780486	15.625	2	2	2
High	IsMasterPr	novel_circ_	novel_circ_001816	1.689688	33.33333	2	2	2
High	IsMasterPr	novel_circ_	novel_circ_001559	1.666134	8.714597	2	2	2
High	IsMasterPr	novel_circ_	novel_circ_001911	1.624519	6.334842	1	2	1
High	IsMasterPr	novel_circ_	novel_circ_002067	1.561458	8.93617	1	1	1
High	IsMasterPr	novel_circ_	novel_circ_000961	1.42678	9.770115	1	1	1
High	IsMasterPr	novel_circ_	novel_circ_001973	1.400226	3.308063	1	1	1
High	IsMasterPr	novel_circ_	novel_circ_001787	1.382685	12.87129	1	3	1
High	IsMasterPr	novel_circ_	novel_circ_001903	1.202371	2.423559	2	2	2
High	IsMasterPr	novel_circ_	novel_circ_001633	0.980884	12.37113	1	1	1
High	IsMasterPr	novel_circ_	novel_circ_000938	0.901356	3.956044	1	2	1
High	IsMasterPr	novel_circ_	novel_circ_001864	0.861697	26.82927	1	2	1
High	IsMasterPr	novel_circ_	novel_circ_002092	0.858864	2.736318	1	1	1
Medium	IsMasterPr	novel_circ_	novel_circ_000895	0.68466	1.511111	1	1	1
Medium	IsMasterPr	novel_circ_	novel_circ_001228	0.678816	4.147465	1	1	1

Medium	IsMasterPr	novel_circ_	novel_circ_000290	0.66314	10.06098	1	1	1
Medium	IsMasterPr	novel_circ_	novel_circ_000717	0.628563	6.097561	1	1	1
Medium	IsMasterPr	novel_circ_	novel_circ_001921	0.609772	2.201835	1	2	1
Medium	IsMasterPr	novel_circ_	novel_circ_001404	0.549289	5.343511	1	1	1
Medium	IsMasterPr	novel_circ_	novel_circ_001731	0.543482	10.30596	1	1	1
Medium	IsMasterPr	novel_circ_	novel_circ_000288	0.539854	1.750547	1	1	1
Medium	IsMasterPr	novel_circ_	novel_circ_000818	0.521722	6.461087	1	1	1
Medium	IsMasterPr	novel_circ_	novel_circ_000665	0.519849	1.472135	1	1	1
Medium	IsMasterPr	novel_circ_	novel_circ_001218	0.500863	9.174312	1	1	1
Medium	IsMasterPr	novel_circ_	novel_circ_000893	0.464959	5.294118	1	1	1

Sample11-20

Protein FDI	Master	Accession	Description	Sum PEP S(	Coverage	# Peptides	# PSMs	# Unique P
High	IsMasterPr	novel_circ_	novel_circ_001953	31.54276	42.98643	9	23	9
High	IsMasterPr	novel_circ_	novel_circ_000948	28.19066	35.8209	5	39	5
High	IsMasterPr	novel_circ_	novel_circ_000291	25.52663	31.19658	4	10	4
High	IsMasterPr	novel_circ_	novel_circ_000312	22.84245	26.21083	5	9	5
High	IsMasterPr	novel_circ_	novel_circ_000674	20.47385	30.05464	4	25	4
High	IsMasterPr	novel_circ_	novel_circ_000609	15.15572	14.22505	5	8	1
High	IsMasterPr	novel_circ_	novel_circ_000612	14.78284	9.768638	6	9	2
High	IsMasterPr	novel_circ_	novel_circ_001852	14.53476	15.35088	2	5	2
High	IsMasterPr	novel_circ_	novel_circ_001903	13.2824	7.214316	5	9	5
High	IsMasterPr	novel_circ_	novel_circ_000281	13.07762	25.99278	9	11	9
High	IsMasterPr	novel_circ_	novel_circ_001780	12.6551	14.35336	7	11	7
High	IsMasterPr	novel_circ_	novel_circ_001977	11.46421	48	4	6	4
High	IsMasterPr	novel_circ_	novel_circ_000771	10.41389	18.9759	3	10	3
High	IsMasterPr	novel_circ_	novel_circ_001404	10.11619	21.37405	2	4	2
High	IsMasterPr	novel_circ_	novel_circ_001681	9.993681	8.51927	2	6	2
High	IsMasterPr	novel_circ_	novel_circ_000286	9.72494	17.65241	8	10	8
High	IsMasterPr	novel_circ_	novel_circ_001954	7.465542	27.58621	2	5	2
High	IsMasterPr	novel_circ_	novel_circ_001559	7.322939	23.96514	3	6	3
High	IsMasterPr	novel_circ_	novel_circ_000284	7.282942	24.4763	6	10	4
High	IsMasterPr	novel_circ_	novel_circ_000283	6.813183	22.90323	6	6	4
High	IsMasterPr	novel_circ_	novel_circ_000465	5.923014	14.60581	4	5	4
High	IsMasterPr	novel_circ_	novel_circ_001228	5.872074	16.12903	3	10	3
High	IsMasterPr	novel_circ_	novel_circ_000716	5.014835	13.33333	1	3	1
High	IsMasterPr	novel_circ_	novel_circ_001122	4.014425	21.66667	2	3	2
High	IsMasterPr	novel_circ_	novel_circ_001731	3.952043	21.90016	2	3	2
High	IsMasterPr	novel_circ_	novel_circ_000754	3.634169	4.219409	2	2	2
High	IsMasterPr	novel_circ_	novel_circ_001973	3.440796	6.202619	2	2	2
High	IsMasterPr	novel_circ_	novel_circ_000937	2.968538	14.15929	1	4	1
High	IsMasterPr	novel_circ_	novel_circ_000393	2.794525	5.128205	1	1	1
High	IsMasterPr	novel_circ_	novel_circ_001787	2.54806	11.88119	1	1	1
High	IsMasterPr	novel_circ_	novel_circ_000631	1.995907	14.81481	2	2	2
High	IsMasterPr	novel_circ_	novel_circ_000288	1.941504	3.063457	2	2	2
High	IsMasterPr	novel_circ_	novel_circ_000961	1.906578	9.770115	1	1	1
High	IsMasterPr	novel_circ_	novel_circ_001651	1.90518	13.88889	1	1	1
High	IsMasterPr	novel_circ_	novel_circ_000784	1.694264	17.64706	2	2	2

High	IsMasterPr	novel_circ_	novel_circ_000136	1.662341	9.42029	1	1	1
High	IsMasterPr	novel_circ_	novel_circ_001339	1.492832	8.264463	1	2	1
High	IsMasterPr	novel_circ_	novel_circ_000290	1.42784	21.95122	2	2	2
High	IsMasterPr	novel_circ_	novel_circ_001816	1.355168	16.66667	1	1	1
High	IsMasterPr	novel_circ_	novel_circ_000099	1.331987	16.74419	1	1	1
High	IsMasterPr	novel_circ_	novel_circ_002059	1.242756	11.14551	1	1	1
High	IsMasterPr	novel_circ_	novel_circ_001633	1.21056	12.37113	1	1	1
High	IsMasterPr	novel_circ_	novel_circ_001864	1.209012	26.82927	1	3	1
High	IsMasterPr	novel_circ_	novel_circ_000817	1.203495	11.15242	1	2	1
High	IsMasterPr	novel_circ_	novel_circ_000346	1.180259	12.96296	1	1	1
High	IsMasterPr	novel_circ_	novel_circ_000765	1.005551	3.163192	1	1	1
High	IsMasterPr	novel_circ_	novel_circ_001264	0.973467	3.252033	1	2	1
High	IsMasterPr	novel_circ_	novel_circ_001166	0.916856	6.024096	1	1	1
High	IsMasterPr	novel_circ_	novel_circ_001125	0.768021	7.898449	1	1	1
High	IsMasterPr	novel_circ_	novel_circ_001408	0.712198	9.090909	1	1	1
High	IsMasterPr	novel_circ_	novel_circ_000383	0.524474	6.839623	1	1	1
High	IsMasterPr	novel_circ_	novel_circ_000508	0.521289	9.278351	1	1	1
High	IsMasterPr	novel_circ_	novel_circ_001784	0.493089	19.93671	1	1	1
Medium	IsMasterPr	novel_circ_	novel_circ_001196	0.474955	13.63636	1	1	1
Medium	IsMasterPr	novel_circ_	novel_circ_001306	0.462937	16.0804	1	1	1
Medium	IsMasterPr	novel_circ_	novel_circ_000598	0.459796	8.653846	1	1	1
Medium	IsMasterPr	novel_circ_	novel_circ_000926	0.457424	7.833537	1	1	1
Medium	IsMasterPr	novel_circ_	novel_circ_000581	0.451365	19.11765	1	2	1
Medium	IsMasterPr	novel_circ_	novel_circ_001890	0.418619	2.066116	1	1	1
Medium	IsMasterPr	novel_circ_	novel_circ_000096	0.409939	1.052632	1	1	1

Sample21-30

Protein FDI	Master	Accession	Description	Sum PEP Sc	Coverage	# Peptides	# PSMs	# Unique P
High	IsMasterPr	novel_circ_	novel_circ_001953	44.8272	42.08145	9	62	9
High	IsMasterPr	novel_circ_	novel_circ_000612	34.04051	20.05141	12	19	4
High	IsMasterPr	novel_circ_	novel_circ_000948	29.67831	28.35821	4	38	4
High	IsMasterPr	novel_circ_	novel_circ_000609	29.23331	23.99151	9	17	1
High	IsMasterPr	novel_circ_	novel_circ_000674	27.61213	30.05464	4	49	4
High	IsMasterPr	novel_circ_	novel_circ_000771	26.17161	40.74074	6	38	6
High	IsMasterPr	novel_circ_	novel_circ_000281	23.34673	26.40536	9	15	9
High	IsMasterPr	novel_circ_	novel_circ_000312	21.2887	22.88699	4	10	4
High	IsMasterPr	novel_circ_	novel_circ_001977	19.73994	48	4	10	4
High	IsMasterPr	novel_circ_	novel_circ_001780	16.75883	18.73335	8	15	8
High	IsMasterPr	novel_circ_	novel_circ_000286	15.96028	18.38035	9	15	9
High	IsMasterPr	novel_circ_	novel_circ_000291	15.38271	31.19658	4	8	4
High	IsMasterPr	novel_circ_	novel_circ_001954	12.32378	37.43842	3	9	3
High	IsMasterPr	novel_circ_	novel_circ_001903	10.96975	5.579822	5	8	5
High	IsMasterPr	novel_circ_	novel_circ_000288	10.81173	12.83735	8	9	8
High	IsMasterPr	novel_circ_	novel_circ_001814	10.08911	9.247507	2	6	2
High	IsMasterPr	novel_circ_	novel_circ_000465	9.95557	20.24896	5	8	5
High	IsMasterPr	novel_circ_	novel_circ_000284	8.558525	21.16869	5	8	4
High	IsMasterPr	novel_circ_	novel_circ_001681	8.314596	11.56187	4	5	4
High	IsMasterPr	novel_circ_	novel_circ_000283	7.555776	16.12903	4	5	3

High	IsMasterPr	novel_circ_	novel_circ_001911	7.194022	14.02715	2	4	2
High	IsMasterPr	novel_circ_	novel_circ_001852	6.075716	14.91228	2	4	2
High	IsMasterPr	novel_circ_	novel_circ_000346	5.701159	25.92593	2	4	2
High	IsMasterPr	novel_circ_	novel_circ_000336	5.419131	15.95745	1	2	1
High	IsMasterPr	novel_circ_	novel_circ_001559	5.297544	27.45098	4	7	4
High	IsMasterPr	novel_circ_	novel_circ_001122	4.500315	23.33333	2	2	2
High	IsMasterPr	novel_circ_	novel_circ_001264	4.345561	12.19512	2	4	2
High	IsMasterPr	novel_circ_	novel_circ_000937	4.151129	19.02655	2	6	2
High	IsMasterPr	novel_circ_	novel_circ_000731	4.121494	10.74766	1	2	1
High	IsMasterPr	novel_circ_	novel_circ_001338	4.094852	11.92053	1	1	1
High	IsMasterPr	novel_circ_	novel_circ_000393	4.064791	8.058608	2	2	2
High	IsMasterPr	novel_circ_	novel_circ_001404	3.940819	16.41221	2	2	2
High	IsMasterPr	novel_circ_	novel_circ_000646	3.880744	10.28571	1	1	1
High	IsMasterPr	novel_circ_	novel_circ_000864	3.497027	9.105691	1	1	1
High	IsMasterPr	novel_circ_	novel_circ_001166	3.451494	20.33133	3	4	3
High	IsMasterPr	novel_circ_	novel_circ_001637	3.353106	4.035608	1	1	1
High	IsMasterPr	novel_circ_	novel_circ_001892	3.283079	7.845579	1	1	1
High	IsMasterPr	novel_circ_	novel_circ_000388	3.257829	14.375	2	2	2
High	IsMasterPr	novel_circ_	novel_circ_000754	3.237321	1.054852	1	1	1
High	IsMasterPr	novel_circ_	novel_circ_001731	3.232894	11.5942	1	3	1
High	IsMasterPr	novel_circ_	novel_circ_000716	3.132591	13.33333	1	1	1
High	IsMasterPr	novel_circ_	novel_circ_000442	3.085392	8.61244	1	1	1
High	IsMasterPr	novel_circ_	novel_circ_001651	3.041858	23.14815	2	2	2
High	IsMasterPr	novel_circ_	novel_circ_000290	2.977871	21.95122	2	2	2
High	IsMasterPr	novel_circ_	novel_circ_000784	2.942334	10.29412	1	2	1
High	IsMasterPr	novel_circ_	novel_circ_001635	2.574303	7.792208	1	1	1
High	IsMasterPr	novel_circ_	novel_circ_001787	2.32781	11.88119	1	1	1
High	IsMasterPr	novel_circ_	novel_circ_001973	1.834459	3.308063	1	1	1
High	IsMasterPr	novel_circ_	novel_circ_000307	1.755598	1.89374	2	4	2
High	IsMasterPr	novel_circ_	novel_circ_000765	1.628194	3.163192	1	1	1
High	IsMasterPr	novel_circ_	novel_circ_001866	1.577246	6.857143	1	1	1
High	IsMasterPr	novel_circ_	novel_circ_001633	1.443818	12.37113	1	1	1
High	IsMasterPr	novel_circ_	novel_circ_001716	1.377993	4.528302	1	1	1
High	IsMasterPr	novel_circ_	novel_circ_000895	1.279014	1.511111	1	1	1
High	IsMasterPr	novel_circ_	novel_circ_001551	1.267365	16.04278	1	1	1
High	IsMasterPr	novel_circ_	novel_circ_001228	1.25189	4.147465	1	1	1
High	IsMasterPr	novel_circ_	novel_circ_001864	1.242908	26.82927	1	2	1
High	IsMasterPr	novel_circ_	novel_circ_000341	1.233438	18.60465	1	1	1
High	IsMasterPr	novel_circ_	novel_circ_000136	1.226433	9.42029	1	1	1
High	IsMasterPr	novel_circ_	novel_circ_002067	1.053351	7.659574	1	1	1
Medium	IsMasterPr	novel_circ_	novel_circ_001878	0.899629	3.539823	1	1	1
Medium	IsMasterPr	novel_circ_	novel_circ_001339	0.806319	8.264463	1	1	1
Medium	IsMasterPr	novel_circ_	novel_circ_000508	0.777804	9.278351	1	2	1
Medium	IsMasterPr	novel_circ_	novel_circ_001318	0.700711	3.52381	1	1	1
Medium	IsMasterPr	novel_circ_	novel_circ_000961	0.633577	5.747126	1	1	1
Medium	IsMasterPr	novel_circ_	novel_circ_001816	0.626169	16.66667	1	1	1
Medium	IsMasterPr	novel_circ_	novel_circ_001474	0.611189	5.493134	1	1	1

# Protein G	# AAs	MW [kDa]	calc. pI	Found in Sa	Modificatio	Area: F2: S	emPAI	Score Sequ	# Peptides
1	221	25.45	7.96	High		1.10E+09	4.817	286.9516	9
1	332	36.361	9.35	High		7.70E+08	2.981	87.27749	5
1	183	21.062	9.16	High		1.40E+09	5.813	81.32044	5
1	201	21.753	6.8	High		1.40E+08	4.878	89.10183	3
1	3379	356.025	9.38	High		2.90E+07	0.162	58.08154	10
1	778	86.73	6.95	High		1.40E+08	0.668	38.15682	10
1	406	46.713	4.78	High		7.70E+09	1.015	39.94772	3
1	471	52.028	5.14	High			0.778	26.02448	7
1	246	28.689	5.52	High		7.20E+07	1.31	49.09481	2
1	2198	253.732	8.63	High		2.20E+08	0.089	21.84507	5
1	1939	222.173	9.14	High		9.30E+07	0.108	23.0031	6
1	125	13.46	5.66	High		4.70E+08	1.783	14.79403	2
1	5688	558.384	9.19	High		1.40E+07	0.015	15.38714	4
1	493	53.486	9.35	High		9.40E+06	0.269	10.75688	2
1	1205	137.453	6.15	High		3.50E+08	0.139	17.37793	4
1	1053	112.367	6.38	High		3.90E+07	0.122	24.41499	3
1	120	13.74	4.81	High		6.40E+07	0.668	19.73513	2
1	664	72.54	6.4	High		3.60E+08	0.122	7.621025	2
1	702	81.53	7.33	High		6.10E+07	0.103	7.364018	2
1	907	105.421	8.98	High		1.00E+08	0.11	8.296913	3
1	108	12.448	4.94	High		1.90E+08	1.371	17.20029	2
1	228	25.366	6.4	High		5.10E+07	0.359	6.07777	2
1	273	31.489	8.44	High		3.70E+07	0.122	4.06284	1
1	374	41.185	8.7	High		6.90E+06	0.086	4.005305	1
1	1240	144.147	8.98	High		5.70E+06	0.079	8.393015	3
1	226	25.412	5.08	High		6.40E+06	0.179	4.40521	1
1	136	15.392	10.7	High		2.20E+08	0.292	3.406537	1
1	185	19.615	5.24	High		1.00E+07	0.233	3.549939	1
1	272	30.168	5.35	High			0.145	3.996771	1
1	160	18.033	4.79	High		3.40E+07	0.585	6.333377	2
1	66	7.728	4.88	High		2.20E+07	1.512	5.802266	2
1	918	97.288	6.01	High		5.10E+08	0.125	4.920475	2
1	221	24.554	5.31	High		3.00E+07	0.179	6.283801	1
1	470	54.331	9.41	High		3.00E+05	0.093	3.423254	1
1	174	19.525	8.15	High			0.233	3.746787	1
1	1451	167.498	5.12	High			0.031	3.137367	1
1	101	11.534	9.31	High		9.50E+06	0.389	12.04205	1
1	7097	728.778	4.48	High		2.00E+06	0.014	6.978355	2
1	97	10.827	5	High		5.30E+06	0.292	3.194897	1
1	455	52.04	7.88	High		6.80E+05	0.105	3.908993	1
1	41	4.456	9.58	High		4.60E+08	1.154	5.493787	1
1	402	46.349	5.22	High		3.10E+06	0.089	3.180914	1
1	1125	126.528	7.44	High		3.70E+07	0.031	4.441221	1
1	217	24.609	5.4	High		9.50E+06	0.166	2.715076	1

1	328	38.276	8.69	High		1.60E+08	0.105	2.809973	1
1	164	18.578	8.68	High			0.194	2.028086	1
1	1090	120.76	5.33	High		2.10E+07	0.037	3.647666	1
1	786	82.6	7.94	High		1.10E+06	0.075	2.922355	1
1	621	68.596	5.27	High		1.20E+07	0.053	2.895534	1
1	2742	324.484	9.19	High			0.012	3.687817	1
1	681	75.619	6.8	High		4.00E+05	0.077	0	1
1	7608	818.066	9.13	High		1.30E+08	0	3.549651	1
1	109	13.277	6.07	High			0.233	2.382688	1
1	680	74.832	5.08	High		1.30E+07	0.054	2.416063	1

# Protein G	# AAs	MW [kDa]	calc. pI	Found in Sa	Modificatio	Area: F3: S	emPAI	Score Sequ	# Peptides
1	221	25.45	7.96	High		4.50E+09	6.627	88.36076	9
1	201	21.753	6.8	High		1.20E+09	6.017	112.5904	5
1	702	81.53	7.33	High		1.90E+08	0.48	40.97857	4
1	1053	112.367	6.38	High		6.60E+07	0.359	36.89299	5
1	183	21.062	9.16	High		2.50E+09	4.623	81.04062	4
1	471	52.028	5.14	High		4.70E+07	0.638	26.53901	5
1	778	86.73	6.95	High		7.70E+07	0.431	27.31001	6
1	228	25.366	6.4	High		5.90E+07	1.154	21.6277	2
1	7097	728.778	4.48	High		3.90E+06	0.048	27.04244	5
1	1939	222.173	9.14	High		1.20E+08	0.186	35.31793	9
1	3379	356.025	9.38	High		4.00E+07	0.123	37.53885	7
1	125	13.46	5.66	High		4.80E+08	3.642	23.71651	4
1	332	36.361	9.35	High		1.60E+08	0.778	28.49569	3
1	786	82.6	7.94	High		1.20E+08	0.334	17.41715	2
1	493	53.486	9.35	High		5.20E+06	0.374	19.49301	2
1	2198	253.732	8.63	High		1.50E+08	0.12	36.52632	8
1	406	46.713	4.78	High		2.80E+09	0.492	18.31545	2
1	918	97.288	6.01	High		4.90E+08	0.266	20.68781	3
1	907	105.421	8.98	High		1.20E+08	0.233	26.28087	6
1	1240	144.147	8.98	High		1.40E+08	0.164	17.29615	6
1	1205	137.453	6.15	High		2.60E+08	0.139	16.58218	4
1	217	24.609	5.4	High		6.80E+07	0.848	30.06526	3
1	210	23.956	6.33	High		1.40E+07	0.638	12.72711	1
1	120	13.74	4.81	High		1.40E+08	0.668	10.89159	2
1	621	68.596	5.27	High		1.50E+07	0.108	9.438806	2
1	5688	558.384	9.19	High		3.90E+06	0.01	8.157372	2
1	1451	167.498	5.12	High			0.062	7.236717	2
1	226	25.412	5.08	High		6.40E+06	0.638	12.33591	1
1	273	31.489	8.44	High			0.122	3.620981	1
1	101	11.534	9.31	High		4.10E+07	0.389	4.026991	1
1	243	27.904	8.16	High		6.90E+06	0.292	7.119661	2
1	2742	324.484	9.19	High		8.40E+06	0.024	5.94477	2
1	174	19.525	8.15	High			0.233	3.392392	1
1	108	12.189	9.06	High			0.585	4.022835	1
1	136	15.392	10.7	High		9.70E+06	0.668	5.692228	2

1	138	15.772	5.15	High		6.80E+06	0.292	4.095231	1
1	121	13.549	8.57	High	Acetyl [N-T	4.80E+07	1.154	5.431015	1
1	328	38.276	8.69	High		2.60E+07	0.222	5.581012	2
1	66	7.728	4.88	High		3.30E+06	0.585	2.708946	1
1	215	25.033	5.07	High			0.179	3.28163	1
1	323	36.72	5.11	High		6.20E+06	0.116	4.236518	1
1	97	10.827	5	High		1.40E+08	0.292	2.666634	1
1	41	4.456	9.58	High		2.20E+08	1.154	8.863279	1
1	269	31.76	9.29	High			0	0	1
1	108	12.448	4.94	High		4.50E+08	0.334	2.699639	1
1	1391	154.364	5.16	High		3.60E+07	0.025	3.036629	1
1	246	28.689	5.52	High		6.20E+06	0.233	3.927496	1
1	664	72.54	6.4	High		5.20E+08	0.059	2.702236	1
1	709	81.826	6.86	High		1.50E+07	0.05	3.43586	1
1	275	30.671	9.03	High			0.129	2.943323	1
1	424	47.177	8.21	High		1.50E+08	0.116	2.319083	1
1	97	11.447	7.37	High		1.10E+06	0.468	1.682305	1
1	316	35.498	7.11	High		6.90E+06	0.129	2.952778	1
1	66	7.489	8.09	High		6.00E+07	0.468	2.171915	1
1	199	22.361	7.83	High		1.00E+07	0.212	0	1
1	104	11.542	8.76	High		7.00E+06	0.334	2.852504	1
1	817	91.822	5.97	High			0.05	3.094894	1
1	204	22.646	9.55	High			0.194	0	1
1	484	51.326	7.94	High			0.129	0	1
1	570	64.025	7.61	High		2.70E+07	0.075	0	1

# Protein G	# AAs	MW [kDa]	calc. pI	Found in Sa	Modificatio	Area: F4: S	emPAI	Score Sequ	# Peptides
1	221	25.45	7.96	High		6.70E+09	6.627	229.0673	9
1	778	86.73	6.95	High		3.00E+08	1.268	69.34248	12
1	201	21.753	6.8	High		9.50E+08	4.878	119.027	4
1	471	52.028	5.14	High		1.80E+08	2.162	62.56487	9
1	183	21.062	9.16	High		1.70E+09	5.813	127.4694	4
1	243	26.508	9	High	Acetyl [N-T	1.10E+09	3.87	105.7487	6
1	1939	222.173	9.14	High		1.90E+08	0.206	53.51027	9
1	1053	112.367	6.38	High		9.70E+07	0.359	41.41367	4
1	125	13.46	5.66	High		6.90E+08	6.743	36.43849	4
1	3379	356.025	9.38	High		6.40E+07	0.149	38.49086	8
1	2198	253.732	8.63	High		3.80E+08	0.153	48.81264	9
1	702	81.53	7.33	High		3.80E+08	0.342	30.52084	4
1	406	46.713	4.78	High		3.40E+09	0.65	24.80153	3
1	7097	728.778	4.48	High		3.40E+06	0.041	23.36381	5
1	2742	324.484	9.19	High		2.10E+08	0.111	28.37773	8
1	1103	119.954	7.81	High		2.00E+07	0.129	19.41834	2
1	1205	137.453	6.15	High		6.50E+08	0.176	28.35846	5
1	907	105.421	8.98	High		1.40E+08	0.191	23.19009	5
1	493	53.486	9.35	High		1.80E+07	0.487	14.82915	4
1	1240	144.147	8.98	High		1.00E+08	0.135	17.31586	4

1	221	24.554	5.31	High		8.30E+07	0.638	10.86409	2
1	228	25.366	6.4	High		7.00E+06	0.585	11.00874	2
1	108	12.448	4.94	High		1.00E+08	1.371	13.27529	2
1	94	10.784	8.32	High	Acetyl [N-T	5.50E+07	0.778	7.83643	1
1	918	97.288	6.01	High		2.60E+08	0.266	11.1137	4
1	120	13.74	4.81	High		1.70E+08	0.668	7.359909	2
1	246	28.689	5.52	High		6.30E+07	0.52	14.39273	2
1	226	25.412	5.08	High		1.40E+08	0.931	15.33726	2
1	214	22.729	9.61	High			0.359	7.909216	1
1	151	16.025	9.17	High		1.10E+07	0.259	5.508084	1
1	273	31.489	8.44	High		3.10E+07	0.259	6.144358	2
1	786	82.6	7.94	High		9.30E+06	0.155	9.250655	2
1	175	19.63	5.39	High		1.20E+07	0.194	5.234268	1
1	615	64.553	4.64	High		4.60E+06	0.212	4.287913	1
1	664	72.54	6.4	High		2.60E+08	0.189	11.26215	3
1	1685	195.669	7.44	High			0.021	4.080487	1
1	803	91.425	8.98	High		1.80E+07	0.039	4.239556	1
1	160	18.033	4.79	High		8.10E+07	0.585	5.98613	2
1	5688	558.384	9.19	High		1.80E+07	0.005	4.104012	1
1	621	68.596	5.27	High		1.40E+07	0.108	10.0665	1
1	210	23.956	6.33	High		1.50E+07	0.179	3.894299	1
1	209	23.516	6.52	High		1.30E+07	0.194	3.970449	1
1	108	12.189	9.06	High		7.60E+06	1.512	5.993039	2
1	328	38.276	8.69	High		3.00E+08	0.222	6.569183	2
1	136	15.392	10.7	High		7.00E+06	0.292	8.106822	1
1	616	67.673	5.27	High			0.122	3.428308	1
1	101	11.534	9.31	High		1.90E+08	0.389	3.856216	1
1	1451	167.498	5.12	High		6.40E+07	0.031	3.24708	1
1	3802	431.84	5.67	High		2.60E+08	0.022	7.793918	2
1	1391	154.364	5.16	High		3.00E+07	0.025	3.377034	1
1	700	77.573	7.05	High			0.054	3.214351	1
1	97	10.827	5	High		5.80E+08	0.292	2.544727	1
1	265	30.133	9.11	High		2.40E+06	0.136	3.165276	1
1	1125	126.528	7.44	High		2.40E+07	0.031	4.22328	1
1	374	41.185	8.7	High		4.90E+06	0.086	2.673064	1
1	217	24.609	5.4	High		9.10E+07	0.166	2.615482	1
1	41	4.456	9.58	High		4.00E+08	1.154	5.299049	1
1	645	71.34	9.95	High			0.059	0	1
1	138	15.772	5.15	High		1.90E+07	0.292	2.623612	1
1	470	54.331	9.41	High		2.20E+07	0.093	2.812286	1
1	1017	115.084	5.96	High		1.50E+07	0.047	2.216459	1
1	121	13.549	8.57	High	Acetyl [N-T	8.00E+07	0.468	2.907443	1
1	97	11.447	7.37	High		5.70E+06	0.468	4.638107	1
1	1050	113.9	6.09	High		4.80E+06	0.037	0	1
1	174	19.525	8.15	High			0.233	2.15177	1
1	66	7.728	4.88	High			0.585	0	1
1	801	90.7	5.64	High	Acetyl [N-T	2.10E+07	0.066	2.637607	1

Sequest HT

Sequest HT

Sequest HT

Table S3 Primers, siRNA and gRNA sequence

Name	Sequence
CircNEB-bos-clone	F: 5′- GCGCTTATACACAGAGGC-3′
	R: 5′- TTGTTCATGTTGATGGCG-3′
CircNEB-mus-clone	F: 5′- CTACAAGAACTACCTGCACC-3′
	R: 5′- TCTATGTCACTGACCAAGGT-3′
CircNEB-DL	F: 5′- ATCTATCGCCAGCCTCCAAAC -3′
	R: 5′- CGCTGTAGTTGATTCTGTTCT -3′
PCNA-bos	F: 5′- TCCAGAACAAGAGTATAGC-3′
	R: 5′- TACAACAGCATCTCCAAT-3′
β-Actin-bos	F: 5′- CTGGCATTGTCATGGACTCTG -3′
	R: 5′- GCTCGGCTGTGGTGGTAAA -3′
MyoG-bos	F: 5′- CAAATCCACTCCCTGAAA-3′
	R: 5′- GCATAGGAAGAGATGAACA-3′
Myhc-bos	F: 5′- TGCTCATCTCACCAAGTTCC-3′
	R: 5′- CACTCTTCACTCTCATGGACC-3′
MyoD-bos	F: 5′- ACGGCATGATGGACTACAGC-3′
	R: 5′- AGGCAGTCGAGGCTCGACA-3′
CyclinD1-bos	F: 5′-CCGTCCATGCGGAAGATC-3′
	R: 5′-CAGGAAGCGGTCCAGGTAG-3′
CDK2-bos	F: 5′-TTTGCTGAGATGGTGACCCG-3′
	R: 5′-TAACTCCTGGCCAAACCACC-3′
PCNA-mus	F: 5′-ACTCCGCCACCATGTTTGAGG-3′
	R: 5′-TCGCAGCGGTATGTGTCGAAG-3′
β-Actin-mus	F: 5′-CAAGAGAGGTATCCTGACCCT -3′
	R: 5′- TGATCTGGGTCATCTTTTCAC-3′
MyoG-mus	F: 5′-CCGTGGGCATGTAAGGTGTGT-3′
	R: 5′-ACTTTAGGCAGCCGCTGGTTG-3′
Myhc-mus	F: 5′-CCCGCTGGCCATGAACTACCT-3′
	R: 5′-CGGGCCGGATAGAGTTGTCAG-3′
MyoD-mus	F: 5′-CCGGAGTGGCAGAAAGTTAAG-3′
	R: 5′-GGCCTGTCAAGTCTATGTCCC-3′
CyclinD1-mus	F: 5′-CCCGCTGGCCATGAACTACCT-3′
	R: 5′-CGGGCCGGATAGAGTTGTCAG-3′
CDK2-mus	F: 5′-AGGTCCTCCGCTCCGAGTGTC-3′
	R: 5′-CGCCACAACTTCTCCCGTCAA-3′
Pax3-mus	F: 5′-TCGTCTCGCCTTCACCTGGAT-3′
	R: 5′-AGCCGCTGCGTGGGTAATTCT-3′
Pax7-mus	F: 5′-GGACGACGAGGAAGGAGACAA-3′
	R: 5′-CGGGTTCTGATTCCACATCTG-3′
GLB1-Rab	F: 5′-TACTTCCGCGTGCCCAGCTTC -3′
	R: 5′-CGTGAGCCAGCCGCAGAAACT -3′
ACTB-Rab	F: 5′-CCCGACGGCCAGGTCATCA -3′
	R: 5′-CGCCCGACAGCACCGTGTT -3′

MyoD-Rab	F: 5´-GATCAAGATGTCAACGTCGTGT-3´<br>CAGAGGTGATACCAGCCAATAA-3´
MyoG-Rab	F: 5´-GCAGCGCCATCCAGTACAT-3´<br>CTGGGTTGGGACCGAACTC-3´
Myhc-Rab	F: 5´-CGTACTCCGGCCTCTTCTG-3´<br>GCGATGGCGTAGATGTGTG-3´
PCNA-Rab	F: 5´-CCCGCCACCATGTTTGAGG-3´<br>CGCAGCGGTACGTGTCGAA-3´
CDK2-Rab	F: 5´-TACTTCAATGCCCGATTA-3´<br>R: 5´-ATTTGCGATAACAAGCTC-3´
CyclinD1-Rab	F: 5´-CAAGCACCCGCAAACCTT-3´<br>R: 5´-TCAGGGCATCGCAAGTGA-3´
TPM1-Rab	F: 5´-GAAGCCGATGTAGCGTCTCT-3´<br>R: 5´-GCTCGGCTTTCAATGACTTT-3´
SKP1-Rab	F: 5´-CGCTGTAGTGGCCTTGT-3´<br>R: 5´-ATGGTGTTCGGGTTTGA-3´
p21-Rab	F: 5´-AGAAGGGCGTGGATAGCAC-3´<br>R: 5´-TGCCCATCTCAGAGTCACC-3´
SKP1-bos	F: 5´-CAGTGGTGTGAGGAGAAGTGA-3´<br>R: 5´-GGGCCTTAAGTTTGGGAACTG-3´
p21-bos	F: 5´-CAGGGACGCGCATCAAATC-3´<br>R: 5´-CGGCGTCTCGGTGACAAAG-3´
p57-bos	F: 5´-GGTCGCTCATCTCTCAGCC-3´<br>R: 5´-GCCACGAATTTGGGGTTGAC-3´
p27-bos	F: 5´-CCCTGGGATAAGGAAGCGAC-3´<br>R: 5´-GTGGTTTACGTCTGACGCCT-3´
PI3K-bos	F: 5´-CAATCGGTGACTGTGTGGGA-3´<br>R: 5´-CTGATGGAGTGTGTGGCTGT-3´
Akt-bos	F: 5´-TCCCCCAGTTCTCCTACTCG-3´<br>R: 5´-TCCTCTCCATCCTGTGTTGG-3´
Si-SKP1-BOS-263	5´-GGGAUCAAGAAUUCCUGAATT-3´
Si-SKP1-BOS-73	5´-GUGACCAUCAAGACUAUGUTT-3´
KO circNEB-F-5'gRNA1	5´-AGUAGCCAAAGGUCUGCCUCG-3´
KO circNEB-F-5'gRNA2	5´-AACAGUGUUAAAGUAGCCAAG-3´
KO circNEB-F-3'gRNA1	5´-CUGCUGGUUUGCCUAAUACA-3´
KO circNEB-F-3'gRNA2	5´-CAAUACAACGAGCCUGUUUUC-3´

KO circNEB-R-5'gRNA1	5´-CAUUAAAUACCGCUGUUGACC-3´
KO circNEB-R-5'gRNA2	5´-CUGUUGACCAUACUCAUGAU-3´
KO circNEB-R-3'gRNA1	5´-CGGUACAUAUAGAUGUGUAU-3´
KO circNEB-R-3'gRNA2	5´-UGGAUUUGCUGAUAUAACCUG-3´
